# Engineered CD47 protects T cells for enhanced antitumour immunity

**DOI:** 10.1038/s41586-024-07443-8

**Published:** 2024-05-15

**Authors:** Sean A. Yamada-Hunter, Johanna Theruvath, Brianna J. McIntosh, Katherine A. Freitas, Frank Lin, Molly T. Radosevich, Amaury Leruste, Shaurya Dhingra, Naiara Martinez-Velez, Peng Xu, Jing Huang, Alberto Delaidelli, Moksha H. Desai, Zinaida Good, Roel Polak, Audre May, Louai Labanieh, Jeremy Bjelajac, Tara Murty, Zach Ehlinger, Christopher W. Mount, Yiyun Chen, Sabine Heitzeneder, Kristopher D. Marjon, Allison Banuelos, Omair Khan, Savannah L. Wasserman, Jay Y. Spiegel, Sebastian Fernandez-Pol, Calvin J. Kuo, Poul H. Sorensen, Michelle Monje, Robbie G. Majzner, Irving L. Weissman, Bita Sahaf, Elena Sotillo, Jennifer R. Cochran, Crystal L. Mackall

**Affiliations:** 1 Center for Cancer Cell Therapy, Stanford Cancer Institute, Stanford University School of Medicine, Stanford, CA USA; 2 Parker Institute for Cancer Immunotherapy, San Francisco, CA USA; 3 Cancer Biology Program, Stanford University School of Medicine, Stanford, CA USA; 4 Immunology Graduate Program, Stanford University School of Medicine, Stanford, CA USA; 5 Masters in Translational Research and Applied Medicine Program, Stanford University School of Medicine, Stanford, CA USA; 6 British Columbia Cancer Agency, Vancouver, British Columbia Canada; 7 Department of Biomedical Data Science, Stanford University School of Medicine, Stanford, CA USA; 8 Department of Medicine, Stanford University School of Medicine, Stanford, CA USA; 9 Princess Máxima Center for Pediatric Oncology, Utrecht, The Netherlands; 10 Department of Bioengineering, Stanford University, Stanford, CA USA; 11 Institute for Stem Cell Biology and Regenerative Medicine, Stanford, CA USA; 12 Program in Biophysics, Stanford University, Stanford, CA USA; 13 Medical Scientist Training Program, Stanford University, Stanford, CA USA; 14 Department of Neurology, Stanford University School of Medicine, Stanford, CA USA; 15 Neurosciences Program, Stanford University, Stanford, CA USA; 16 Stanford Cancer Institute, Stanford University School of Medicine, Stanford, CA USA; 17 Sylvester Comprehensive Cancer Center, University of Miami, Miami, FL USA; 18 Department of Pathology, Stanford University School of Medicine, Stanford, CA USA; 19 Department of Pediatrics, Stanford University School of Medicine, Stanford, CA USA; 20 Ludwig Center for Cancer Stem Cell Research and Medicine, Stanford University School of Medicine, Stanford, CA USA; 21 Department of Chemical Engineering, Stanford University, Stanford, CA USA

**Keywords:** Cancer immunotherapy, Immunotherapy

## Abstract

Adoptively transferred T cells and agents designed to block the CD47–SIRPα axis are promising cancer therapeutics that activate distinct arms of the immune system^1,2^. Here we administered anti-CD47 antibodies in combination with adoptively transferred T cells with the goal of enhancing antitumour efficacy but observed abrogated therapeutic benefit due to rapid macrophage-mediated clearance of T cells expressing chimeric antigen receptors (CARs) or engineered T cell receptors. Anti-CD47-antibody-mediated CAR T cell clearance was potent and rapid enough to serve as an effective safety switch. To overcome this challenge, we engineered the CD47 variant CD47(Q31P) (47_E_), which engages SIRPα and provides a ‘don’t eat me’ signal that is not blocked by anti-CD47 antibodies. TCR or CAR T cells expressing 47_E_ are resistant to clearance by macrophages after treatment with anti-CD47 antibodies, and mediate substantial, sustained macrophage recruitment to the tumour microenvironment. Although many of the recruited macrophages manifested an M2-like profile^3^, the combined therapy synergistically enhanced antitumour efficacy. Our study identifies macrophages as major regulators of T cell persistence and illustrates the fundamental challenge of combining T-cell-directed therapeutics with those designed to activate macrophages. It delivers a therapeutic approach that is capable of simultaneously harnessing the antitumour effects of T cells and macrophages, offering enhanced potency against solid tumours.

## Abstract (web summary)

Combination of TCR or CAR T cells expressing the engineered CD47 variant 47_E_ with anti-CD47 antibody therapy results in synergistic antitumour efficacy due to T cell resistance to clearance by macrophages, while maintaining macrophage recruitment into the tumour microenvironment.

## Main

Myeloid cells are the most plentiful immune cells within the tumour microenvironment (TME) and there has been great interest in therapeutically targeting them for antitumour effects^2^. Increased levels of tumour-associated macrophages (TAMs) associate with poorer outcomes in numerous studies, and some preclinical data demonstrate that reducing or eliminating TAMs enhances responses to chemotherapy and immunotherapy^4,5^. However, despite dozens of clinical studies testing agents such as CSF1R and CCR2 inhibitors to deplete TAMs and tumour-associated myeloid cells, a clear clinical benefit has not been demonstrated^2,4,5^. Alternatively, increased TAM density is correlated with improved clinical outcomes in some cancers^2^, and augmenting TAM phagocytic activity by blocking the CD47–SIRPα axis mediates antitumour effects in several preclinical models^6–9^. Clinical trials of CD47–SIRPα axis blockers demonstrated antitumour activity in some liquid tumours when combined with additional agents, but clinical evidence for single-agent activity or activity in solid cancers is lacking^10–12^. Thus, despite extensive effort, therapeutic approaches to target TAMs for clinical benefit are lacking.

## Anti-CD47 abrogates CAR T and TCR T cell efficacy

**Fig. 1:**
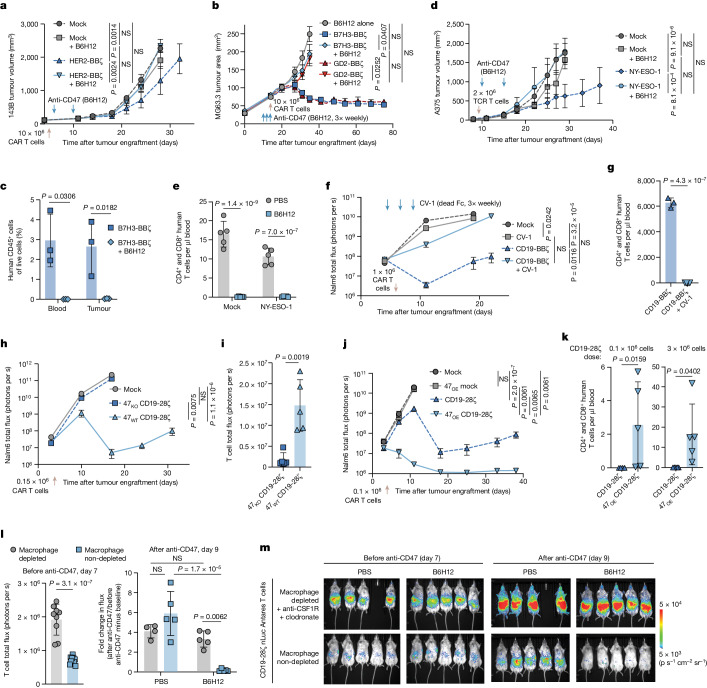
Anti-CD47 antibodies abrogate adoptively transferred T cell efficacy through macrophage-mediated T cell depletion. **a**, 143B osteosarcoma tumour growth after treatment with HER2-BBζ CAR T cells with or without B6H12. **b**, MG63.3 osteosarcoma tumour growth after treatment with B7H3-BBζ or GD2-BBζ CAR T cells with or without B6H12. **c**, T cells from blood and tumour in the MG63.3 model at day 30. Data are mean ± s.d. of *n* = 3 mice. **d**, A375 melanoma tumour growth after treatment with NY-ESO-1 TCR cells with or without B6H12. **e**, T cells in the blood of mice in the A375 model at day 17. **f**, Nalm6 tumour growth after treatment with CD19-BBζ CAR T cells with or without CV-1 (Fc-dead). **g**, T cells in the blood of mice in the Nalm6–CV-1 model at day 11. Data are mean ± s.d. of *n* = 3 (CD19-BBζ) or *n* = 4 (CD19-BBζ + CV-1) mice. **h**, Nalm6 leukaemia tumour growth after treatment with *CD47* knock out (47_KO_) CD19-28ζ CAR T cells. **i**, T cell BLI in the Nalm6–47_KO_ CAR T cell model at day 11. **j**, Nalm6 leukaemia tumour growth after treatment with CD47-overexpressing (47_OE_) CD19-28ζ CAR T cells. **k**, T cells on day 45 after CAR treatment in the blood of mice in the Nalm6–47_OE_ CAR T cell model. Data are mean ± s.d. of *n* = 4 (0.1 × 10^6^ CD19-28ζ) or *n* = 5 (all others) mice. **l**, T cell BLI after macrophage depletion (left). Data are mean ± s.d. of *n* = 9 (depleted) or *n* = 10 (non-depleted) mice. Right, the fold change in T cell BLI, with or without B6H12, after macrophage depletion. Data are mean ± s.d. of *n* = 4 (depleted + PBS) or *n* = 5 (all others) mice. **m**, T cell BLI before and after B6H12, after macrophage depletion. For **a**, **b**, **d**, **f**, **h** and **j**, data are mean ± s.e.m. of *n* = 5 mice per arm for tumour growth. For **e** and **i**, data are mean ± s.d. of *n* = 5 mice. Statistical analysis was performed using two-way analysis of variance (ANOVA) with Tukey’s multiple-comparison test (**a**, **b**, **d**, **f**, **h**, **j** and **l** (right)), unpaired two-tailed Student’s *t*-tests (**c**, **e**, **g**, **i**, **k** (right) and **l** (left)) and two-tailed Mann–Whitney *U*-tests (**k**, left); NS, not significant. Source Data

**Extended Data Fig. 1:**
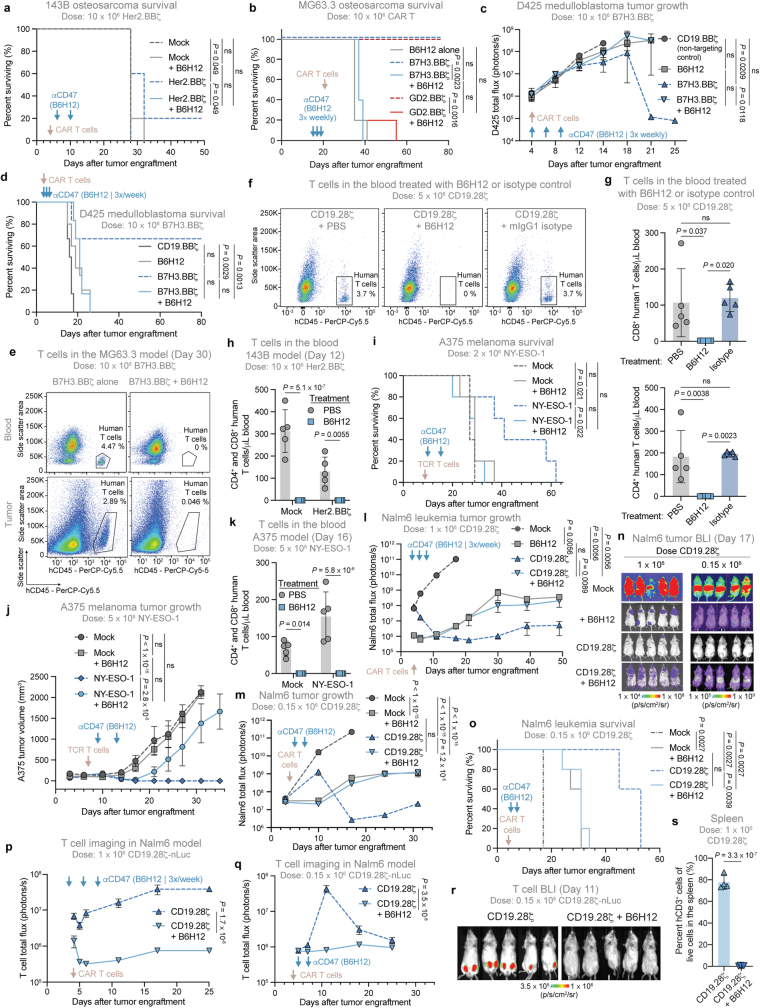
Anti-CD47 therapy blunts CAR and TCR T cell efficacy by depleting adoptively transferred T cells. (a) Her2.BBζ-CAR ± B6H12 treated 143B tumour survival. n = 5 mice/arm. (b) B7H3.BBζ- or GD2.BBζ-CAR ± B6H12 treated MG63.3 tumour survival. n = 5 mice/arm. (c) B7H3.BBζ-CAR ± B6H12 treated D425 tumour growth by BLI. CD19.BBζ-CAR is included as a non-tumour targeting control. Mean ± SEM of n = 5 (B6H12) or n = 6 (all others) mice/arm. Representative of two independent experiments. (d) B7H3.BBζ-CAR ± B6H12 treated D425 tumour survival. CD19.BBζ-CAR is included as a non-tumour targeting control. n = 5 (B6H12) or n = 6 (all others) mice per treatment arm. Representative of two independent experiments. (e) Representative flow cytometry plots of hCD45^+^ T cells identified in the blood and tumour in the B7H3.BBζ-CAR ± B6H12 treated MG63.3 model on day 30 post tumour engraftment. (f) Representative flow cytometry plots of hCD45^+^ T cells identified in the blood of non-tumour bearing mice co-treated with CD19.28ζ-CAR T cells and either PBS, B6H12, or mIgG1 isotype control. Representative of two independent experiments. (g) hCD8^+^ (top) and hCD4^+^ (bottom) T cells in the blood of mice on day 5 in the isotype control model, treated as in (f). Mean ± SD of n = 5 mice. Representative of two independent experiments. (h) T cells (hCD4^+^ and hCD8^+^) in the blood of mice on day 12 in Her2.BBζ ± B6H12 treated mice in the 143B model. Mean ± SD of n = 5 mice. (i) Low-dose NY-ESO-1-TCR ± B6H12 treated A375 tumour survival. n = 5 mice/arm. (j) High-dose NY-ESO-1-TCR ± B6H12 treated A375 tumour growth. Mean ± SEM of n = 5 mice/arm. (k) T cells (hCD4^+^ and hCD8^+^) in the blood by flow cytometry on day 16 of mice treated with high-dose NY-ESO-1-TCR ± B6H12 in the A375 model. Mean ± SD of n = 5 mice. (l) High-dose CD19.28ζ-CAR ± B6H12 treated Nalm6 tumour growth by BLI. Mean ± SEM of n = 5 mice/arm. (m) Low-dose CD19.28ζ-CAR ± B6H12 treated Nalm6 tumour growth by BLI. Mean ± SEM of n = 5 mice/arm. (n) Images of Nalm6 tumour BLI in the high-dose (left) and low-dose (right) CAR-T model on day 17. (o) Low-dose CD19.28ζ-CAR ± B6H12 treated Nalm6 survival. n = 5 mice/arm. (p) T cell BLI in the high-dose CAR-T – Nalm6 model, treated as in (l). Mean ± SEM of n = 5 mice/arm. (q) T cell BLI in the low-dose CAR-T – Nalm6 model, treated as in (m). Mean ± SEM of n = 5 mice/arm. (r) Images of CD19.28ζ-nLuc-CAR T cell BLI in the low-dose CAR T – Nalm6 model, treated as in (m), on day 11. (s) Quantification of T cells by flow cytometry from the spleen in the high-dose CAR T – Nalm6 model, treated as in (l). Mean ± SD of n = 4 mice. [(a), (b), (d), (i), (o)] Log-rank Mantel-Cox test. [(c), (j), (l), (m), (p), (q)] Two-way analysis of variance (ANOVA) test with Tukey’s multiple comparison test. [(g), (h), (k), (s)] Unpaired two-tailed Student’s *t* test. For all: ns = not significant. Source Data

To test the hypothesis that augmenting macrophage phagocytosis through CD47 blockade could improve efficacy of CAR T cell therapy, we administered HER2-BBζ CAR T cells with or without the anti-CD47 monoclonal antibody B6H12 to mice bearing 143B osteosarcoma xenografts. CAR T cells alone induced antitumour effects, but the addition of anti-CD47 antibodies ablated CAR T cell efficacy (Fig. 1a and Extended Data Fig. 1a). Similar antagonism was observed with MG63.3 osteosarcoma and D425 medulloblastoma (Fig. 1b and Extended Data Fig. 1b–d). To investigate the cause of therapeutic failure with dual treatment, we quantified human T cells in tumour-bearing mice after treatment with B7H3-BBζ CAR T cells ± B6H12. Human T cells were completely absent in the tumours and blood of MG63.3-bearing mouse recipients of B7H3-BBζ CAR T cells plus B6H12 but present in mice treated with CAR T cells only and isotype-control-treated animals (Fig. 1c and Extended Data Fig. 1e–g), and similar results were observed in the 143B model (Extended Data Fig. 1h). B6H12 also completely depleted adoptively transferred T cells expressing an NY-ESO-1-targeting TCR in mice bearing A375 melanoma xenografts and ablated their antitumour effects (Fig. 1d,e and Extended Data Fig. 1i–k).

To characterize the kinetics of CAR T cell depletion in B6H12 recipients, we used bioluminescence imaging (BLI) to monitor CD19-28ζ CAR T cells expressing nanoluciferase (CD19-28ζ-nLuc) in mice bearing Nalm6 leukaemia expressing firefly luciferase (Nalm6-fLuc). The modest single-agent efficacy of B6H12 in this system was not affected by co-administration of CD19-28ζ CAR T cells. However, B6H12 completely ablated CD19-28ζ CAR T cell efficacy and BLI demonstrated a significant loss of CAR T cell signal after B6H12 treatment, with T cells absent from the spleens of mice at the conclusion of the experiment (Extended Data Fig. 1l–s). Together, these data demonstrate that anti-CD47 antibodies induce rapid depletion of adoptively transferred T cells, including those engineered to express a transgenic TCR or CARs with differing targeting and costimulatory domains.

## CD47 is essential for CAR T cell persistence

**Extended Data Fig. 2:**
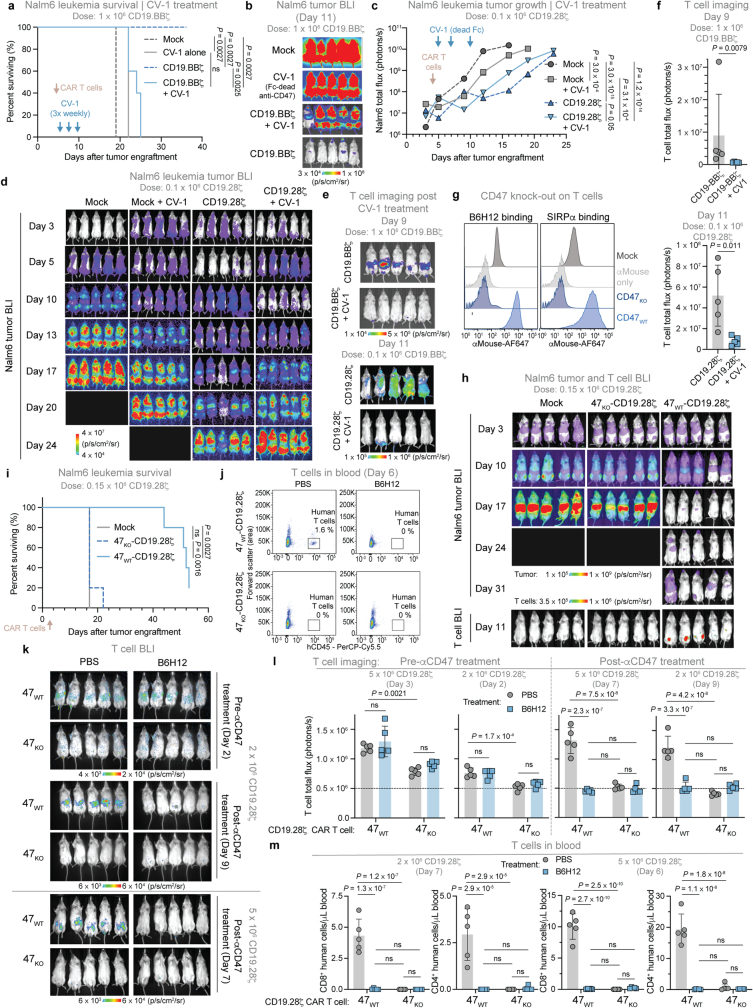
Treatment with CV-1 or CD47 knock-out leads to T cell depletion and ablation of antitumour efficacy. (a) CD19.BBζ-CAR ± CV-1 (Fc-dead) treated Nalm6 tumour survival. n = 5 mice/arm. (b) Images of Nalm6 BLI on day 11 of high-dose CD19.BBζ-CAR ± CV-1 treated mice, treated as in (a). (c) Low-dose CD19.28ζ-CAR ± CV-1 (Fc-dead) treated Nalm6 tumour growth. Mean ± SEM of n = 5 mice/arm. (d) Images of Nalm6 BLI of low-dose CD19.28ζ-CAR ± CV-1 treated mice, treated as in (c). (e - f) Images (e) and quantification (f) of T cell BLI post CV-1 treatment of Nalm6 tumour-bearing mice. Top: high-dose CD19.BBζ-CAR ± CV-1, treated as in (a), on day 9 (four days post CV-1 treatment). Bottom: low-dose CD19.28ζ-CAR ± CV-1, treated as in (c), day 11 (six days post CV-1 treatment). For (f): mean ± SD of n = 5 mice for each dose condition. (g) CRISPR/Cas9 mediated CD47 knock-out (CD47_KO_) efficiency in primary human T cells by flow cytometry. CD47_WT_ cells are CD47_KO_ with exogenous expression of wild-type CD47. Representative of n > 3 donors. (h) Images of Nalm6 tumour progression (top five rows) and T cells on day 11 (bottom row) by BLI in the Nalm6 – 47_KO_-CD19.28ζ-CAR T model. (i) Survival of Nalm6 bearing mice shown in (h). n = 5 mice per treatment arm. (j) Representative flow cytometry plots of hCD45^+^ T cells identified in the blood of 47_WT_- or 47_KO_-CD19.28ζ-nLuc-CAR ± B6H12 treated, non-tumour bearing mice. (k) Images of T cell BLI following treatment with 47_WT_- or 47_KO_-CD19.28ζ-nLuc-CAR T cells before and after B6H12 treatment. (l) T cell BLI of mice shown in (k) treated with 47_WT_- or 47_KO_-CD19.28ζ-nLuc-CAR T, before and after B6H12 treatment. Dashed line indicates limit of detection. Mean ± SD of n = 5 mice. (m) hCD8^+^ and hCD4^+^ T cells in the blood on day 6 or 7 by flow cytometry following treatment 47_WT_- or 47_KO_-CD19.28ζ-nLuc-CAR T ± B6H12 in separate experiments, treated as in (k). Mean ± SD of n = 5 mice. [(c), (l), (m)] Two-way analysis of variance (ANOVA) test with Tukey’s multiple comparison test. ns = not significant. [(f - top)] Two-tailed Mann-Whitney test. [(f - bottom)] Unpaired two-tailed Student’s *t* test. Source Data

To determine whether T cell ablation in these models occurs through FcR-mediated antibody-dependent phagocytosis^6^, we administered CV-1^9^, a fusion protein that binds to CD47 but does not bind to FcRs. Similar to the results with B6H12, CV-1 co-treatment with CD19 CAR T cells abrogated antitumour efficacy in Nalm6-fLuc-bearing mice, and induced near total T cell depletion (Fig. 1f,g and Extended Data Fig. 2a–f). We next tested whether CD47 expression was required for the survival of adoptively transferred T cells by using CRISPR-Cas9 to knock out *CD47* (47_KO_) in primary human T cells, and then restoring CD47 protein expression in 47_KO_ cells (47_WT_, Extended Data Fig. 2g). 47_WT_ CAR T cells expanded in Nalm6-fLuc mice and mediated robust tumour control and significantly prolonged survival, whereas 47_KO_ CAR T cells were depleted and delivered no anti-tumour activity (Fig. 1h,i and Extended Data Fig. 2h,i). Even in the absence of tumour, 47_WT_ CAR T cells robustly expanded, while 47_KO_ CAR T cells were depleted in vivo (Extended Data Fig. 2j–m).

**Extended Data Fig. 3:**
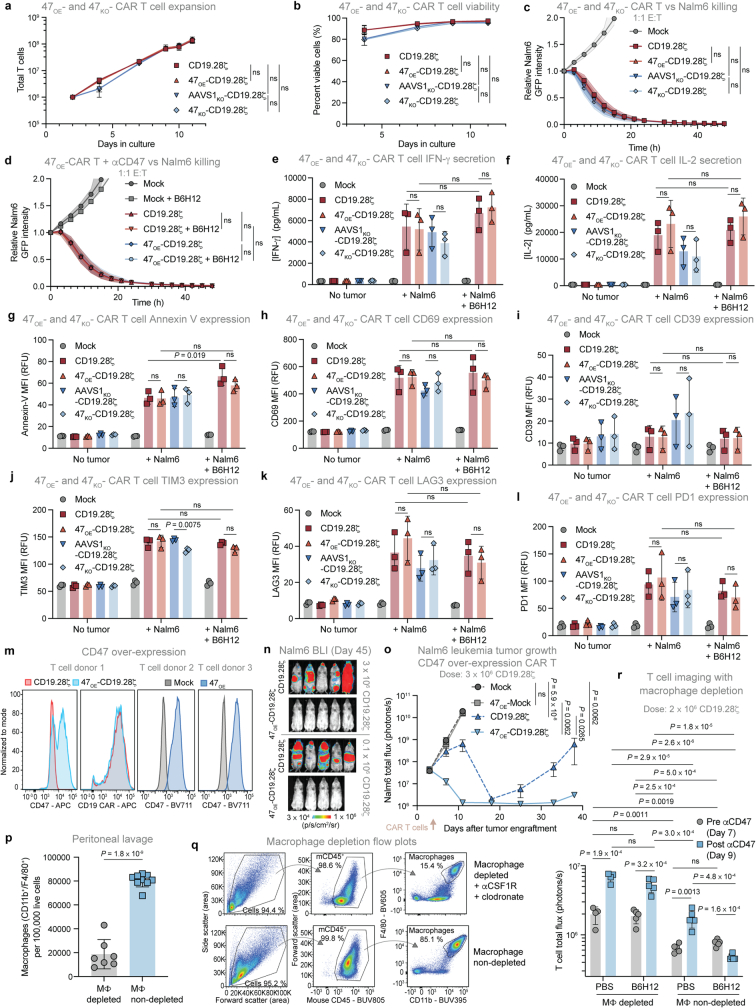
CD47 over-expression (47_OE_) does not alter T cell function in vitro, but enhances efficacy in vivo. (a) Expansion of CD19.28ζ-, 47_OE_-CD19.28ζ-, AAVS1_KO_-CD19.28ζ-, or 47_KO_-CD19.28ζ-CAR-T cells over days in culture. Mean ± SD of n = 3 T cell donors. (b) CD19.28ζ-, 47_OE_-CD19.28ζ-, AAVS1_KO_-CD19.28ζ-, or 47_KO_-CD19.28ζ-CAR-T cell viability over days in culture. Mean ± SD of n = 3 T cell donors. (c) Nalm6-GFP tumour killing by mock, CD19.28ζ-, 47_OE_-CD19.28ζ-, AAVS1_KO_-CD19.28ζ-, or 47_KO_-CD19.28ζ-CAR-T cells at a 1:1 E:T ratio measured via IncuCyte assay. Mean ± SD of n = 3 T cell donors, with each datapoint derived from the average of n = 3 triplicate wells per donor. Mock, CD19.28ζ, and 47_OE_-CD19.28ζ are shared conditions duplicated in (d). (d) Nalm6-GFP tumour killing by mock, CD19.28ζ-, or 47_OE_-CD19.28ζ-CAR T cells ± B6H12 at a 1:1 E:T ratio measured via IncuCyte assay. Mean ± SD of n = 3 T cell donors, with each datapoint derived from the average of n = 3 triplicate wells per donor. Conditions without B6H12 are shared conditions duplicated in (c). (e - f) IFN-γ (e) and IL-2 (f) secretion upon co-culture for 24 h with and without Nalm6 tumour cells at a 1:1 E:T ratio of mock, CD19.28ζ-, 47_OE_-CD19.28ζ-, AAVS1_KO_-CD19.28ζ- or 47_KO_-CD19.28ζ-CAR-T measured via ELISA. Mock, CD19.28ζ-, and 47_OE_-CD19.28ζ-CAR-T cells were also assessed in the presence of B6H12 and Nalm6. Mean ± SD of n = 3 T cell donors, with each datapoint derived from the average of n = 3 triplicate wells per donor. (g - l) Annexin V (g), CD69 (h), CD39 (i), TIM3 (j), LAG3 (k), and PD1 (l) expression upon co-culture for 24 h with and without Nalm6 tumour cells at a 1:1 E:T ratio of mock, CD19.28ζ-, 47_OE_-CD19.28ζ-, AAVS1_KO_-CD19.28ζ- or 47_KO_-CD19.28ζ-CAR-T measured via flow cytometry. Mock, CD19.28ζ-, and 47_OE_-CD19.28ζ-CAR-T cells were also assessed in the presence of B6H12 and Nalm6. Mean ± SD of n = 3 T cell donors. (m) CD47 and CAR (second from left) expression on T cells by flow cytometry after CD47 over-expression (47_OE_). Representative of n > 3 donors. (n) Images of Nalm6 BLI on day 45 after dosing with CD19.28ζ- or 47_OE_-CD19.28ζ-CAR-T cells in Nalm6 tumour-bearing mice. (o) CD19.28ζ- or 47_OE_-CD19.28ζ-CAR T treated Nalm6 tumour growth by BLI. Mean ± SEM of n = 5 mice/arm. (p) CD11b^+^/F4/80^+^ macrophages collected via peritoneal lavage by flow cytometry on day 13 after clodronate treatment. Mean ± SD of n = 7 (macrophage depleted) or n = 9 (macrophage non-depleted) mice. (q) Representative flow plots and gating strategy for detection of macrophages in samples collected following peritoneal lavage. (r) CD19.28ζ-nLuc-CAR T BLI before (day 7) and after (day 9) treatment with B6H12 of mice ± macrophage depletion (started on day 0). Mean ± SD of n = 4 (macrophage depleted, PBS treated) or n = 5 (all other groups) mice. [(a), (b), (c), (d), (e), (f), (g), (h), (i), (j), (k), (l), (o), (r)] Two-way analysis of variance (ANOVA) test with Tukey’s multiple comparison test. ns = not significant. [(p)] Unpaired two-tailed Student’s *t* test. Source Data

Given the essential role for CD47 expression for T cell persistence in vivo, we wondered whether overexpression of CD47 (47_OE_), which has been reported to prevent immune rejection by allogeneic cells^13^, could enhance CAR T cell persistence and efficacy in NSG mice, in which immune rejection does not occur due to profound immune suppression. Modulation of CD47 expression (either through knock out or overexpression) or addition of anti-CD47 did not alter CAR T cell function in vitro (Extended Data Fig. 3a–l). However, 47_OE_ CD19-28ζ CAR T cells mediated significantly better long-term antitumour efficacy and improved T cell persistence compared with the controls in vivo (Fig. 1j,k and Extended Data Fig. 3m–o). Together, these data demonstrate that the survival of adoptively transferred T cells requires CD47 expression and SIRPα engagement and that CD47 overexpression enhances CAR T cell persistence and efficacy, even in the absence of CD47 blocking agents and in the absence of immune rejection^13^.

On the basis of evidence that CD47 blockade enhances macrophage phagocytosis of tumour cells^7^, we examined whether macrophages mediated T cell depletion induced by anti-CD47 by depleting macrophages (Extended Data Fig. 3p,q) and then treating the mice with CD19-28ζ-nLuc CAR T cells with or without B6H12. The day after adoptive transfer but before B6H12 administration, BLI analysis revealed significantly higher CAR T cell numbers in macrophage-depleted mice, consistent with a model in which macrophages mediate T cell depletion even in the absence of CD47 blockade (Fig. 1l,m and Extended Data Fig. 3r). After B6H12 administration, we observed no loss of CAR T cell BLI signal in macrophage-depleted mice, but a significantly reduced CAR T cell BLI signal in mice with an intact macrophage compartment (Fig. 1l,m and Extended Data Fig. 3r). Together, these results identify macrophages as barriers to engraftment and antitumour efficacy of adoptively transferred T cells and demonstrate an essential requirement for adequate levels of CD47 on T cells to engage SIRPα, even in hosts that are incapable of recognizing allogeneic disparities. They further explain the futility of combining anti-CD47 with adoptive T cell therapy and implicate macrophage-mediated phagocytosis as an important regulator of T cell persistence in vivo.

## Human macrophages phagocytose T cells

**Fig. 2:**
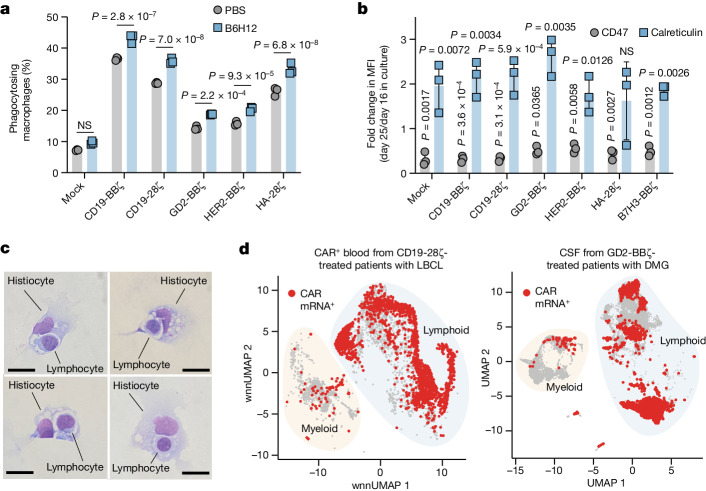
Macrophages phagocytose CAR T cells in vitro and in patients. **a**, Phagocytosis of co-cultured CFSE^+^ CAR T cells by primary human macrophages by flow cytometry. Data are mean ± s.d. of *n* = 3 triplicate wells. Reproducible across *n* = 4 macrophage donors. **b**, The fold change in CD47 and calreticulin expression on CAR T cells between days 25 and 16 of culture by flow cytometry. Data are mean ± s.d. of fold change of values (day 25/day 16) derived from *n* = 3 donors. MFI, mean fluorescence intensity. **c**, Microscopy images of Wright-Giemsa-stained histiocytes engulfing lymphocytes collected from the CSF of a patient with LBCL who was treated with CD19-28ζ CAR T cells. Representative of a sample collected from a single patient. Scale bars, 10 μm. **d**, scRNA-seq landscapes, with CAR mRNA shown in red. Left, *n* = 6,316 CAR^+^ cells sorted from blood of *n* = 9 axi-cel CD19-28ζ-treated patients with LBCL collected on day 7 after CAR T cell infusion. Right, *n* = 25,598 cells from the CSF of *n* = 4 GD2.BBζ-treated patients with DMG. Both sampled to 500 cells per patient sample. Statistical analysis was performed using unpaired two-tailed Student’s *t*-tests (**a** and **b**); for **b**, comparison is between expression values of the indicated group on day 16 versus day 25. Source Data

**Extended Data Fig. 4:**
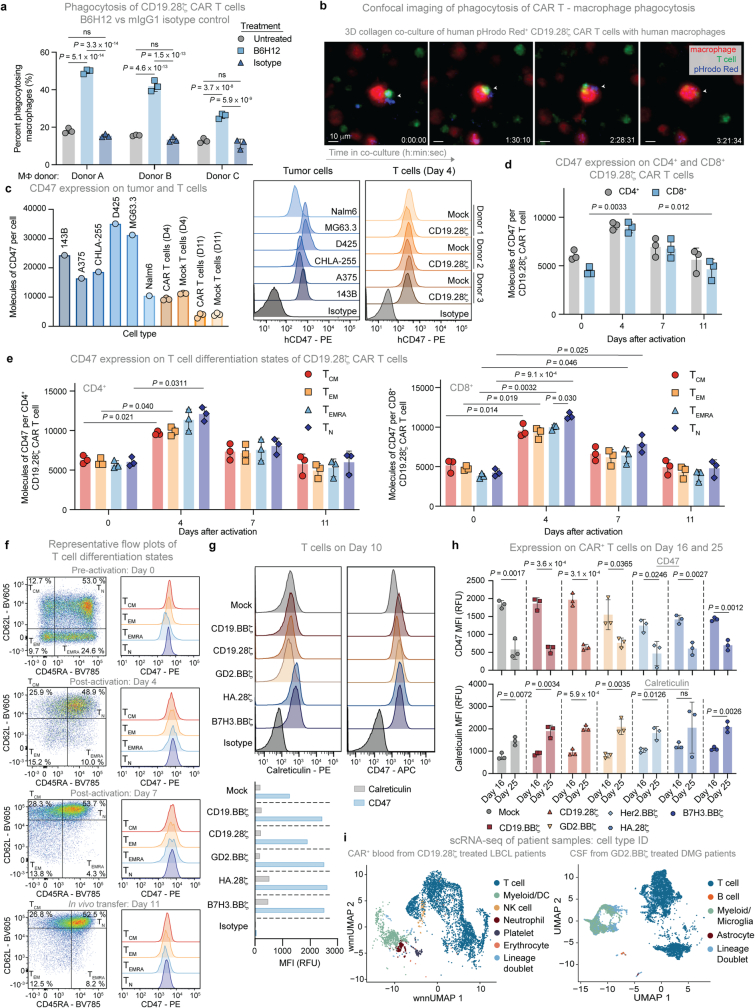
CD47 expression is uniform among CAR T cell subtypes and decreases over time in culture. (a) Phagocytosis of CFSE labelled CD19.28ζ-CAR-T cells from three donors by primary human macrophages from three donors by flow cytometry, following one hour of co-culture. CAR T cells were either untreated or treated with B6H12 or mIgG1 isotype control prior to addition of macrophages. Mean ± SD of n = 3 T cell donors, with each datapoint derived from the average of n = 3 triplicate wells per donor. (b) Representative confocal images of primary human CD19.28ζ-CAR-T cell phagocytosis by a primary human macrophage after treating with B6H12 in a three-dimensional collagen matrix. Images from left to right represent 0 min (initial image), 1 h 30 min, 2 h 28 min, and 3 h 21 min of T cell – macrophage co-incubation. CAR T cells (green) and macrophages (red) were labelled separately with lipophilic dyes. CAR T cells were also labelled with pH sensitive pHrodo Red dye (shown in blue) prior to B6H12 treatment. White arrows indicate a phagocytic event. See SI Video 1 for accompanying time-lapse video. Images are representative across n = 2 experiments. (c) CD47 expression on tumour cells, mock T cells, and CD19.28ζ-CAR-T cells by QuantiBrite quantitative flow cytometry. T cells were assessed on day 4 and day 11 of culture. Quantification of histograms shown on the left for each cell type. Representative of n = 1 tumour sample and n = 3 T cell donors and reproducible across n > 3 experiments. (d) CD47 expression on CD4^+^ and CD8^+^ CD19.28ζ-CAR-T cells by QuantiBrite quantitative flow cytometry. Cells were assessed by flow cytometry on day 0 (prior to activation), day 4 (after activation bead removal), day 7, and day 11 (time of typical transfer in vivo). Mean ± SD of n = 3 T cell donors. Only significant differences are shown. All other groups are not significantly different, comparing between the same subtype over time and between subtypes at the same timepoint. (e) CD47 expression on T_N_, T_CM_, T_EM_, and T_EMRA_ CD4^+^ (left) and CD8^+^ (right) CD19.28ζ-CAR-T cell subtypes on days 0, 4, 7, and 11 post-activation by QuantiBrite quantitative flow cytometry. Mean ± SD of n = 3 T cell donors. Only significant differences are shown. All other groups are not significantly different, comparing between the same subtype over time and between subtypes at the same timepoint. (f) Representative flow cytometry plots of CD19.28ζ-CAR-T cells pre- (day 0) and post-activation (days 4, 7, and 11). Left column: gating for T cell differentiation states, T naïve (CD45RA^+^/CD62L^+^ | T_N_), T central memory (CD45RA^−^/CD62L^+^ | T_CM_), T effector memory (CD45RA^−^/CD62L^−^ | T_EM_), and T effector memory re-expressing CD45RA (CD45RA^+^/CD62L^−^ | T_EMRA_). Right column: histograms of CD47 expression across T cell differentiation states. (g) Calreticulin (left) and CD47 (right) expression on CAR-T cells by flow cytometry on day 10 of culture. Quantification of histograms shown on the bottom for each type of CAR-T cell. Representative of n = 3 donors. (h) Quantification of CD47 (top) and calreticulin (bottom) expression on CAR^+^ T cells on day 16 and day 25 of culture by flow cytometry. Mean ± SD of n = 3 donors. (i) Single cell landscapes colour coded for cell type. Top: Weighted nearest neighbour (wnn) UMAP derived from *n* = 6,316 CAR^+^ cells sorted from the blood collected on day 7 after CAR T infusion of *n* = 9 axi-cel (CD19.28ζ) treated LBCL patients, sampled to 500 cells/patient sample. Bottom: UMAP derived from *n* = 25,598 cells from the CSF of *n* = 4 GD2.BBζ treated diffuse midline glioma (DMG) patients, sampled to 500 cells/patient sample. [(a), (d), (e)] Two-way analysis of variance (ANOVA) test with Tukey’s multiple comparison test. [(h)] Unpaired two-tailed Student’s *t* test. For all: ns = not significant. Source Data

We next investigated the potential for primary human macrophages to phagocytose primary human T cells in vitro. We observed macrophage phagocytosis of mock-transduced T cells at baseline; however, T cells transduced to express CARs were phagocytosed at significantly higher levels, which was further increased with B6H12 (Fig. 2a, Extended Data Fig. 4a,b and Supplementary Video 1). Macrophage phagocytosis is regulated by a balance of ‘eat me’ signals, such as calreticulin^7^, and ‘don’t eat me’ signals, such as CD47. Flow cytometry revealed that CAR T cells expressed fewer CD47 molecules than the tumour lines used in this study (Extended Data Fig. 4c), consistent with a model in which CAR T cells present limiting ‘don’t eat me’ signals to macrophages. CD47 expression was relatively uniform between CD4^+^ and CD8^+^ T cells and among T cell differentiation states; however, CD47 expression decreased over time in culture while calreticulin expression increased during the same period (Fig. 2b and Extended Data Fig. 4d–h), consistent with a model in which aged CAR T cells are more susceptible to phagocytosis.

During the course of these experiments, routine cytologic analysis of cerebrospinal fluid (CSF) collected from a patient treated with axicabtagene ciloleucel (axi-cel), a commercial CD19-28ζ CAR T cell therapy, revealed histiocytes engulfing lymphocytes (Fig. 2c), consistent with macrophage-mediated phagocytosis. To address this possibility more systematically, we analysed single-cell RNA-sequencing (scRNA-seq) data collected from two recent clinical studies of axi-cel^14^ for large B cell lymphoma (LBCL) and GD2-BBζ CAR T cells^15^ for diffuse midline glioma (DMG). Both datasets demonstrated CAR mRNA in myeloid cells, consistent with macrophage-mediated phagocytosis of CAR T cells in humans (Fig. 2d and Extended Data Fig. 4i). These data provide further evidence in support of a model in which myeloid cells phagocytose CAR T cells, and may therefore limit durable engraftment of adoptively transferred cells or the survival of activated T cells in clinical settings.

## Anti-CD47 can function as a safety switch

**Fig. 3:**
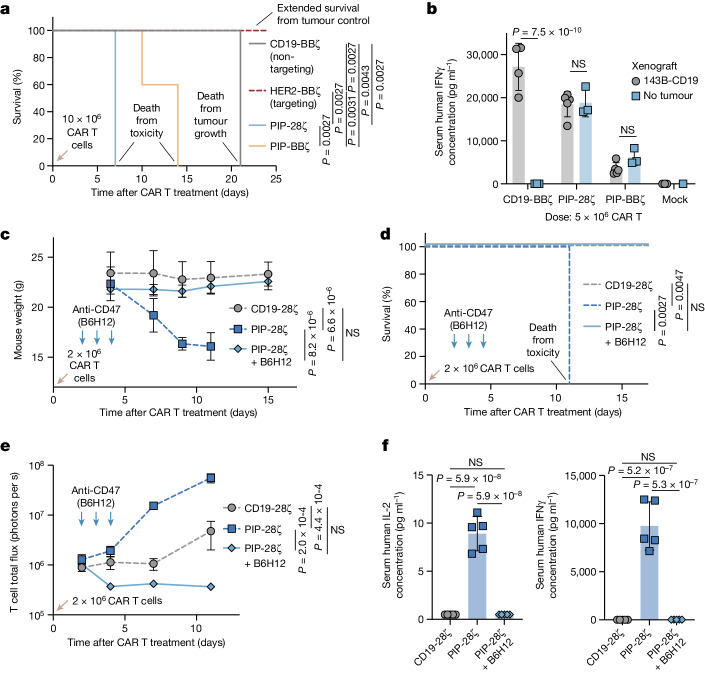
Anti-CD47 therapy can be used as a safety switch. **a**, The survival of 143B tumours after treatment with CD19-BBζ (non-targeting), HER2-BBζ (targeting), PIP-28ζ or PIP-BBζ CAR T cells. *n* = 5 mice per arm. **b**, Human IFNγ in the blood of mice with or without 143B-CD19 tumours, treated with CD19-BBζ, PIP-28ζ or PIP-BBζ CAR T cells on day 4, as determined using LEGENDPlex quantitative flow cytometry. Data are mean ± s.d. of *n* = 5 (PIP-28ζ or PIP-BBζ with 143B-CD19 tumour), *n* = 4 (CD19-BBζ with 143B-CD19 tumours), *n* = 3 (mock with 143B-CD19 tumour and CD19-BBζ, PIP-28ζ or PIP-BBζ without tumours) and *n* = 1 (mock without tumour) mice. **c**, Mouse weights after PIP-28ζ CAR T cell treatment with or without B6H12. Data are mean ± s.d. of *n* = 5 mice per arm. Representative of two independent experiments. **d**, The survival of mice treated with PIP-28ζ CAR T cells with or without B6H12. *n* = 5 mice per arm. Representative of two independent experiments. **e**, T cell BLI after treatment with PIP-28ζ CAR T cells with or without B6H12. Data are mean ± s.e.m. of *n* = 5 mice per arm. **f**, IL-2 (left) and IFNγ (right) in the blood of mice treated with PIP-28ζ CAR T cells with or without B6H12 on day 4, as determined using LEGENDPlex quantitative flow cytometry. Data are mean ± s.d. of *n* = 5 mice. Statistical analysis was performed using log-rank Mantel–Cox tests (**a** and **d**), unpaired two-tailed Student’s *t*-tests (**b**), two-way ANOVA (**c** and **e**) and one-way ANOVA with Tukey’s multiple-comparison test (**f**). Source Data

**Extended Data Fig. 5:**
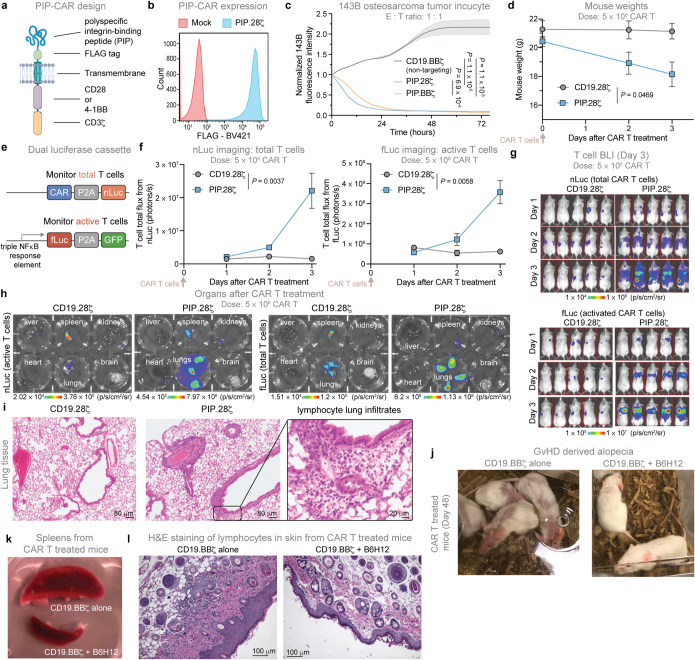
Anti-CD47 can limit toxicities from a pan-tumour integrin targeting PIP-CAR and GvHD. (a) Cartoon of the PIP-CAR design. The diagram was created using BioRender. (b) PIP.28ζ-CAR expression by flow cytometry. Representative of n = 3 donors. (c) 143B-GFP tumour killing by PIP.28ζ-, PIP.BBζ-, or non-tumour targeting CD19.BBζ-CAR T cells at a 1:1 E:T ratio measured via IncuCyte assay. Mean ± SD of n = 3 triplicate wells and reproducible in three independent experiments with different donors. (d) Weights of mice following treatment with CD19.28ζ- or PIP.28ζ-CAR-T. Mean ± SEM of n = 5 mice/arm. (e) Cartoon of the dual luciferase reporter design for tracking CAR-T activity in vivo. nLuc is linked to CAR expression, fLuc is linked to NF-κB activation. (f) T cell BLI of mice treated with CD19.28ζ- or PIP.28ζ-nLuc_NFκB-fLuc-CAR T cells. Left: nLuc (total T cells). Right: fLuc (active T cells). Mean ± SEM of n = 5 mice/arm. (g) Images of T cell BLI of mice treated as in (f), 3 days after infusion. Top: nLuc (total T cells). Bottom: fLuc (activated T cells). n = 5 mice/arm. (h) Representative images of BLI from organs extracted from mice treated as in (f), four days after CAR T administration. Left: nLuc (total T cells). Right: fLuc (active T cells). Representative of n = 5 mice/arm. (i) Representative hematoxylin and eosin (H&E) stained images of lung tissue collected from mice treated as in (f), four days after CAR T administration. Right panel is a higher magnification view of the boxed region in the middle panel. Images are representative of n = 3 mice/arm. (j – l) Representative images of (j) mice demonstrating GvHD derived alopecia (day 48 post-CAR T infusion), (k) spleens from mice (_collected_ on day 48 post-CAR T infusion), and (l) H&E staining of skin sections (collected on day 48 post-CAR T infusion) from mice treated with CD19.BBζ-CAR T ± B6H12. Representative of n = 5 mice/arm. [(c), (d), (f)] Two-way analysis of variance (ANOVA) test with Tukey’s multiple comparison test. Source Data

We reasoned that anti-CD47-mediated T cell depletion could be harnessed as an off-the-shelf safety switch to mitigate CAR T cell toxicity. To test this, we used a polyspecific integrin-binding peptide (PIP) CAR, which expresses a cystine-knot peptide binding domain that targets integrins expressed on a wide array of malignancies^16^ (Extended Data Fig. 5a,b). PIP CAR T cells mediated potent activity in vitro, but osteosarcoma-bearing mice that were treated with PIP CAR T cells demonstrated acute toxicity (Fig. 3a and Extended Data Fig. 5c). Even in non-tumour-bearing mice, treatment with PIP-28ζ CAR T cells quickly resulted in toxicity (Fig. 3b and Extended Data Fig. 5d–i). By contrast, PIP-28ζ CAR T cell recipients treated with B6H12 did not demonstrate any overt toxicity or weight loss, including no detectable human cytokines in the blood (Fig. 3c–f). Prolonged CAR T cell persistence can lead to graft versus host disease (GvHD) in xenograft models. To determine whether anti-CD47 treatment could durably prevent GvHD, we monitored Nalm6-bearing mice treated with CD19-BBζ CAR T cells with or without B6H12 for the development of GvHD after successful tumour clearance. After 48 days, we observed GvHD in mice treated with CD19-BBζ CAR T cells, evidenced by alopecia and weight loss, whereas mice that were treated with CAR T cells plus B6H12 did not develop GvHD (Extended Data Fig. 5j–l). These data suggest that CD47 blockade could hold promise as an off-the-shelf safety switch to eliminate CAR-T-cell-associated toxicities, as evidenced by rescue in a stringent acute toxicity model as well as a model of chronic CAR T cell toxicity.

## Engineered CD47 does not bind to anti-CD47

**Fig. 4:**
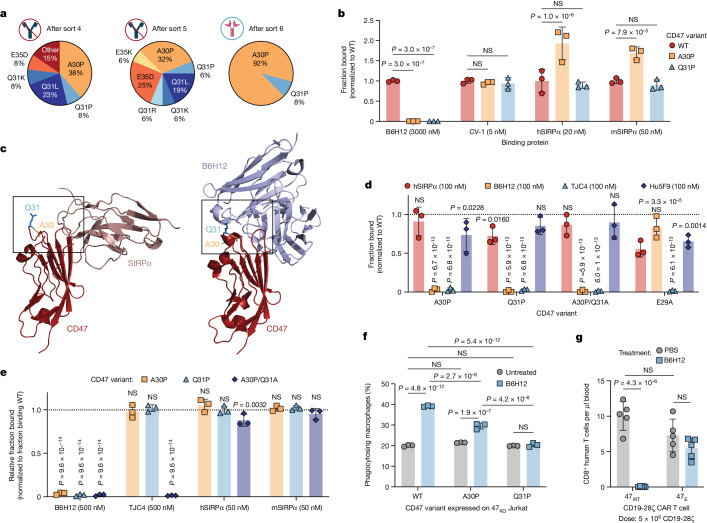
An engineered variant of CD47 retains binding to SIRPα, but no longer binds to anti-CD47 antibodies. **a**, Consensus mutations identified in yeast sequenced after sorts 4, 5 and 6. Frequencies of identified mutations out of *n* = 13, 16 and 12 sequenced clones for sorts 4, 5 and 6, respectively. **b**, Normalized binding of B6H12, CV-1, human SIRPα and mouse SIRPα to yeast-displayed CD47 WT, CD47(A30P) and CD47(Q31P). **c**, Crystal structures of CD47 (red) binding to SIRPα (dark pink, left) and B6H12 (light blue, right); residues Ala30 (gold) and Gln31 (blue) are indicated by boxes. **d**, Normalized binding of human SIRPα, B6H12, TJC4 and Hu5F9 to yeast-displayed CD47 WT, CD47(A30P), CD47(Q31P), CD47(A30P/Q31A) and CD47(E29A). **e**, Normalized binding of B6H12, TJC4, human SIRPα and mouse SIRPα to full-length CD47 WT, CD47(A30P) and CD47(Q31P) expressed on primary human T cells. Data are mean ± s.d. of *n* = 3 donors, normalized to the fraction binding to CD47 WT. **f**, Phagocytosis of Jurkat cells with endogenous *CD47* KO expressing CD47 WT, CD47(A30P) or CD47(Q31P), by primary human macrophages. Data are mean ± s.d. of triplicate wells (*n* = 3). Reproducible across *n* = 4 macrophage donors. **g**, CD8^+^ T cells in the blood on day 6 after treatment with 47_E_ CD19-28ζ CAR T cells with or without B6H12. Data are mean ± s.d. of *n* = 5 mice. For **b** and **d**, data are mean ± s.d. of *n* = 3 individual yeast clones, normalized to MFI from binding to CD47 WT. Statistical analysis was performed using two-way ANOVA with Tukey’s multiple-comparison test (**b** and **d**–**g**). For **d** and **e**, the comparisons are between the indicated groups and binding to cells expressing CD47 WT. Source Data

**Extended Data Fig. 6:**
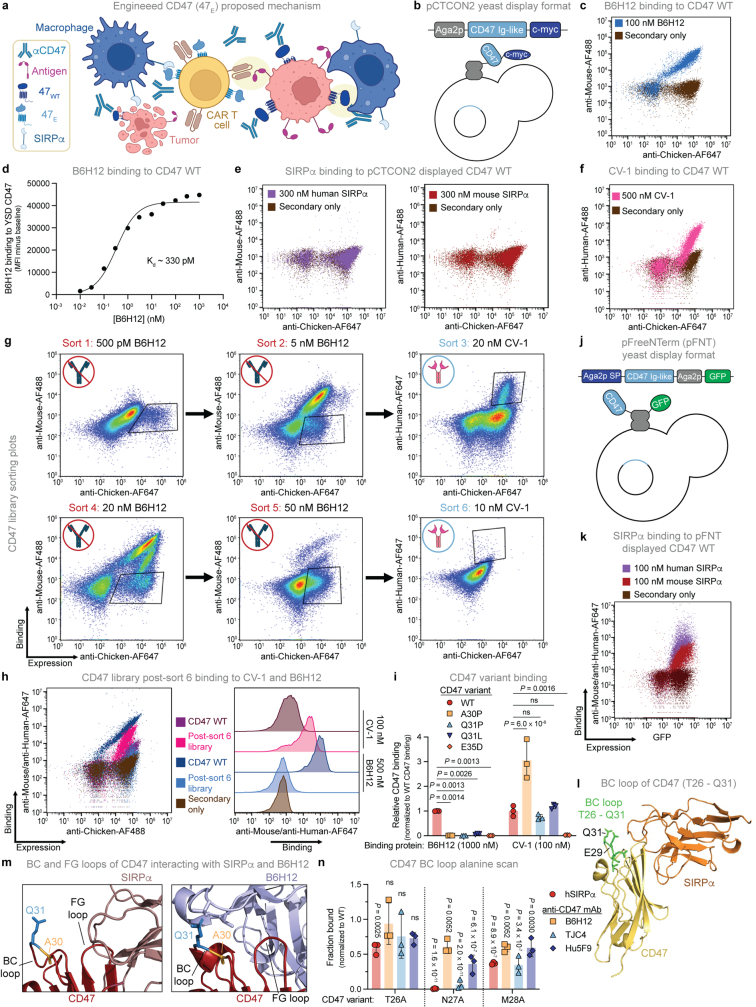
Engineered variants of CD47 retain SIRPα binding and demonstrate a loss of binding to some, but not all, anti-CD47 antibodies. (a) Engineered CD47 (47_E_) mechanism: αCD47 antibodies bind tumour cells, but not 47_E_-T cells, triggering tumour-specific phagocytosis. The diagram was created using BioRender. (b) Cartoon of yeast displayed CD47 Ig-like domain using the pCTCON2 vector. CD47 is displayed as an N-terminal fusion. (c) Binding of 100 nM B6H12 to yeast displayed CD47 in pCTCON2 by flow cytometry. Representative of n = 3 independent experiments. (d) Binding curve of B6H12 to yeast displayed CD47 in pCTCON2, measured over multiple concentrations by flow cytometry. MFI of n = 1 experiment. (e) Binding of 300 nM human (top) and mouse (bottom) SIRPα to yeast displayed CD47 in pCTCON2 by flow cytometry. Data are representative of n = 3 independent experiments. (f) Binding of 500 nM CV-1 to yeast displayed CD47 in pCTCON2 by flow cytometry. Data are representative of n = 3 independent experiments. (g) Flow cytometry sorting plots of all six sorts of the CD47 library, indicating negative sorts to B6H12 and positive sorts to CV-1. Collected population indicated by the black box in each plot. (h) Binding of 500 nM B6H12 or 100 nM CV-1 to the yeast displayed CD47 library population collected after sort 6 or yeast displayed WT CD47. Data are representative of n = 2 independent experiments. (i) Binding of 1 μM B6H12 or 100 nM CV-1 to CD47 variants displayed on yeast using the pCTCON2 vector. Mean ± SD of n = 3 individual yeast clones, normalized to MFI from binding to 47_WT_. (j) Cartoon of yeast-displayed CD47 Ig-like domain using the pFreeNTerm (pFNT) vector. CD47 is displayed as a C-terminal fusion, along with GFP to monitor protein expression. (k) Binding of 100 nM human and mouse SIRPα to yeast displayed CD47 in pFNT by flow cytometry. Representative of n = 3 independent experiments. (l) Crystal structure of CD47 (yellow) binding SIRPα (orange) [PDB: 2JJS], identifying the CD47 BC loop (green), containing CD47 residues T26 – Q31. (m) Crystal structures of CD47 (red) binding SIRPα (dark pink, left) [PDB: 2JJS] and B6H12 (light blue, right) [PDB: 5TZU], identifying residues A30 (gold) and Q31 (blue), and the BC and FG loops of CD47. Structures are enlargements of the boxed regions in the full structures shown in Fig. 4c. (n) Binding of 100 nM B6H12, TJC4, Hu5F9, and hSIRPα to yeast displayed 47_T26A_, 47_N27A_, and 47_M28A_ variants. Mean ± SD of n = 3 individual yeast clones, normalized to MFI from binding to 47_WT_. [(i), (n)] Two-way analysis of variance (ANOVA) test with Tukey’s multiple comparison test. ns = not significant. Comparison is between indicated group and binding to CD47 WT expressing cells. Source Data

To induce selective tumour phagocytosis through CD47 blockade while protecting T cells from phagocytosis, we sought to engineer a CD47 variant that ablates anti-CD47 binding but retains SIRPα interaction (Extended Data Fig. 6a). We first displayed the CD47 Ig-like domain on the surface of yeast and detected strong binding to B6H12, but not SIRPα (Extended Data Fig. 6b–e), due to the lack of a free N terminus on CD47^17^. We therefore used the engineered SIRPα variant CV-1^9^ as a proxy for SIRPα binding and subjected a library of yeast-displayed CD47 mutant variants to six successive sorts using fluorescence-activated cell sorting (FACS), alternating between negative sorts against B6H12 and positive sorts towards CV-1 (Extended Data Fig. 6f–h). All of the variants in the final sort contained a single A30P (CD47(A30P)) or Q31P (CD47(Q31P)) point mutation (Fig. 4a), which we confirmed, when displayed as individual CD47 variants on yeast with a free CD47 N terminus^17^, manifested no binding to B6H12 but retained similar or enhanced binding to CV-1 and SIRPα (Fig. 4b and Extended Data Fig. 6i–k). These results are consistent with structural understanding of CD47–SIRPα interactions, whereby SIRPα predominantly contacts CD47 through the CD47 FG loop and N-terminus^18^, forming more minor contacts with the CD47 BC loop, which encompasses Thr26–Gln31^18^ (Fig. 4c and Extended Data Fig. 6l,m). As the CD47 BC loop lies near the critical CD47 FG loop, it can serve as an anchoring point for anti-CD47 blocking monoclonal antibodies like B6H12^19^, with the Gln31 residue appearing to be particularly important for antibody binding^19^ (Fig. 4c and Extended Data Fig. 6m).

**Extended Data Fig. 7:**
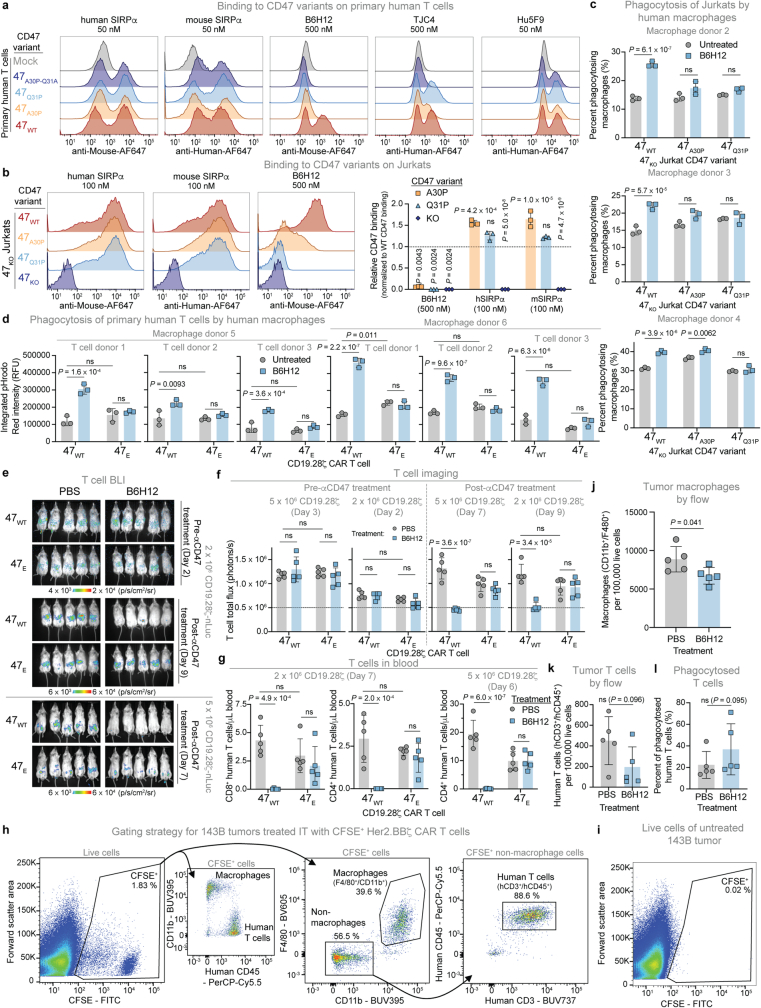
Expression of 47_E_ on T cells mitigates B6H12 induced phagocytosis due to lack of antibody binding in vitro and in vivo. (a) Representative flow cytometry histograms of 50 nM hSIRPα, 50 nM mSIRPα, 500 nM B6H12, 500 nM TJC4, and 50 nM Hu5F9 binding to 47_WT_, 47_A30P_, 47_Q31P_, and 47_A30P-Q31A_ over-expressed on primary human T cells. Data are representative of n = 3 different T cell donors in independent experiments. (b) Left: Representative flow cytometry histograms of 100 nM hSIRPα, 100 nM mSIRPα, and 500 nM B6H12 binding to 47_WT_, 47_A30P_, and 47_Q31P_ expressed on Jurkats with endogenous 47_KO_. Right: Binding of B6H12, hSIRPα, and mSIRPα to full-length 47_WT_, 47_A30P_, and 47_Q31P_ expressed on Jurkat cells with endogenous 47_KO_. Mean ± SD of n = 3 independent experiments, normalized to fraction binding to 47_WT_. (c) Phagocytosis by primary human macrophages from multiple donors of CFSE labelled Jurkats with endogenous 47_KO_, expressing 47_WT_, 47_A30P_, or 47_Q31P_ variants after one hour of co-culture ± B6H12. Mean ± SD of n = 3 triplicate wells. (d) Phagocytosis by primary human macrophages from two donors of pHrodo Red labelled primary human CD19.28ζ-CAR-T cells from three donors with endogenous 47_KO_, expressing 47_WT_ or 47_E_ (47_Q31P_) after three hours of co-culture ± B6H12. Mean ± SD of n = 3 triplicate wells. (e) Images of T cell BLI following treatment with 47_WT_- or 47_E_-CD19.28ζ-nLuc-CAR-T cells ± B6H12 in non-tumour bearing mice. (f) T cell BLI before and after αCD47 treatment, of mice treated as in (e). Mean ± SD of n = 5 mice. (g) hCD8^+^ and hCD4^+^ T cells in the blood on day 6 by flow cytometry of mice treated as in (e). Mean ± SD of n = 5 mice. (h) Representative flow plots and gating strategy for detection of CFSE^+^ macrophages and T cells in dissociated 143B tumour samples in the in vivo phagocytosis model treated with Her2.BBζ-CAR T cells ± B6H12. Representative of n = 5 mice/arm. (i) Representative flow plot and gating strategy for detection of CFSE^+^ cells of a dissociated 143B tumour from a mouse that did not receive CAR T cells. (j) mF4/80^+^/CD11b^+^ macrophages identified by flow cytometry of dissociated tumours treated as in (h). Mean ± SD of n = 5 mice. (k) hCD3^+^/hCD45^+^ human T cells identified by flow cytometry of dissociated tumours treated as in (h). Mean ± SD of n = 5 mice. (l) Percentage of phagocytosed T cells identified in each dissociated tumour, treated as in (h), calculated as the number of CFSE^+^ macrophages per 100,000 lives cells per sample divided by the total number of CFSE^+^ macrophages and T cells per 100,000 live cells per sample, identified by flow cytometry. Mean ± SD of n = 5 mice. [(b), (c), (d), (f), (g)] Two-way analysis of variance (ANOVA) test with Tukey’s multiple comparison test. ns = not significant. (b) comparison is between indicated group and binding to 47_WT_ expressing cells. [(j), (k)] Unpaired two-tailed Student’s *t* test. [(l)] Two-tailed Mann-Whitney Test. Source Data

To determine whether the CD47 variants bound to other CD47 blocking monoclonal antibodies, we analysed their binding to TJC4 (lemzoparlimab) and Hu5F9 (magrolimab) in a yeast display assay. An alanine scan of the entire BC loop (Thr26–Gln31) revealed that most mutations allowed for some SIRPα binding, with mutations to Ala30 or Gln31 manifesting the most minimal impact (Fig. 4d and Extended Data Fig. 6n). Hu5F9, which has a binding footprint that largely overlaps with SIRPα^8^, demonstrated minimal loss of binding to any of the BC loop variants. However, TJC4, which structurally binds to CD47 similarly to B6H12^20^, no longer bound to CD47(A30P), CD47(Q31P) and CD47(A30P/Q31A) nor to CD47(E29A), an additional variant that did not affect B6H12 binding (Fig. 4d). We next profiled the binding of SIRPα, B6H12 and TJC4 to full-length WT CD47, CD47(A30P), CD47(Q31P) and CD47(A30P/Q31A) expressed on primary human T cells. Binding of human and mouse SIRPα was largely unaffected by any of the three variants and we detected no B6H12 binding to any of the three variants, while TJC4 binding was completely ablated by the CD47(A30P/Q31A) double mutant (Fig. 4e and Extended Data Fig. 7a). These data demonstrate that CD47 mutations in the BC loop, and Ala30 and Gln31 specifically, generate proteins that retain SIRPα binding but are exempt from binding to multiple anti-CD47 monoclonal antibodies, providing a proof of concept for the ability to engineer ‘don’t eat me’ signalling CD47 variants that will not be blocked by anti-CD47 monoclonal antibodies, which we predicted would drive tumour-specific phagocytosis while sparing T cells in the TME.

## 47_E_ prevents anti-CD47-mediated phagocytosis

We next measured phagocytosis of 47_KO_ Jurkat cells engineered to express either CD47 WT, CD47(A30P) or CD47(Q31P) by human donor macrophages. CD47 variants expressed on Jurkat cells demonstrated similar binding properties to anti-CD47 monoclonal antibodies and SIRPα as observed on primary T cells, with CD47(Q31P) leading to the greatest loss of B6H12 binding (Extended Data Fig. 7b). Across multiple donors, macrophages mediated significantly reduced phagocytosis after B6H12 incubation with either variant, but CD47(A30P) provided less protection compared with CD47(Q31P), which completely prevented additional phagocytosis after incubation with B6H12 (Fig. 4f and Extended Data Fig. 7c). On the basis of this promising profile, we chose to move forward with CD47(Q31P), hereafter 47_E_ (engineered CD47) for further study.

To study the effects of 47_E_ in human T cells, we knocked out endogenous *CD47*, then retrovirally introduced CAR, TCR, and/or 47_E_ or CD47 WT (47_WT_), then measured phagocytosis in vitro and in vivo with or without B6H12. Across multiple T cell and macrophage donors, we observed that B6H12 treatment did not enhance phagocytosis of 47_E_ CD19-28ζ CAR T cells in vitro, in contrast to control 47_WT_ CD19-28ζ CAR T cells (Extended Data Fig. 7d). Similarly, while 47_WT_ or 47_E_ CD19-28ζ-nLuc CAR T cells administered to non-tumour-bearing mice manifested similar BLI levels before B6H12 administration, B6H12 depleted 47_WT_ CAR T cells but did not affect the levels of 47_E_ CAR T cell (Fig. 4g and Extended Data Fig. 7e–g). These data demonstrate that 47_E_ functions as a ‘don’t eat me’ signal in vitro and in vivo but does not render T cells susceptible to phagocytosis after B6H12-mediated CD47 blockade.

## CAR T cells recruit macrophages into tumours

**Fig. 5:**
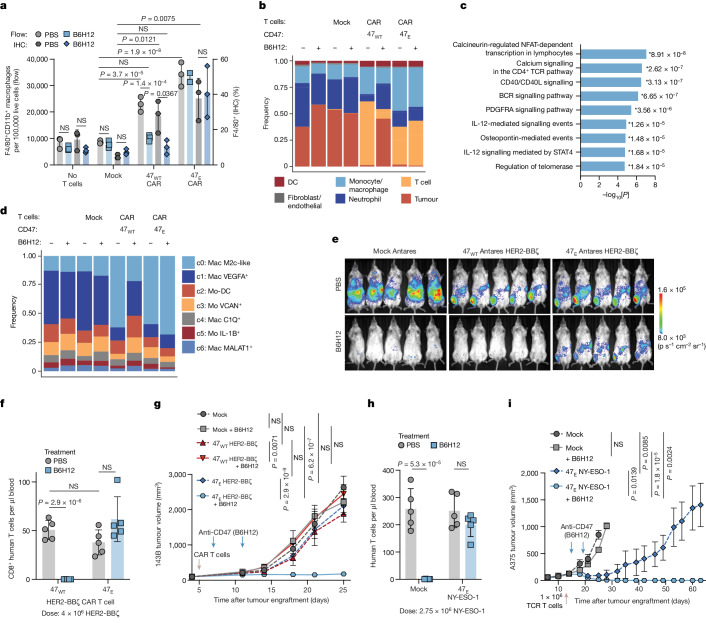
47_E_ T cell therapy plus anti-CD47 treatment leads to enhanced antitumour efficacy through recruitment of distinct macrophage populations. **a**, Macrophages identified by flow cytometry (flow; left axis) and immunohistochemistry (IHC; right axis) of excised 143B osteosarcoma tumours treated with no T cells, mock, 47_WT_ or 47_E_ HER2-BBζ CAR T cells with or without B6H12. Data are the mean of *n* = 2 (flow: 47_E _CAR + B6H12) or mean ± s.d. of *n* = 3 (all others) mice. **b**, The composition of cells identified using scRNA-seq from tumours treated as in **a**. *n* = 53,062 cells from 8 experimental conditions. DC, dendritic cells. **c**, Enrichr pathway analysis of the top 100 upregulated genes in CAR T cells from tumours treated with 47_E_ CAR T cells + B6H12 versus 47_WT_ CAR T cells + B6H12. The results show the *P* value (two-sided Fisher’s exact test) for each pathway. **d**, The composition of macrophage clusters (c0–c6) after subsetting and reclustering, coloured by cluster, across the experimental conditions described in **a**. *n* = 13,082 cells from 8 experimental conditions. Mac, macrophage; Mo-DC, monocyte-derived dendritic cell; Mo, monocyte. **e**, T cell BLI in 143B-tumour-bearing mice treated with 47_WT_ or 47_E_ Antares HER2-BBζ CAR T cells with or without B6H12 on day 13. Tumours were engrafted in the right leg. **f**, T cells in the blood of 143B-tumour-bearing mice, treated as described in **e**, on day 14. Data are mean ± s.d. of *n* = 5 mice per arm. **g**, 143B tumour growth after treatment as described in **e**. Data are mean ± s.e.m. of *n* = 5 mice per arm. **h**, T cells in the blood of A375-tumour-bearing mice, treated with 47_E_ NY-ESO-1 TCR T cells with or without B6H12, on day 15. Data are mean ± s.d. of *n* = 5 mice per arm. **i**, A375 melanoma tumour growth treated as in **h**. Data are mean ± s.e.m. of *n* = 5 mice per arm. Statistical analysis was performed using two-way ANOVA with Tukey’s multiple-comparison test (**a**, **b**, **f**, **g** and **i**) and unpaired two-tailed Student’s *t*-tests (**h**). Source Data

**Extended Data Fig. 8:**
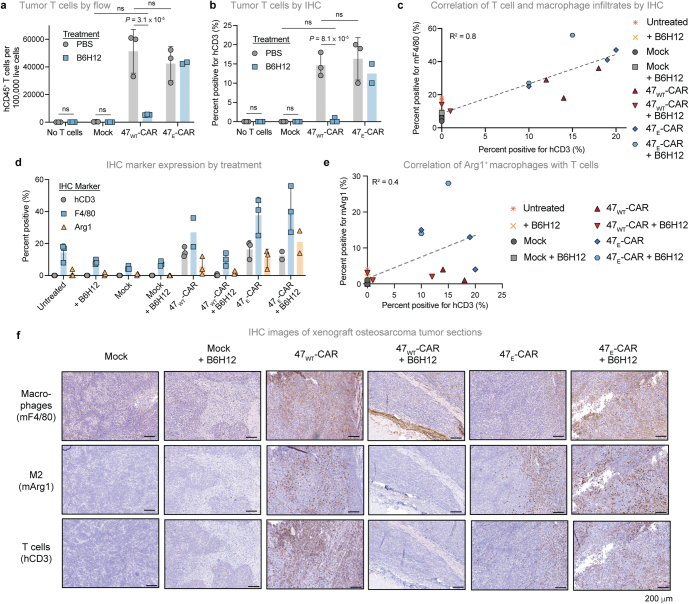
CAR-T treatment increases tumour infiltration of macrophages. (a) hCD45^+^ T cells identified by flow cytometry of dissociated 143B tumours in the 143B correlative model, treated with no T cells, mock, 47_WT_- or 47_E_-Her2.BBζ-CAR-T cells ± B6H12. Mean of n = 2 (47_E_-CAR + B6H12) or mean ± SD n = 3 (all other samples) mice. (b) Percent positivity for hCD3 T cells identified by IHC from tumour sections treated as in (a). Mean of n = 2 (47_E_-CAR + B6H12) or mean ± SD n = 3 (all other samples) mice. (c) Correlation of hCD3 and mF4/80 staining in IHC sections of tumours treated as in (a). Data points are representative of individual tumours, coloured by treatment group (n = 23). R^2^ calculated by simple linear regression. (d) Percent positivity for hCD3 (T cells), mArg1 (M2 macrophages), and mF4/80 (total macrophages) cells identified by IHC from tumour sections treated as in (a). Mean of n = 2 (47_WT_-CAR: F4/80; 47_E_-CAR + B6H12: CD3; 47_E_-CAR + B6H12: Arg1) or mean ± SD of n = 3 (all others) mice. (e) Correlation of hCD3 and mArg1 percent positivity by IHC of tumour sections treated as in (a). Datapoints are representative of individual tumours, coloured by treatment group (n = 23). R^2^ calculated by simple linear regression. (f) Representative images of IHC tumour sections stained for mF4/80, mArg1, and hCD3, treated as in (a). Representative of tumour sections collected from n = 3 mice/arm. [(a), (b)] Two-way ANOVA test with Tukey’s multiple comparison test. ns = not significant. Source Data

**Extended Data Fig. 9:**
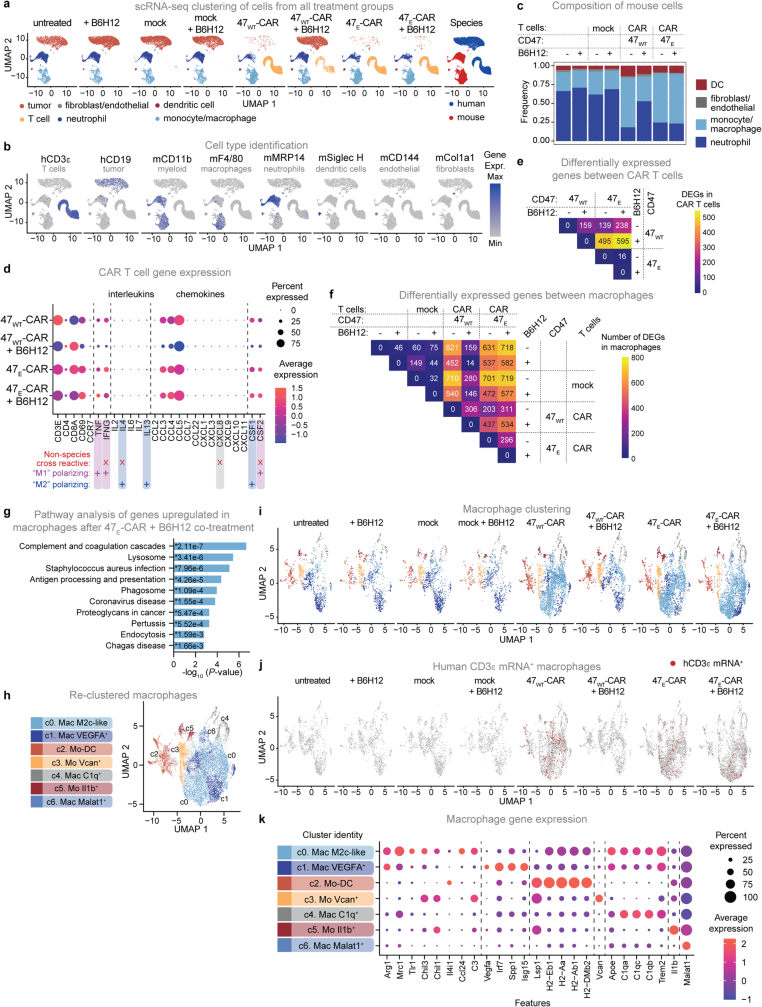
CAR-T treatment increases tumour infiltration of distinct macrophage populations. (a and b) scRNA-seq profile of dissociated tumour and infiltrating immune cells in the 143B correlative model, treated with no T cells, mock, 47_WT_- or 47_E_-Her2.BBζ-CAR-T cells ± B6H12. Dots represent individual cells. *n* = 53,062 cells from 8 experimental conditions with three mice per treatment group, coloured by (a) cell type (left eight plots; UMAPs represent distinct treatment conditions), species (far right), or (b) gene expression level. (c) Composition of murine cells identified via scRNA-seq from 143B tumours treated as in (a). Data are derived from n = 53,062 cells pooled from 8 experimental conditions with three mice per treatment group. Cell types assigned using SingleR automated cell type recognition. (d) Dot Plot depicting scRNA-seq expression of selected T cell subset markers, cytokines, and chemokines. n = 11,044 human tumour infiltrating T cells from treatments described in (a). (e) Comparison of differentially expressed genes between CAR-T cells of different treatment groups described in (a). Statistical significance was determined with Seurat; *P adj <0.05. (f) Comparison of differentially expressed genes between macrophages of different treatment groups described in (a). Statistical significance was determined with Seurat; *P adj <0.05. (g) Enrichr pathway analysis of the top 100 upregulated genes in tumour infiltrating macrophages [macrophage/monocyte population in (a)] in 47_E_-CAR + B6H12 treated tumours compared with untreated controls. Results depict the *P* value (two-sided Fisher’s exact test) of each pathway. (h) UMAP of the identified macrophage/monocyte population in (a), subsetted and re-clustered, coloured by cluster (cluster identities on the left). Dots represent individual cells. *n* = 13,082 cells from 8 experimental conditions. (i) UMAPs of the identified macrophage/monocyte population, subsetted and re-clustered, coloured by cluster (cluster identities in (h)). UMAPs represent distinct treatment conditions. Dots represent individual cells. *n* = 13,082 cells from 8 experimental conditions described in (a). (j) UMAP of the re-clustered macrophage/monocyte population from (m), coloured for red if hCD3ε mRNA expression > 0. Dots represent individual cells. *n* = 13,082 cells from 8 experimental conditions, with n = 350 total cells identified as hCD3ε mRNA^+^. UMAPs represent distinct treatment conditions described in (a). (k) Dot plot depicting scRNA-seq expression of selected cluster-defining genes within the macrophage populations identified in (h). *P* adj. <0.0001 for each selected gene. Source Data

We next quantified macrophage-mediated T cell phagocytosis in the stringent 143B model (Fig. 1a). CFSE-labelled HER2-BBζ CAR T cells were injected intratumourally into established orthotopic tumours, with or without B6H12 treatment. Tumour samples revealed robust macrophage infiltration, and trends of decreased CAR T cells and increased CFSE^+^ macrophages after B6H12 treatment, consistent with phagocytosis of CAR T cells in vivo (Extended Data Fig. 7h–l). We next treated 143B-tumour-bearing mice with no T cells, mock, or 47_WT_ or 47_E_ HER2-BBζ CAR T cells with or without B6H12 and observed significant increases in mouse macrophages in CAR T cell recipients compared with in the mock or untreated animals (Fig. 5a). B6H12 did not affect the TME macrophage levels in the recipients of 47_E_ CAR T cells, but significantly reduced the macrophage levels in the recipients of 47_WT_ CAR T cells coincident with CAR T cell depletion (Fig. 5a and Extended Data Fig. 8a,b). Confirmed through scRNA-seq analysis, CAR T cell and macrophage tumour infiltration were highly correlated, consistent with a model in which CAR T cells recruit macrophages into the tumour, and macrophage persistence is dependent on CAR T cell persistence in the tumour (Fig. 5b and Extended Data Figs. 8c–f and 9a–c).

scRNA-seq profiling of 47_WT_ and 47_E_CAR T cell recipients demonstrated robust T cell expression of *TNF*, *IFNG*, *CCL3*, *CCL4*, *CCL5*, *CSF1 *(also known as M-SCF) and *CSF2 *(also known as GM-CSF) (Extended Data Fig. 9d), which collectively attract and activate monocytes and macrophages and have been implicated in T-cell-mediated recruitment of macrophages into tumours^21^. Gene expression in recipients of 47_E_ CAR T cells was essentially unchanged with or without B6H12 therapy, whereas T cells in the TME of B6H12 recipients co-treated with 47_E_ CAR T cells showed 595 differentially expressed genes (DEGs) compared with those co-treated with 47_WT_ CAR T cells (Extended Data Fig. 9e and Supplementary Table 1), including increased proinflammatory genes sets such as IL-12, CD40/CD40L and NF-κB signalling (Fig. 5c). These data provide evidence for substantial cross-talk between myeloid cells and T cells within the 47_E_ CAR T cell TME, which is lacking in the TME of 47_WT_ CAR T cell recipients after B6H12-induced T cell depletion. DEG analyses within the major macrophage clusters across treatments showed that treatment with 47_WT_ CAR T cells alone induced 621 DEGs in macrophages compared with the untreated condition, and this effect was magnified after 47_E_ CAR T cell co-treatment with B6H12, with 718 DEGs (Extended Data Fig. 9f and Supplementary Table 2). Notably, the effect of B6H12 therapy on macrophage gene expression when administered as a single agent was minimal (46 DEGs), while B6H12 co-administration with 47_WT_ CAR T cells substantially reduced macrophage DEGs, probably due to CAR T cell depletion (Extended Data Fig. 9f). By contrast, pathway analysis of genes upregulated by 47_E_ CAR T cells plus B6H12 highlighted macrophage activation indicated by enrichment of lysosome, complement, antigen presentation and phagosome pathways (Extended Data Fig. 9g).

To further characterize changes in the macrophage compartment induced by 47_E_ CAR T cells, we reclustered the macrophage/monocyte cluster (Extended Data Fig. 9h,i). As observed in clinical data (Fig. 2d), we identified numerous macrophages that contained human *CD3E *mRNA within multiple CAR-T-cell-treated macrophage subclusters (Extended Data Fig. 9j), consistent with macrophage-mediated phagocytosis of CAR T cells. We also observed expansion of macrophage cluster c0, which was enriched after CAR T cell treatment and further expanded after treatment with 47_E_ CAR T plus B6H12, but substantially reduced after treatment with 47_WT_ CAR T cells plus B6H12 (Fig. 5d), suggesting that these macrophages are dependent on CAR T cell accumulation within the TME. Key DEGs in the expanded cluster, such as *Arg1*, *Mrc1*, *Chil3* and *Tlr1*, were associated with M2c-like macrophages^3^ (Extended Data Fig. 9k and Supplementary Table 3). While M2 macrophages are generally understood to be protumorigenic, they have also been demonstrated to manifest strong phagocytic potential, especially those in the M2c subclass^3^.

Together, these results demonstrate a feedforward loop in which CAR T cells drive the recruitment and activation of macrophages within the TME and, simultaneously, macrophages enhance immune-activating pathways in 47_E_ CAR T cells in the TME. These effects did not occur within the 47_WT_ CAR T cell TME, in which CD47-blockade-mediated CAR T cell depletion abrogates the cycle. The data further demonstrate robust cross-talk between CAR T cells and macrophages in the TME, resulting in the induction of several proinflammatory gene expression programs that are predicted to enhance antitumour effects.

## Anti-CD47 enhances efficacy of 47_E_ CAR/TCR T cells

**Extended Data Fig. 10:**
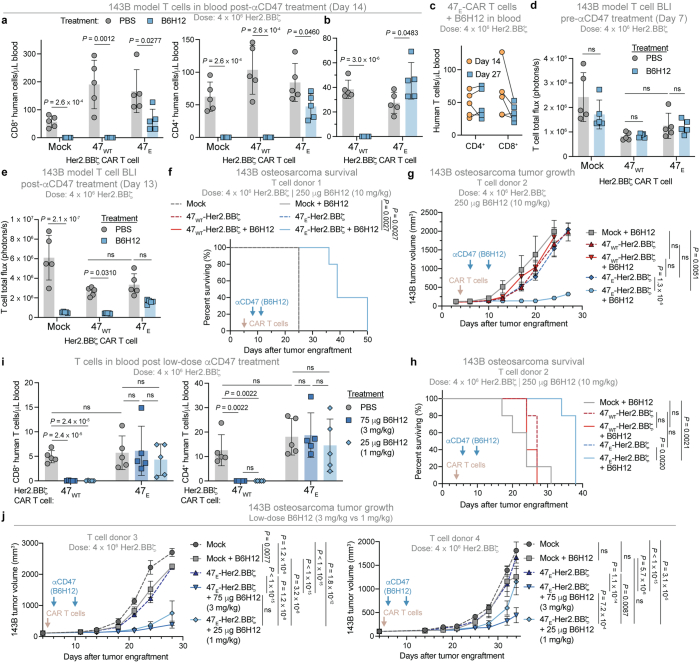
Pairing of 47_E_-CAR T and anti-CD47 therapy enhances tumour control in an aggressive osteosarcoma model, even at low doses. (a – b) hCD8^+^ (left, a) and hCD4^+^ (right, a & b) T cells derived from two independent donors (represented by panels a & b) in the blood on day 14 by flow cytometry after B6H12 treatment in 143B tumour bearing mice, treated with 47_WT_- or 47_E_-Her2.BBζ-Antares-CAR-T ± B6H12. Mean ± SD of n = 5 mice. (c) hCD4^+^ and hCD8^+^ T cells in the blood on day 14 and day 27 of tumour growth in 143B tumour bearing mice, treated with 47_E_-Her2.BBζ-Antares-CAR-T + B6H12. Datapoints represent individual mice, with values from the same mouse connected by lines between time points. (d) T cell BLI prior to B6H12 treatment in mice treated as in (a). Mean ± SD of n = 5 mice. (e) T cell BLI after B6H12 treatment on day 13 in mice treated as in (a). Mean ± SD of n = 5 mice. (f) 47_WT_- or 47_E_-Her2.BBζ-Antares-CAR-T ± B6H12 treated 143B tumour survival. n = 5 mice/arm. (g and h) 47_WT_- or 47_E_-Her2.BBζ-Antares-CAR-T ± B6H12 treated 143B tumour (g) growth and (h) survival, using T cells derived from a different donor than (f). Mean ± SEM (g) or representative (h) of n = 5 mice. (i) hCD8^+^ (left) and hCD4^+^ (right) T cells derived from in the blood on day 12 by flow cytometry after treatment with 47_WT_- or 47_E_-Her2.BBζ-CAR-T ± low-doses of 75 µg or 25 µg B6H12 treatment in 143B tumour bearing mice. Mean ± SD of n = 5 mice. (j) 47_E_-Her2.BBζ-CAR-T ± low-dose B6H12 (75 µg or 25 µg) treated 143B tumour growth, using T cells derived from two different donors (left and right panels, respectively). Mice treated with mock T cells were co-treated ± 250 µg (~10 mg/kg) B6H12 (left panel) or 75 µg (~3 mg/kg) B6H12 (right panel). Mean ± SEM of n = 5 mice. [(a), (b), (d), (e), (g), (i), (j)] Two-way ANOVA test with Tukey’s multiple comparison test. ns = not significant. [(f), (h)] Log-rank Mantel-Cox test. ns = not significant. Source Data

We next assessed the antitumour effects of 47_WT_ versus 47_E_ T cells plus anti-CD47 therapy in multiple tumour models. In 143B, in which both CAR T cell and anti-CD47 therapy have minimal effect as monotherapies (Fig. 1a), we observed marked T-cell-mediated recruitment of macrophages into tumours (Fig. 5a,b). After treatment with B6H12, 47_WT_ CAR T cells were completely depleted, while 47_E_ CAR T cells persisted for multiple weeks (Fig. 5e,f and Extended Data Fig. 10a–e). Neither 47_WT_ or 47_E _CAR T cells alone, nor mock T cells or 47_WT_ CAR T cells paired with B6H12, mediated significant antitumour effects, whereas B6H12 plus 47_E_ CAR T cells induced a significant delay in tumour growth and an improvement in overall survival (Fig. 5g and Extended Data Fig. 10f–h). Similar results were seen with lower doses of anti-CD47, which may be associated with reduced toxicity in clinical studies^12^ (Extended Data Fig. 10i,j).

**Extended Data Fig. 11:**
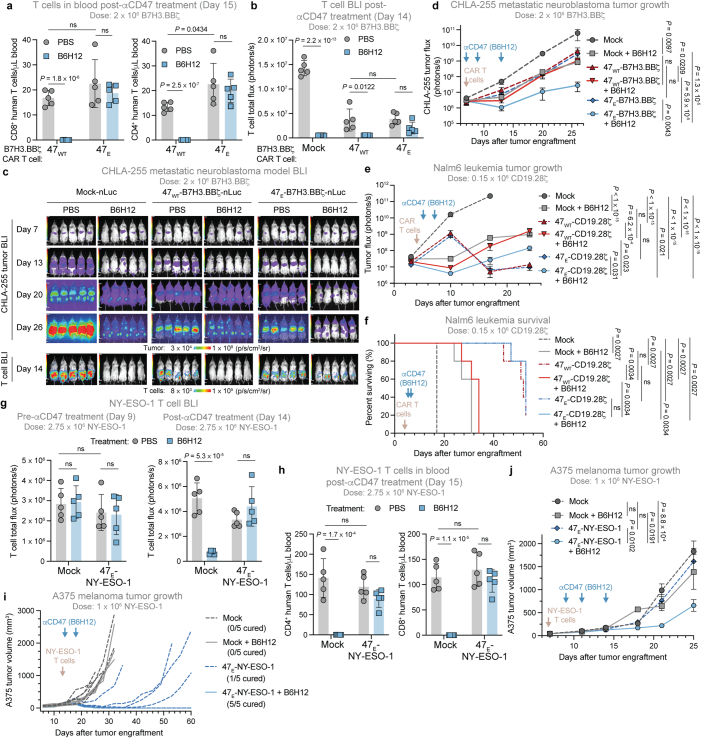
Expression of 47_E_ on tumour-targeting T cells permits pairing with anti-CD47 therapy and results in improved tumour control. (a) hCD8^+^ (left) and hCD4^+^ (right) T cells by flow cytometry from the blood of CAR-T treated mice on day 15 in CHLA-255 tumour bearing mice, treated with 47_WT_- or 47_E_-B7H3.BBζ-nLuc-CAR-T ± B6H12. Mean ± SD of n = 5 mice. (b) T cell BLI on day 14 in CHLA-255 tumour bearing mice, treated as in (a). Mean ± SD of n = 5 mice. (c) Images of CHLA-255 tumour progression (top four rows) and T cells on day 14 (bottom) by BLI, treated as in (a). (d) 47_WT_- or 47_E_-B7H3.BBζ-nLuc-CAR-T ± B6H12 treated CHLA-255 tumour growth by BLI. Mean ± SEM of n = 5 mice/arm. (e) 47_WT_- or 47_E_-CD19.28ζ-CAR-T ± B6H12 treated Nalm6 tumour growth by BLI. Mean ± SEM of n = 5 mice/arm. (f) 47_WT_- or 47_E_-CD19.28ζ-CAR-T ± B6H12 treated Nalm6 survival. n = 5 mice/arm. (g) T cell by BLI in A375 tumour bearing mice, treated with 47_E_-NY-ESO-1-Antares-TCR-T cells ± B6H12, before (day 9, left) and after (day 14, right) αCD47 treatment. Mean ± SD of n = 5 mice. (h) hCD4^+^ (left) and hCD8^+^ (right) T cells by flow cytometry from the blood in the A375 tumour bearing mice, treated as in (g). Mean ± SD of n = 5 mice. (i) 47_E_-NY-ESO-1-TCR-T ± B6H12 treated A375 tumour growth. Data are individual tumour growth traces of n = 5 mice/arm. (j) 47_E_-NY-ESO-1-TCR-T ± B6H12 treated A375 tumour growth, with T cells derived from a different donor than shown in (i). Mean ± SEM of n = 5 mice/arm. [(a), (b), (d), (e), (g), (h), (j)] Two-way ANOVA test with Tukey’s multiple comparison test. ns = not significant. [(f)] Log-rank Mantel-Cox test. ns = not significant.

We also paired B6H12 with 47_WT_ versus 47_E_ B7H3-BBζ CAR T cells in metastatic neuroblastoma and observed enhanced persistence and improved antitumour efficacy with 47_E_ CAR T cells plus B6H12 (Extended Data Fig. 11a–d) and enhanced antitumour efficacy with 47_E_ versus 47_WT_ CD19-28ζ CAR T cells plus B6H12 in mice bearing Nalm6-fLuc leukaemia (Extended Data Fig. 11e,f). Finally, in the A375 melanoma model, B6H12 depleted NY-ESO-1 T cells expressing endogenous CD47 (Fig. 1e), while 47_E_ NY-ESO-1 T cells persisted (Fig. 5h and Extended Data Fig. 11g,h). A375 tumour growth was minimally slowed by B6H12 plus mock T cells and treatment with 47_E_ NY-ESO-1 T cells alone led to initial tumour control, but ultimate tumour outgrowth. By contrast, mice treated with 47_E_NY-ESO-1 T cells and B6H12 demonstrated complete tumour control and cure in all of the mice treated (Fig. 5i and Extended Data Fig. 11i,j).

Together, these data demonstrate that CD47 blockade paired with 47_E_ expressed in therapeutic T cells protects T cells from macrophage-mediated phagocytosis and results in a considerable and sustained influx of macrophages within the TME, associated with T cell–macrophage cross-talk. The outcome is a strong antitumour synergy in solid, liquid and metastatic tumours, even at low doses and in conditions in which both single-agent therapies manifest no activity.

## Discussion

Previous studies reported that CD47 is necessary to prevent immune rejection^22^ and that CD47 overexpression combined with MHC knockout imparts resistance of CAR T cells to allogeneic immune rejection^13^. Our findings demonstrate that, even in the absence of immune rejection, CD47 is required for the survival of adoptively transferred T cells and CD47 overexpression improves CAR T cell persistence and efficacy (Fig. 1j,k). Anti-CD47 treatment in NSG mouse hosts induced rapid and complete macrophage-mediated depletion of adoptively transferred T cells (Fig. 1), which was sufficiently rapid and potent to mediate complete protection in a lethal CAR T cell toxicity model (Fig. 3), providing evidence to support testing of CD47–SIRPα blockers to mitigate toxicities resulting from adoptive T cell therapeutics, which could have immediate clinical benefits. These results align with data demonstrating that *CD47*knockout CAR T cells show limited persistence in xenograft models^23^ and observations of reduced total and antigen specific lymphocytes, increased susceptibility to infection and reduced susceptibility to autoimmunity in *Cd47* and *Sirpa* knockout mice^24,25^. Relevance to the clinical setting is provided by the observation of lymphopaenia in studies of magrolimab and evorpacept, both of which block CD47 engagement of SIRPα^10,12^.

Although T cell depletion was greatly enhanced by CD47 blockade in our studies, our data demonstrate that macrophage phagocytosis limits the persistence of adoptively transferred T cells even in the absence of CD47–SIRPα blockade (Fig. 1l and Extended Data Figs. 7h–l and 9j). Clinical scRNA-seq data also consistently identified CAR transcripts within myeloid cells (Fig. 2d). Together, these findings are consistent with a model in which macrophage phagocytosis has an important role in regulating T cell homeostasis. It is well recognized that CD47 blockade selectively depletes aged red blood cells^12,20^. We observed increased expression of ‘eat me’ signals and decreased expression of CD47 on T cells after prolonged culture (Fig. 2b and Extended Data Fig. 4d–h), raising the prospect that the CD47–SIRPα axis may similarly regulate the clearance of aged T cells. Future research is needed to better define the relationship between T cell activation and differentiation, and expression of pro-phagocytic and anti-phagocytic receptors, and phagocytosis susceptibility.

We observed that CAR T cells induce a rapid influx of macrophages into tumours (Fig. 5a,b), and that macrophage persistence is dependent on CAR T cell persistence. As a result, 47_E_ CAR T cell recipients manifested extensive cross-talk between myeloid cells and T cells in the TME, including induction of IL-12, CD40L and NF-κB signalling in T cells (Fig. 5c), which have been demonstrated to augment antitumour effects^1^. Thus, 47_E_ protected T cells from anti-CD47-mediated phagocytosis (Fig. 4f,g and Extended Data Fig. 7), while enhancing tumour phagocytosis, antigen presentation and inducing a pro-inflammatory TME. Additional studies are needed to test 47_E_ in fully immunocompetent systems to understand the impact on other immune cell types, including endogenous T and natural killer cells^26^. While B6H12 is a research-grade antibody, the CD47 variants generated in this study also ablated binding to the clinical grade antibody TJC4 (lemzoparlimab)^20^ (Fig. 4d,e), leading us to predict that 47_E_ could allow pairing of adoptive therapies with clinical grade anti-CD47 antibodies. Further enhancements could be developed for this approach^1,6^, including pairing with additional antibodies^27^ and inducing T cell secretion of CD47–SIRPα blockers^27,28^.

TAMs are among the most plentiful cells in the TME, and there has been great interest in harnessing their antitumour properties, but effective macrophage-modulating therapeutics for cancer are lacking^2^. Neither CSF1R and CCR2 inhibitors, which inhibit recruitment of monocytes to the TME and reduce or eliminate TAMs^4,5^, nor approaches to modulate macrophage states from those with immunosuppressive profiles, including the M2-like subset, toward those with more inflammatory profile have mediated clear therapeutic benefit^2,29^. Similarly, although systemic blockade of the CD47–SIRPα axis mediated antitumour effects in several preclinical models^6–9^, clinical benefit as single agents and in solid cancers is lacking^10,11^. The data presented here suggest that this conundrum may be explained by the double-edged sword that TAMs represent within the TME, whereby antitumour effects of manoeuvres designed to augment macrophage phagocytosis are offset by phagocytosis of tumour reactive T cells. Conversely, eliminating or reducing TAMs may diminish immunosuppression and phagocytosis of tumour infiltrating T cells, but these benefits are offset by loss of tumour phagocytosis by macrophages. Our data demonstrate that pairing 47_E_ CAR T therapy with anti-CD47 is an exciting prospect that could enhance the potency of adoptive T cell therapies, especially in solid cancers.

## Methods

### Cell lines

The Nalm6 B-ALL cell line was provided by D. Barrett and retrovirally transduced to express GFP and firefly luciferase. 143B osteosarcoma cells were acquired from the American Type Culture Collection (ATCC) and then retrovirally transduced with human CD19. The CHLA-255 neuroblastoma line was obtained and provided by R. Seeger and retrovirally transduced with GFP and firefly luciferase. MG63.3 cells were provided by C. Khanna and retrovirally transduced with GFP and firefly luciferase. D425 cells were provided by S. Chesier and retrovirally transduced to express GFP and firefly luciferase. Nalm6 and MG63.3 cells were originally obtained from ATCC. D425 cells were originally obtained from Sigma-Aldrich. A375 melanoma cells and Jurkat cells (clone E6-1) were obtained from ATCC. The 293GP retroviral packaging line was provided by the Surgery Branch (National Cancer Institute, National Institutes of Health). HEK293T lentiviral packaging cells were obtained from ATCC. Expi293 protein production cells were obtained from ATCC.

D425 cells were maintained in serum-free medium supplemented with B27 (Thermo Fisher Scientific), EGF, FGF (Shenandoah Biotechnology), human recombinant LIF (Millipore) and heparin (StemCell Technologies). Nalm6, 143B, A375, MG63.3, CHLA-255 and Jurkat cells were cultured in RPMI-1640 (Gibco). 293GP and HEK293T cells were cultured in DMEM (Gibco). Expi293 cells were cultured in Expi293 medium (Thermo Fisher Scientific). Cell line culture medium was supplemented with 10% FBS, 10 mM HEPES, 2mM l-glutamine, 100 U ml^−1^ penicillin and 100 μg ml^−1^ streptomycin (Gibco), except for the Expi293 medium. Short tandem repeat DNA profiling of all cell lines was conducted once per year (Genetica Cell Line testing). All cell lines were routinely tested for mycoplasma. Cell lines were cultured at 37 °C in a 5% CO_2_ environment.

### Source of primary human T cells and macrophages

Buffy coats from healthy donors were purchased from the Stanford Blood Center under an IRB-exempt-protocol. Leukopaks from healthy donors were purchased from StemCell Technologies. Primary human T cells were purified by negative selection using the RosetteSep Human T cell Enrichment kit (StemCell Technologies) and SepMate-50 tubes. T cells were cryopreserved at 2 × 10^7^ cells per ml in CryoStor CS10 cryopreservation medium (StemCell Technologies) until use. Primary peripheral monocytes were purified through successive density gradients using Ficoll (Sigma-Aldrich) and Percoll (GE Healthcare). Monocytes were then differentiated into macrophages by 7–9 days of culture in IMDM + 10% AB human serum (Life Technologies).

### Viral vector construction

All DNA constructs were visualized using SnapGene software (v.6.0.2; Dotmatics). All retroviral constructs were cloned into the MSGV1 retroviral vector^30^. The following CAR and TCR constructs used in this study were previously described: B7H3-BBζ^31^, GD2-BBζ^15^, CD19-BBζ^32^, HER2-BBζ^32^, CD19-28ζ^32^, HA-28ζ^33^ and NY-ESO-1^34^. B7H3-BBζ was previously generated by fusing, from N to C terminus, a human GM-CSF leader sequence, scFv derived from MGA271 in the VH-VL orientation and (GGGS)_3_ linker sequence, CD8α hinge and transmembrane sequence, and human 4-1BB and CD3ζ intracellular signalling domains. GD2-BBζ, HER2-BBζ and CD19-BBζ were generated previously by cloning scFvs derived from 14G2A, 4D5 and FMC63 antibodies, respectively, into the B7H3-BBζ vector in place of the MGA271 scFv. CD19-28ζ was generated previously by replacing the 4-1BB domain in CD19-BBζ with the intracellular signalling domain of human CD28. HA-28ζ was generated previously by replacing the FMC63 scFv with the 14G2a scFv containing a point mutation (E101K) followed by a spacer region derived from the CH2CH3 domains of IgG1. PIP-28ζ and PIP-BBζ were generated by replacing the FMC63 scFv with the 2.5F knottin^16,35^ followed by a Flag-tag sequence (DYKDDDDK) in the CD19-28ζ and CD19-BBζ vectors, respectively. The in vivo T cell activation reporter was constructed by cloning a sequence containing firefly luciferase into the pGreenFire1-NF-κB lentiviral vector (System Biosciences) under the NF-κB responsive promoter^32^. CD47 vectors were generated by inserting codon-optimized CD47 sequences (variant and WT) in place of the CD19-BBζ sequence. For in vivo tracking, CAR-nLuc plasmids were generated by replacing the stop codon in the CD3ζ with a sequence containing a porcine teschovirus-1 2A (P2A) ribosomal skipping sequence, followed by nanoluciferase^32^. Antares plasmids were generated by inserting the Antares sequence^36^ in place of the CD19-BBζ sequence. The NY-ESO-1 TCR construct was generated by inserting the NY-ESO-1 α chain, followed by a P2A sequence, followed by the β chain in place of CD19-BBζ.

### Virus production

Retroviral supernatant was packaged using 293GP cells and the RD114 envelope plasmid. In brief, 11 μg RD114 and 22 μg of the corresponding MSGV1 transfer plasmid were delivered to 293GP cells grown on 150 mm poly-d-lysine dishes (Corning) to 80% confluency by transient transfection with Lipofectamine 2000 (Thermo Fisher Scientific). The medium was replenished every 24 h. Virus production was performed side by side for comparable CAR, TCR and CD47 constructs. The retroviral supernatant was collected 48 and 72 h after transfection. The supernatants from replicate dishes were pooled, centrifuged to deplete cell debris and stored at −80 °C until use. Third-generation, self-inactivating lentiviral supernatant was similarly produced with HEK293T cells using 7 μg pMD2.G (VSVg) envelope, 18 μg pMDLg/pRRE (Gag/Pol), 18 μg pRSV-Rev and 20 μg of the corresponding transfer plasmids. All of the constructs were retroviral, except for the T cell NF-κB activation construct, which was lentiviral.

### CAR T and TCR T cell manufacturing

At day 0, primary human T cells were thawed and activated with anti-CD3/CD28 human T-Expander Dynabeads (Thermo Fisher Scientific) at a 3:1 or 1:1 bead to cell ratio. On day 2, virus-coated culture plates were prepared on non-tissue-culture-treated 12-well plates that had been precoated with RetroNectin (Takara Bio) according to the manufacturer’s instructions, by incubating with 1 ml of retroviral supernatant (2 × 10^7^–5 × 10^7^ TU ml^−1^) and centrifugation at 3,200 rpm and 32 °C for 2 h. The supernatant was subsequently aspirated off of the wells and 0.5 × 10^6^–1 × 10^6^ T cells were added in 1 ml of T cell medium comprising AIM V (Thermo Fisher Scientific), 5% FBS, 100 U ml^−1^ penicillin (Gibco), 100 mg ml^−1^ streptomycin (Gibco), 2 mM l-glutamine (Gibco), 10 mM HEPES (Gibco) and 40 U ml^−1^ rhIL-2 (Peprotech). After addition of the T cells, the plates were gently centrifuged at 1,200 rpm for 2 min then incubated for 24 h at 37 °C under 5% CO_2_. This transduction process was repeated on day 3 and day 4 (if necessary). Dynabeads were removed on day 4 or day 5 by magnetic separation. Cells were maintained between 0.4 × 10^6^ and 2 × 10^6^ cells per ml and expanded until day 10–12. Typically, T cells were transduced with CAR or TCR and Antares (if used) on day 2, and then CD47 variants on days 3 and 4.

### CRISPR–Cas9 KO of *CD47* and *AAVS1*

Ribonucleoprotein (RNP) was prepared using synthetic sgRNA with 2′-*O*-methyl phosphorothioate modification (Synthego) diluted in TE buffer at 120 μM. A total of 5 μl sgRNA was incubated with 2.5 μl duplex buffer (IDT) and 2.5 μg Alt-R *Streptococcus pyogenes* Cas9 Nuclease V3 (IDT) for 30 min at room temperature. Reactions (100 μl) were assembled with 5 million T cells or Jurkat cells, 90 μl P3 buffer (Lonza) and 10 μl RNP. Cells were pulsed with protocol EO115 using the P3 Primary Cell 4D-Nucleofector Kit and 4D-Nucleofector System (Lonza). Cells were recovered immediately with warm medium for 6 h before transduction with CAR or TCR. Cells were electroporated with RNP on day 2 after thaw and transduced later the same day. Guide sequences were as follows: *CD47*, 5′-AUGCUUUGUUACUAAUAUGG-3′; *AAVS1*, 5′-GGGGCCACUAGGGACAGGAU-3′.

### Flow cytometry analysis of mammalian cells

Cells were washed with FACS buffer (2% FBS in PBS) before staining. Staining was performed in FACS buffer for 30 min at 4 °C. In certain experiments, cells were first stained with Fixable Viability Dye eFluor 780 (eBioscience, 1:2,000) in PBS for 10 min at room temperature before washing with FACS buffer and staining with other antibodies. After staining, cells were then washed once with FACS buffer and analysed on the BD Fortessa system. FACSDiva (v.8.0.1; BD) software was used for data collection and FlowJo software (v.10.8.1; BD) was used for data analysis (gating strategies are shown in Supplementary Fig. 2).

Recombinant B7H3-Fc and HER2-Fc (both R&D systems, 1:400 dilution) were used to detect B7H3 and HER2 surface CAR, respectively. Likewise, anti-FMC63 idiotype antibody (Genscript, 1:400) was used to detect CD19 CARs, while anti-14G2a idiotype antibody (National Cancer Institute, 1:400) was used to detect GD2 and HA CARs. CAR detection reagents were fluorescently labelled using the DyLight 650 Microscale Antibody Labelling Kit (Thermo Fisher Scientific). Anti-DYKDDDDK tag (Flag tag, APC, L5, BioLegend, 1:400) was used to detect the PIP CAR. NY-ESO-1 TCR was detected with antibodies specific for Vβ13.1 (APC, H131, BioLegend, 1:100), the beta chain of the NY-ESO-1 TCR. CD47 was detected with B6H12^37,38^ (BV711 and PE, B6H12, BD, 1:100; APC, B6H12, Invitrogen, 1:100; or unlabelled, Bio X Cell, concentrations are listed in the figures), TJC4^20^ (unlabelled, produced in-house, concentrations are listed in the figures), Hu5F9^12^ (unlabelled, produced in-house, concentrations are listed in the figures), CV-1-Fc (unlabelled, ALX Oncology, concentrations are listed in the figures), mSIRPα-Fc (unlabelled, Sino Biological, concentrations are listed in the figures) or hSIRPα-Fc (unlabelled, Sino Biological, concentrations are listed in the figures), followed by detection with polyclonal anti-mouse or anti-human IgG antibodies (AF488 and AF647, polyclonal, Invitrogen, 1:500). mIgG1 isotype control antibodies (unlabelled, MPOC-21, Bio X Cell, 1:100 and PE, B11/6, Abcam, 1:100) were used as controls for B6H12 staining. The following antibodies were used for detection of cell surface proteins: calreticulin (PE, FMC 75, Abcam, 1:100); human CD4 (BUV 395, SK3, BD, 1:200); human CD8 (BUV 805, SK1, BD, 1:400); human CD45 (Per-CP-Cy5.5, HI30, Invitrogen, 1:50); human CD69 (BV421, FN50, BioLegend, 1:100); human CD39 (BV605, A1, BioLegend, 1:100); human TIM3 (BV510, F38-2E2, BioLegend, 1:100); human LAG3 (PE, 3DS223H, Invitrogen, 1:100); human PD1 (PE-Cy7, J105, Invitrogen, 1:100); human CD45RA (BV785, HI100, BioLegend, 1:100); human CD62L (BV605, DREG-56, BD, 1:100); human CD3 (BUV 737, SK7, BD, 1:100); mouse CD45 (BUV 805, I3/2.3, BD, 1:100); F4/80 (BV605, BM8, BioLegend, 1:100); CD11b (APC, M1/70, BioLegend, 1:50 and BUV 395, M1/70, BD, 1:100); human CD19 (BUV 496, SJ25C1, BD, 1:100). Annexin V was detected using the eBioscience Annexin V Apoptosis Detection Kit (Invitrogen) according to the manufacturer’s instructions.

### BLI analysis

Mice were administered 200 μl of 15 mg ml^−1^ d-luciferin for firefly luciferase imaging or a 1:40 dilution of Nano-Glo substrate (Promega, diluted in DPBS) for Antares and nanoluciferase imaging by intraperitoneal injection. Images were acquired on the IVIS (Perkin Elmer) or Lago (Spectral Instruments Imaging) imaging system 4 min after injection for fLuc and 8 min after injection for nLuc/Antares using 30 s exposures and medium binning. If saturated pixels were detected in the image, an additional image was acquired using the auto-expose setting. Total flux was measured using Living Image (v.4.7.3; Perkin Elmer) or Aura (v.4.0.7; Spectral Instruments Imaging) software with a region of interest around the body of each mouse. Only non-saturated images were used for quantification of BLI. Mice were randomized before T cell administration to ensure uniform distribution of tumour burden between groups. At the end of the experiment, all of the images were collected into a single sequence on Aura and set to the same luminescence scale.

### Recombinant protein cloning and production

The gWIZ vector with a BM40 signal peptide was used for protein expression. DNA encoding the Hu5F9 (magrolimab^12^) heavy chain with an hIgG1 Fc domain, Hu5F9 light chain, TJC4 (lemzoparlimab^20^) heavy chain with an hIgG1 Fc domain and TJC4 light chain were ordered from IDT. Heavy and light chains were individually cloned into an AscI/BamHI-digested gWIZ vector using Gibson assembly. Plasmids were transfected into Expi293F cells (Thermo Fisher Scientific) at a 1:1 ratio of heavy chain:light chain using ExpiFectamine according to the manufacturer’s instructions. Then, 5 days after transfection, the supernatant was collected, adjusted to pH 8.0 and sterile-filtered. Hu5F9 and TJC4 were then purified using recombinant Protein A-Sepharose 4B (Thermo Fisher Scientific) buffer-exchanged into PBS and concentrated using Amicon Centrifugal Filters (Millipore Sigma). To assess CD47 binding, cells were stained with Hu5F9 or TJC4 and then stained with labelled anti-human secondary antibodies (AF488 or AF647, Invitrogen, 1:500). B6H12^37,38^ and mIgG1 isotype control (MOPC-21) were acquired from Bio X Cell. CV-1 variants (ALX-222, CV-1-hIgG1 Fc; and ALX-90, CV-1-hIgG1 dead Fc) were acquired from ALX Oncology. Human SIRPα-mFc and mouse SIRPα-hFc were acquired from Sino Biologic.

### Animal models

NSG mice (NOD.Cg-Prkdc^scid^Il2rgt^m1Wjl^/SzJ) were purchased from the Jackson Laboratory and bred in-house under Stanford University APLAC-approved protocols. Healthy male and female mice that were used for in vivo experiments were aged between 6 and 10 weeks at tumour or T cell engraftment and were drug naive, and not involved in previous procedures. The mice were housed in sterile cages in a barrier facility at Stanford University at 22 °C and 50% humidity under a 12 h–12 h light–dark cycle. Veterinary Services Center (VSC) staff at Stanford University monitored the mice daily. Mice were euthanized when they manifested persistent hunched posture, persistent scruffy coat, paralysis, impaired mobility, greater than 20% weight loss, if tumours significantly interfered with normal bodily functions or if they exceeded limits designated in APLAC-approved protocols of 1.70 cm in any direction. According to the recommendations of VSC staff, mice with morbidities were supported with 500 μl subcutaneous saline, diet gel (DietGel 76A, ClearH2O) and wet chow. For all experiments, no sample size calculations were performed, but group sizes were determined by experience with well-established, previously published models^31,32,34,38^. Cages of mice that were previously engrafted with tumour were randomly assigned CAR T cell and anti-CD47 conditions for infusion, ensuring approximately equal distributions of tumour size between groups before treatment. Tumour engraftments and T cell infusions were performed by a technician who was blinded to treatments and expected outcomes.

### 143B osteosarcoma tumour model

0.5 × 10^6^ or 1 × 10^6^ 143B or 143B-CD19 cells (143B cells engineered to over-express CD19; 143B cells do not naturally express CD19) in 100 μl DPBS were injected into the tibial periosteum of six- to ten-week-old NSG male or female mice (engraftment dose indicated below for each specific study)^32^. Generally, 5 days after tumour implantation and after visual confirmation of tumour formation, mice were treated with HER2-BBζ CAR T cells, followed by two doses of B6H12. Tumour progression was monitored by measurement using callipers. Mice were euthanized according to the criteria described in the ‘Animal models’ section. Specifics for different iterations of the model presented are as follows:

CAR T cell + B6H12 studies (Fig. 1a, Supplementary Fig. 1a and Extended Data Fig. 1a,h): mice engrafted with 0.5 × 10^6^ 143B-CD19 cells were treated with 10 × 10^6^ Her2-BBζ CAR T cells by tail-vein injection on day 5. Mice were then treated twice with B6H12 (250 μg) or PBS by intraperitoneal injection on day 6 and day 10. T cells were quantified in the blood by flow cytometry on day 12.

PIP CAR T cell survival study (Fig. 3a): mice engrafted with 0.5 × 10^6^ 143B were treated with 10 × 10^6^ CD19-BBζ (non-tumour-targeting control), HER2-BBζ (tumour targeting control), PIP-28ζ or PIP-BBζ CAR T cells on day 5 by tail-vein injection.

PIP CAR T cell serum cytokine study (Fig. 3b): non-tumour-bearing mice or mice engrafted with 0.5 × 10^6^ 143B-CD19 were treated with 5 × 10^6^ CD19-BBζ (tumour-specific control), PIP-28ζ or PIP-BBζ CAR T cells, or mock T cells by tail-vein injection on day 4. Blood was collected for serum cytokine analysis on day 8 (4 days after CAR T cell administration).

47_E_ CAR T cell studies with high-dose B6H12 (Fig. 5e–g, Supplementary Fig. 1p and Extended Data Fig. 10a–h): mice engrafted with 1 × 10^6^ 143B-CD19 cells were treated with 4 × 10^6^ HER2-BBζ Antares CAR T cells with endogenous *CD47* KO and overexpressing either CD47 WT (47_WT_) or CD47(Q31P) (47_E_), or an equivalent number of mock-Antares T cells intravenously by tail-vein injection on day 5. Mice were then treated twice with B6H12 (250 μg) or PBS by intraperitoneal injection on day 7 and day 11. T cells were quantified by nanoluciferase BLI before (day 7) and after (day 13) anti-CD47 treatment and in the blood by flow cytometry on day 14.

47_E_ CAR T cell studies with low-dose B6H12 (Supplementary Fig. 1q and Extended Data Fig. 10i,j): mice engrafted with 0.5 × 10^6^ 143B-CD19 cells were treated with 4 × 10^6^ HER2-BBζ CAR T cells with endogenous *CD47* KO and overexpressing either 47_WT_ or 47_E_, or an equivalent number of mock T cells intravenously by tail-vein injection on day 5. The mice were then treated twice with B6H12 (75 μg or 25 μg) or PBS by intraperitoneal injection on day 6 and day 10. T cells were quantified in the blood by flow cytometry on day 12. Only those mice that were treated with 47_E_ CAR T cells were evaluated for antitumour efficacy in combination with B6H12.

### A375 melanoma tumour model

A total of 3 × 10^6^ A375 cells in 100 μl DPBS was injected into the flanks of NSG male or female mice aged 6–10 weeks^34^. Generally, 7 to 14 days after tumour implantation and after visual confirmation of tumour formation, mice were treated with NY-ESO-1 TCR T cells, followed by two or three doses of B6H12. Tumour progression was monitored by measurement using callipers. Mice were euthanized according to the criteria described in the Animal Models section. Specifics for different iterations of the model presented are as follows:

Low-dose NY-ESO-1 TCR T cell + B6H12 studies (Fig. 1d,e, Supplementary Fig. 1d and Extended Data Fig. 1i): mice were treated with 2 × 10^6^ NY-ESO-1 TCR T cells or an equivalent number of mock T cells intravenously by tail-vein injection on day 9 after tumour implantation. Mice were then treated twice with B6H12 (250 μg) or PBS by intraperitoneal injection on day 10 and 15. T cells were quantified in the blood by flow cytometry on day 17.

High-dose NY-ESO-1 TCR T cell + B6H12 studies (Supplementary Fig. 1d and Extended Data Fig. 1j,k): mice were treated with 5 × 10^6^ NY-ESO-1 TCR T cells or an equivalent number of mock T cells intravenously by tail-vein injection on day 7 after tumour implantation. Mice were then treated twice with B6H12 (250 μg) or PBS by intraperitoneal injection on day 9 and 13. T cells were quantified in the blood by flow cytometry on day 16.

47_E_ NY-ESO-1 TCR T cell quantification studies (Fig. 5h, Supplementary Fig. 1t and Extended Data Fig. 11g,h): 7 days after tumour implantation, mice were treated with 2.75 × 10^6^ NY-ESO-1-Antares TCR T cells with endogenous *CD47* KO and overexpressing 47_E_, or an equivalent number of mock-Antares T cells intravenously by tail-vein injection. Mice were then treated three times with B6H12 (250 μg) or PBS by intraperitoneal injection on days 9, 11 and 14. T cells were quantified by nanoluciferase BLI before (day 9) and after (day 14) anti-CD47 treatment and in the blood by flow cytometry on day 15.

47_E_ NY-ESO-1 TCR T cell antitumour efficacy studies (Fig. 5i, Supplementary Fig. 1u and Extended Data Fig. 11i,j): 7 days (T cell donor experiment 1; Extended Data Fig. 11j) or 14 days (T cell donor experiment 2; Fig. 5i and Extended Data Fig. 11i) after tumour implantation, mice were treated with 1 × 10^6^ NY-ESO-1-Antares TCR T cells with endogenous *CD47* KO and overexpressing 47_E_, or an equivalent number of mock-Antares T cells intravenously by tail-vein injection. Mice were then treated either: three times with B6H12 (250 μg) or PBS by intraperitoneal injection on days 9, 11 and 14 (experiment 1); or twice with B6H12 (250 μg) or PBS by intraperitoneal injection on days 15 and 19 (experiment 2).

### MG63.3 osteosarcoma tumour model

A total of 1 × 10^6^ MG63.3 cells in 100 μl DPBS was injected into the tibia periostea of NSG male or female mice aged 6–10 weeks^31^. Starting 15 days after tumour implantation and after visual confirmation of tumour formation, the mice were treated with 400 μg of B6H12 or PBS three times per week by intraperitoneal injection. On day 21, the mice were treated intravenously with 10 × 10^6^ GD2-BBζ or B7H3-BBζ CAR T cells or no T cells. Tumour progression was measured using digital callipers twice per week. Mice were euthanized according to the criteria described in the ‘Animal models’ section (Supplementary Fig. 1b). For T cell quantification experiments, mice engrafted orthotopically with 1 × 10^6^ MG63.3 cells were treated intravenously with 10 × 10^6^ B7H3-BBζ CAR T cells on day 15 with or without 3 doses of B6H12 treatment (400 µg per dose; intraperitoneal). Blood and tumours were collected on day 30 after tumour engraftment.

### D425 medulloblastoma tumour model

Mice (aged 6–10 weeks) were anaesthetized with 3% isoflurane (Minrad International) in an induction chamber^31^. Anaesthesia on a stereotactic frame (David Kopf Instruments) was maintained at 2% isoflurane delivered through a nose adaptor. D425 medulloblastoma cells were injected at coordinates 2 mm posterior to lambda on midline and 2 mm deep using a blunt-ended needle (75 N, 26 s gauge/2 inch/point style 2, 5 μl; Hamilton). Using a microinjection pump (UMP-3; World Precision Instruments), 0.2 × 10^6^ D425-GFP-fLuc cells were injected in a volume of 3 μl at 30 nl s^−1^. After leaving the needle in place for 1 min, it was retracted at 3 mm min^−1^. Then, 4 days after tumour implantation and after confirmation of tumour formation by bioluminescence, mice were randomized and treated with no T cells (B6H12 only group), or 10 × 10^6^ B7H3-BBζ CAR^+^ T cells or an equivalent number of non-tumour targeting CD19-BBζ CAR^+^ T cells intravenously by tail-vein injection. Starting on day 4, the mice were also treated with 400 μg of B6H12 or PBS three times per week by intraperitoneal injection. Tumour progression was monitored by firefly luciferase BLI (Supplementary Fig. 1c). In Extended Data Fig. 1c,d, CD19-BBζ and B7H3-BBζ treatments are reproductions of previously published data^31^, included for comparison with B7H3-BBζ + B6H12, as these arms were all run simultaneously in the same experiment.

### Nalm6 leukaemia tumour models

A total of 1 × 10^6^ Nalm6-GFP-fLuc cells in 200 μl DPBS was implanted by tail-vein injection into NSG male or female mice aged 6–10 weeks^32^. Generally, four days after tumour implantation and after confirmation of tumour formation by BLI, mice were treated with CD19-BBζ or CD19-28ζ CAR T cells, followed by doses of anti-CD47. Tumour progression was monitored by fLuc BLI measurement. Mice were euthanized according to the criteria described in the ‘Animal models’ section. Specifics for different iterations of the model presented are as follows:

High-dose CAR T cell + B6H12 studies (Supplementary Fig. 1e and Extended Data Fig. 1l,n,p,s): mice engrafted with 1 × 10^6^ Nalm6-GFP-fLuc cells were treated with B6H12 (400 μg) or PBS by intraperitoneal injection three times per week, starting on day 3. Mice were then treated with 1 × 10^6^ CD19-28ζ-nLuc CAR T cells by tail-vein injection on day 4. T cells and tumours were quantified by BLI weekly.

Low-dose CAR T cell + B6H12 studies (Supplementary Fig. 1f and Extended Data Fig. 1m–o,q,r): mice engrafted with 1 × 10^6^ Nalm6-GFP-fLuc cells were treated with 0.15 × 10^6^ CD19-28ζ-nLuc CAR T cells by tail-vein injection on day 4. Mice were treated twice with B6H12 (250 μg) or PBS by intraperitoneal injection on day 5 and 7. T cells and tumours were quantified by BLI weekly.

High-dose CAR T cell + CV-1 studies (Fig. 1f,g, Supplementary Fig. 1g and Extended Data Fig. 2a,b,e): mice engrafted with 1 × 10^6^ Nalm6-GFP-fLuc cells were treated with 1 × 10^6^ CD19-BBζ-nLuc CAR T cells by tail-vein injection on day 4. Mice were treated with CV-1-Fc (ALX-90; dead Fc; 400 μg) or PBS by intraperitoneal injection three times per week starting on day 5. T cells and tumours were quantified by BLI weekly.

Low-dose CAR T cell + CV-1 studies (Supplementary Fig. 1h and Extended Data Fig. 2c–f): mice engrafted with 1 × 10^6^ Nalm6-GFP-fLuc cells were treated with 0.1 × 10^6^ CD19-28ζ-nLuc CAR T cells by tail-vein injection on day 4. Mice were treated with CV-1-Fc (ALX-90; dead Fc; 400 μg) or PBS by intraperitoneal injection three times on days 5, 7 and 10. T cells and tumours were quantified by BLI twice weekly.

Low-dose 47_KO_ CAR T cell studies (Fig. 1h,i and Extended Data Fig. 2h,i): mice engrafted with 1 × 10^6^ Nalm6-GFP-fLuc cells were treated with 0.15 × 10^6^ CD19-28ζ-nLuc CAR T cells with endogenous *CD47* KO (47_KO_), *CD47* KO with overexpression of CD47 WT (47_WT_), or an equivalent number of mock T cells by tail-vein injection on day 4. Mice were treated twice with B6H12 (250 μg) or PBS by intraperitoneal injection on days 5 and 7. Tumours were quantified by BLI weekly. T cells were quantified by BLI on day 11.

Low-dose 47_E_ CAR T cell + B6H12 studies (Supplementary Fig. 1s and Extended Data Fig. 11e,f): mice engrafted with 1 × 10^6^ Nalm6-GFP-fLuc cells were treated with 0.15 × 10^6^ CD19-28ζ-nLuc CAR T cells with endogenous *CD47* KO and overexpressing either 47_WT_ or 47_E_, or an equivalent number of mock T cells by tail-vein injection on day 4. Mice were treated twice with B6H12 (250 μg) or PBS by intraperitoneal injection on days 5 and 7. Tumours were quantified by BLI weekly.

### T cell depletion model

NSG male or female mice (aged 6–10 weeks) were implanted with 2 × 10^6^ or 5 × 10^6^ CD19-28ζ-nLuc CAR T cells with endogenous *CD47* KO and overexpressing either 47_WT_, 47_E_ or no additional protein (47_KO_) by tail-vein injection (day 0). Mice were then treated twice with B6H12 (250 μg) or PBS by intraperitoneal injection on days 3 and 5. T cells were quantified by nanoluciferase BLI before (2 × 10^6^ dose, day 2; 5 × 10^6^ dose, day 3) and after (2 × 10^6^ dose, day 9; 5 × 10^6^ dose, day 7) anti-CD47 treatment and in the blood by flow cytometry (2 × 10^6^ dose, day 7; 5 × 10^6^ dose, day 6). For isotype control studies (Extended Data Fig. 1f,g), mice were implanted with 5 × 10^6^ CD19-28ζ CAR T cells by tail-vein injection (day 0) and were then treated with B6H12 (250 μg), mIgG1 isotype control (250 μg) or PBS by intraperitoneal injection on day 1. T cells were quantified in the blood by flow cytometry on day 5. Mice were euthanized according to the criteria described in the ‘Animal models’ section at the conclusion of the experiment (Supplementary Fig. 1i,m).

### PIP CAR toxicity model

PIP CAR vectors were made as described in the viral vector construction section. NSG male or female mice (aged 6–10 weeks) were treated with the PIP CAR T or control CD19-BBζ, HER2-BBζ or mock T cells at the dose indicated in the figure (10 × 10^6^, 5 × 10^6^ or 2 × 10^6^ CAR T cells) by tail-vein injection. Mice in the PIP-28ζ and PIP-BBζ groups experienced rapid onset of toxicity (within 1–5 days, depending on dose) observed clinically as a hunched posture, scruffy coat, slow movement, dehydration and weight loss. Treatment-related toxicity was monitored by weight change, which was measured before T cell administration and 1–2× per week thereafter. The percentage weight change was calculated as follows: percentage weight change = ((weight at time *x*/initial weight) − 1) × 100. Mice died from toxicity or were euthanized if they reached 20% weight loss or showed clinical signs of severe toxicity, as described in the ‘Animal models’ section. For assessment of T cell localization and activation, CD19-28ζ-nLuc or PIP-28ζ-nLuc CAR T cells were transduced with a firefly luciferase reporter under control of an NF-κB-inducible promoter. Mice were implanted with 5 × 10^6^ CAR T cells and imaged daily with Nano-GLO substrate (nLuc; total CAR T) and luciferin (fLuc; active CAR T), with each substrate dose separated by 12 h. For organ BLI analysis, 4 days after treatment with 5 × 10^6^ CD19-28ζ-nLuc or PIP-28ζ-nLuc, the mice were injected with either Nano-GLO substrate (nLuc; total CAR T) or luciferin (fLuc; active CAR T) and euthanized 10 min after injection. Organs were collected according to standard procedures and imaged using BLI on the IVIS machine (Perkin Elmer) in six-well plates. For safety-switch models, mice were dosed with 2 × 10^6^ PIP-28ζ or CD19-28ζ CAR T cells intravenously. Mice treated with PIP-28ζ CAR T cells were administered 250 µg B6H12 or PBS over three consecutive days (days 2, 3 and 4), 2 days after CAR administration (day 0). Blood was collected for serum cytokine analysis on day 4 after CAR administration (Supplementary Fig. 1k).

### CHLA-255 neuroblastoma metastatic tumour model

Six- to ten-week-old NSG male or female mice were implanted with 1 × 10^6^ CHLA-255-GFP-fLuc cells by tail-vein injection^38^. The, 7 days after tumour implantation and after confirmation of tumour formation by BLI, mice were randomized and treated with 2 × 10^6^ B7H3-BBζ-nLuc CAR T cells with endogenous *CD47* KO and overexpressing 47_WT_ or 47_E_ or an equivalent number of mock (non-transduced) T cells intravenously by tail-vein injection. Mice were then treated three times with B6H12 (250 μg) or PBS by intraperitoneal injection on days 7, 9 and 13. Tumour progression was monitored by firefly luciferase BLI. T cells were quantified by nanoluciferase BLI after anti-CD47 treatment on day 14 and in the blood by flow cytometry on day 15. Mice were euthanized according to the criteria described in the ‘Animal models’ section (Supplementary Fig. 1r).

### CAR T cell GvHD model

NSG male or female mice (aged 6–10 weeks) were implanted with 1 × 10^6^ Nalm6-GFP-fLuc cells by tail-vein injection. The mice were then treated with 10 × 10^6^ CD19-BBζ CAR T cells on day 4. Half of the cohort of mice received three doses of B6H12 (250ug) over 3 days after CAR T cell administration. Mice were monitored for tumour growth by BLI and signs of GvHD, such as alopecia, dyskeratosis and weight loss^39,40^. Spleens and skin were extracted surgically. Skin sections were prepared as slides and stained with H&E using the standard method (Supplementary Fig. 1l).

### Isolation of T cells from spleens and tumours

Spleens and tumours were collected and mechanically dissociated using the gentleMACS dissociator (Miltenyi). Single-cell suspensions were made by passing spleens and tumours through a 70 μm cell strainer (Thermo Fisher Scientific), depleting red blood cells by ACK lysis (Quality Biological), and further filtration through flow cytometry filter tubes with 35 μm cell strainer caps (Falcon). Single-cell suspensions were then frozen in CryoStor buffer (StemCell Technologies) in liquid nitrogen, or stained and run directly on the flow cytometry system.

### Quantification of T cells and cytokines from the blood

Mouse blood was collected from the retro-orbital sinus into Microvette blood collection tubes with EDTA (Thermo Fisher Scientific). Red blood cells were depleted by ACK lysis (Quality Biological), followed by two washes with FACS buffer (PBS + 2% FBS). The samples were stained and mixed with CountBright Absolute Counting beads (Thermo Fisher Scientific) before flow cytometry analysis. IL-2 and IFNγ cytokine levels in blood were quantified using LEGENDPlex immunoassays (BioLegend) according to the manufacturer’s instructions from serum collected after centrifuging blood samples at 3,000 rpm for 10 min. Negative cytokine values were set to 0.

### IncuCyte tumour killing assays, cytokine analysis and T cell activation marker detection

A total of 5 × 10^4^ GFP-labelled tumour cells was cocultured with 5 × 10^4^ CAR T cells in 200 μl RPMI supplemented with 10% FBS, 10 mM HEPES, 2mM l-glutamine, 100 U ml^−1^ penicillin and 100 μg ml^−1^ streptomycin. For conditions with B6H12, a concentration of 10 µg ml^−1^ was used. Triplicate wells were plated in 96-well flat-bottom plates for each condition. Tumour fluorescence was monitored every 2–3 h with a ×10 objective using the IncuCyte S3 Live-Cell Analysis System (Sartorius), housed in a cell culture incubator at 37 °C and 5% CO_2_, set to take 4 images per well at each timepoint. The total integrated GFP intensity was quantified using the IncuCyte basic analyzer software feature (IncuCyte S3 v.2019B Rev2; Sartorius). Data were normalized to the first timepoint and plotted as the fold change in tumour fluorescence over time. For cytokine secretion and T cell marker analysis, cocultures were set up as described above except in 96-well round-bottom plates. After approximately 24 h, the plates were centrifuged to pellet cells and 150 µl of supernatant was collected and stored at −20 °C until analysis, while the cell pellets were immediately processed for flow cytometry. IFNγ and IL-2 levels in the coculture supernatants were quantified by ELISA (Human ELISA MAX Deluxe, BioLegend) according to the manufacturer’s instructions. Negative cytokine values were set to 0. Absorbance values were measured using the Synergy H1 Hybrid Multi-Mode Reader with Gen5 software (v.2.00.18; BioTek). For analysis of T cell markers after activation by tumour cells, pellets from centrifuged plates were pooled together for triplicate wells, stained and analysed using flow cytometry. Coculture experiments were set-up using day 10 T cells.

### Macrophage depletion and peritoneal lavage

NSG male or female mice (aged 6–10 weeks) were pretreated with intravenous injection with 200 µl of clodronate liposomes (Liposoma), followed by 400 µg of anti-mouse-CSF1R (AFS98; Bio X Cell) by intraperitoneal injection^38^. Mice were treated with 400 µg of anti-CSF1R three times per week for the duration of the experiment. Then, 6 days after clodronate treatment, the mice were administered with 2 × 10^6^ CD19-28ζ-nLuc CAR T cells intravenously, followed by 250 µg B6H12 intraperitoneally on day 7. T cells were quantified by nanoluciferase BLI before (day 7) and after (day 9) anti-CD47 treatment. Peritoneal lavage was performed on day 13 with 10 ml of FACS buffer and a 25 gauge needle. Peritoneal cells were collected, washed with FACS buffer and stained, before being run on the flow cytometry system (Supplementary Fig. 1j).

### Phagocytosis assay

For all flow-based in vitro phagocytosis assays, T cells and human macrophages were co-cultured at a ratio of 2:1 (for example, 100,000 T cells:50,000 macrophages) in ultra-low-attachment 96-well U-bottom plates (Corning) in serum-free RPMI (Thermo Fisher Scientific). T cells were labelled with CFSE (Invitrogen) by suspending cells in PBS (5 µM working solution) according to the manufacturer’s instructions for 20 min at 37 °C protected from light and washed twice with 20 ml of FBS-containing medium before co-culture. Cells were then either incubated alone or in the presence of anti-CD47 (B6H12; Bio X Cell) or mIgG1 isotype control (MOPC-21; Bio X Cell) at a concentration of 10 μg ml^−1^. T cells and antibodies were incubated for 30 min in a humidified 5% CO_2_ incubator at 37 °C. Plates were washed twice; human macrophages were added to the plate; and plates were incubated for 1–2 h at 37 °C. Phagocytosis was stopped by washing with 4 °C PBS and centrifugation at 1,450 rpm before the cells were stained with Live/Dead stain and anti-CD11b (APC, M1/70, BioLegend, 1:50). Assays were analysed by flow cytometry, and phagocytosis was measured as the number of CD11b^+^ and CFSE^+^ macrophages, quantified as a percentage of the total CD11b^+^ macrophages and normalized to the control condition.

For IncuCyte-based in vitro phagocytosis assays, T cells and human macrophages were co-cultured at a ratio of 2:1 (for example, 100,000 T cells:50,000 macrophages) in 96-well flat-bottom plates (Corning) in RPMI supplemented with 10% FBS, 10 mM HEPES, 2mM l-glutamine, 100 U ml^−1^ penicillin and 100 μg ml^−1^ streptomycin. T cells were labelled with pHrodo Red dye (Invitrogen) by incubating T cells at 1 × 10^6^ cells per ml with a working concentration of pHrodo Red of 30 ng ml^−1^ in PBS for 1 h at 37 °C in the dark in a humidified 5% CO_2_ incubator. The labelling reaction was quenched and excess dye was washed away by washing twice with complete medium. Cells were then either incubated alone or in the presence of anti-CD47 (B6H12; Bio X Cell) at a concentration of 10 μg ml^−1^ in serum-free RPMI. T cells and antibodies were incubated for 30 min in a humidified 5% CO_2_ incubator at 37 °C, before being washed twice with complete medium. Macrophages were added to each well and allowed to adhere for 2 h in a humidified 5% CO_2_ incubator at 37 °C. After 2 h, labelled T cells were added to the plate at a 2:1 T cell:macrophage ratio. pHrodo Red fluorescence due to phagocytosis was monitored after 3 h with a ×10 objective using the IncuCyte S3 Live-Cell Analysis System (Sartorius), housed in a cell culture incubator at 37 °C and 5% CO_2_, set to take four images per well at each timepoint. Total integrated red fluorescence intensity was quantified using the IncuCyte basic analyzer software feature (IncuCyte S3 v.2019B Rev2; Sartorius).

### Confocal microscopy of T cell–macrophage interactions

CD19-28ζ CAR T cells were labelled with pHrodo Red dye as described above. pHrodo-Red-labelled T cells were then labelled with DiO Vybrant lipophilic dye (Invitrogen) according to the manufacturer’s instructions in PBS for 2 min at 37 °C in the dark. Macrophages were labelled with DiD Vybrant dye (Invitrogen) according to the manufacturer’s instructions in PBS for 15 min at 37 °C in the dark. After dye labelling, cells were washed three times with complete medium to remove excess dye. T cells were then incubated with B6H12 at 10 μg ml^−1^ for 20 min at 37 °C in PBS, followed by two washes with complete medium. Labelled T cells and macrophages embedded in a collagen matrix (Cellmatrix type I-A, FUJIFILM Wako chemicals) at a ratio of 2:1 (for example, 1,000,000 T cells:500,000 macrophages) in a 24-well glass-bottom culture plate (Mattek). Four-dimensional (*x*,*y*,z,t) live confocal imaging was performed on a confocal laser-scanning microscope (Zeiss LSM900). Images were analysed using Imaris software (v.10.0; Oxford Instruments).

### Quantification of CD47 expression on tumour and T cells using QuantiBrite

CD47 expression was quantified using an anti-CD47-PE antibody (B6H12, BD, 1:20) and a QuantiBrite PE Quantitation Kit (BD) according to the manufacturer’s instructions^41^. CD19-28ζ CAR T cells were produced as described above, except that cells were kept in culture 1 day after thawing before activation with anti-CD3/CD28 beads. T cells were analysed by flow cytometry on day 0 (before activation; 1 day after thaw), day 4 (immediately after removal from bead activation), day 7 and day 11 (average time of transfer in vivo). T cells were stained with anti-hCD4 (BUV 395, SK3, BD, 1:200), anti-hCD8 (BUV 805, SK1, BD, 1:400), anti-hCD47 or mIgG1 isotype control (PE, B11/6, Abcam, 1:20), anti-hCD45RA (BV785, HI100, BioLegend, 1:100) and anti-hCD62L (BV605, DREG-56, BD, 1:100) antibodies. T cell differentiation subtypes were defined as follows: T naive (CD45RA^+^CD62L^+^), T central memory (CD45RA^−^CD62L^+^), T effector memory (CD45RA^−^CD62L^−^) and T effector memory re-expressing CD45RA (CD45RA^+^CD62L^−^). Tumour cells were stained with only anti-hCD47 or mIgG1 isotype control. Molecules of CD47 were calculated according to the QuantiBrite kit instructions using extrapolation from MFI signals of BD QuantiBrite-PE beads with known quantities of PE. The degree of labelling for anti-CD47-PE (BD, 2040745) was determined experimentally as 0.842 molecules of dye per antibody, using the maximum absorbance at 566 nm, the extinction coefficient for PE (1,863,000 M^−1^ cm^−1^) and the listed antibody concentration.

### Imaging of patient CSF samples

A CSF cytospin preparation was collected from a patient treated with axicabtagene ciloleucel (axi-cel) CD19-28ζ CAR T cell therapy, stained with Wright-Giemsa and imaged by microscopy at ×1,000 magnification, capturing histiocytes with engulfed lymphocytes^42^ (raw images are shown in Supplementary Fig. 3).

### Single-cell analysis of patient samples

Two datasets were reanalysed: ref. ^14^ (GSE168940), including scRNA-seq data collected from nine patients with LBCL treated with axi-cel CD19-28ζ CAR T cell therapy, where 50,000–70,000 CAR T cells (single live CD4^+^ or CD8α^+^CD235a^−^CAR^+^ events) were FACS sorted to ≥95% purity; and ref. ^15^ (GSE186802), including scRNA-seq data collected from four patients with DMG treated with GD2.BBζ CAR T cell therapy, with cells derived from CSF. Both datasets were analysed on the 10x Genomics platform^14,43^. Where indicated, previously annotated CAR-mRNA-expressing cells were used.

### Histology of tissue samples

The tissues assessed include skin and lung. Tissues were collected and immersion-fixed in 10% neutral-buffered formalin. After fixation, tissues were routinely processed, embedded in paraffin, sectioned at 5.0 μm and routinely stained with haematoxylin and eosin (H&E). Tissues were visualized using the Olympus BX43 upright bright-field microscope, and images were captured using the Olympus DP27 camera and cellSens software (v.3.2; Olympus Life Science).

### Yeast surface display vectors

A DNA sequence encoding the CD47 Ig-like domain (Gln19–Ser135) was cloned into the pCTCON2 yeast-surface display vector (Addgene) using the NheI and BamHI sites. The pFreeNTerm (pFNT) vector was based on the pCL backbone^44^, designing an intrinsic NheI cutsite into the Aga2p signal sequence as the 5′ cloning site and using a MluI cutsite prior to a 3×Gly_4_Ser linker as the 3′ cloning site. Variants of the CD47 Ig-like domain (Gln19–Ser135) were cloned into the pFNT yeast-surface display vector using these NheI and MluI sites.

### Flow cytometry analysis of yeast surface display constructs

*Saccharomyces cerevisiae* (strain, EBY100; ATCC) yeast were transformed with pCTCON2 or pFNT plasmids and selected on SD-CAA-Agar plates. Yeast (~100,000 per sample) were grown and induced in SG-CAA, and binding was set up over a range of soluble ligand concentrations in PBS containing 1 mg ml^−1^ BSA (BPBS), taking into account ligand depletion and equilibrium time^45^. After incubation with binding partner, yeast cells were washed once with BPBS, then incubated with a 1:5,000 dilution of chicken anti-MYC antibody (polyclonal, Invitrogen) for pCTCON2 displayed proteins, and incubated for 30 min at 4 °C in the dark. After primary addition, the samples were washed once with BPBS, and secondary antibodies were added. Expression was detected with a 1:500 dilution of goat anti-chicken Alexa Fluor 488 (polyclonal, Invitrogen) or Alexa Fluor 647 (polyclonal, Abcam). For pFNT displayed proteins, co-displayed GFP was used to monitor expression. Binding of proteins with mouse Fc domains (hSIRPα, B6H12) was detected with a 1:500 dilution of goat anti-mouse Alexa Fluor 488 or Alexa Fluor 647 (polyclonal, Invitrogen). Binding of proteins with a human Fc domain (CV-1 (ALX-222), mSIRPα, Hu5F9, TJC4) was detected using a 1:500 dilution of goat anti-human Alexa Fluor 488 or Alexa Fluor 647 (polyclonal, Invitrogen). Secondary antibodies were incubated for 15 min at 4 °C in the dark. After secondary incubation, the samples were washed once with BPBS, pelleted and left pelleted on ice until analysis. The samples were analysed by resuspending them in 50 μl of BPBS and running flow cytometry on the BD Accuri C6 (BD Biosciences) system. Accuri C6 software (v.1.0.264.21; BD) was used for data collection and FlowJo software (v.10.8.1; BD) was used for data analysis (gating strategies are shown in Supplementary Fig. 2). The samples were gated for bulk yeast cells (forward scatter (FSC) versus side scatter (SSC)) and then for single cells (FSC-height versus FSC-area). Expressing yeast were determined and gated through the C-terminal MYC tag or GFP detection. The geometric mean of the binding fluorescence signal was quantified from the expressing population and used as a raw binding value. When comparing binding signals, the average fluorescence expression signal was quantified for different protein variants and used to normalize binding signal. To determine the ‘fraction bound’, binding signals were divided by the signal derived from the highest concentration of binding partner used, or that derived from binding to WT CD47. To calculate *K*_d_ values, data were analysed in Prism (v.9.5.1, GraphPad) using a nonlinear regression curve fit.

### Yeast surface display library generation, sorting and sequencing

CD47 was expressed in *S. cerevisiae* (strain, EBY100; ATCC) as a genetic fusion to the agglutinin mating protein Aga2p in the pCTCON2 vector. An error-prone PCR library was created using the CD47 Ig-like domain (Gln19 to Ser135) as a template and mutations were introduced using the Gene Morph II random mutagenesis kit (Agilent) according to the manufacturer’s instructions. Separate PCRs were performed using various concentrations of Mutazyme II enzyme. Products from these reactions were purified by gel electrophoresis, pooled and amplified with standard PCR using Phusion polymerase (New England BioLabs). Purified mutant DNA and linearized pCTCON2 plasmid were electroporated into EBY100 yeast, where they were assembled in vivo through homologous recombination. We estimated 5 × 10^7^ variants for the library, determined by dilution plating and colony counting. Yeast were grown in SD-CAA medium and induced for CD47 protein expression by growth in medium containing 90% SG-CAA and 10% SD-CAA overnight^45^. Yeast displaying CD47 variants were isolated by FACS using the SONY SH800S cell sorter (SONY; SH800S cell sorter software v.2.1.5) and analysed using the BD Accuri C6 flow cytometer (BD Biosciences; BD Accuri software v.1.0.264.21). Gating strategies are shown in Supplementary Fig. 2. Data were analysed using FlowJo software (v.10.8.1, BD). Screens were performed using equilibrium binding conditions where yeast were incubated at room temperature in BPBS with the following concentrations of B6H12 or CV-1 (ALX-222) for 2 h. For negative sorts to B6H12, the CD47-expressing, but non-binding populations of yeast were collected. For positive sorts to CV-1, the CD47-expressing and binding populations of yeast were collected (Extended Data Fig. 6g). Sort 1, negative sort, 500 pM B6H12; sort 2, negative sort, 5 nM B6H12; sort 3, positive sort, 20 nM CV-1; sort 4, negative sort, 20 nM B6H12; sort 5, negative sort, 50 nM B6H12; sort 6, positive sort, 10 nM CV-1. After incubation with B6H12 or CV-1, yeast was pelleted, washed and labelled with fluorescent antibodies as described above before sorting. Sorted yeast clones were propagated, induced for CD47 expression and subjected to iterative rounds of FACS as described above. After each round of screening, plasmid DNA was recovered using the Zymoprep yeast plasmid miniprep I kit (Zymo Research), transformed into DH10B electrocompetent cells (Thermo Fisher Scientific) and isolated using the GeneJET plasmid miniprep kit (Thermo Fisher Scientific). Sequencing was performed by ELIM Biopharmaceuticals.

### CD47 structure modelling

CD47 structures were downloaded from the Protein Data Bank (PDB) and analysed using PyMol (v.2.5.8; Schrödinger). The CD47–hSIRPa structure used was PDB 2JJS (ref. ^18^). The CD47–B6H12 structure used was PDB 5TZU (ref. ^19^).

### 143B in vivo phagocytosis model

A total of 1 × 10^6^ 143B cells in 100 μl DPBS was injected into the tibia periosteum of NSG mice (aged 6–10 weeks). Then, 20 days after tumour implantation, mice were treated with 3 × 10^6^ CFSE-labelled HER2-BBζ CAR T cells intratumourally. Before administration, T cells were labelled with CFSE (5 µM working solution), according to the manufacturer instructions, with cells labelled at a concentration of 10^7^ cells per ml in PBS for 5 min. Cells were washed in complete medium, and then incubated with or without 10 µg ml^−1^ B6H12 in PBS for 20 min at 37 °C. Cells were resuspended in 80 µl of PBS/tumour for intratumoural injection. Immediately after T cell administration, the mice were then treated with B6H12 (250 µg) or PBS by intraperitoneal injection. Tumours were collected 16 h later on day 21 after tumour implantation. Tumours were mechanically dissociated using the gentleMACS dissociator (Miltenyi). Single-cell suspensions were made by passing tumours through a 70 μm cell strainer (Thermo Fisher Scientific), depleting red blood cells by ACK lysis (Quality Biological) and further filtration through flow cytometry filter tubes with 35 μm cell strainer caps (Falcon). Single-cell suspensions were subsequently stained for flow cytometry to detect CFSE^+^ T cells and macrophages from dissociated tumours (see below) (Supplementary Fig. 1n).

### 143B correlative study and tumour dissociation

A total of 1 × 10^6^ 143B cells in 100 μl DPBS was injected into the tibia periosteum of NSG mice (aged 6–10 weeks). Then, 13 days after tumour implantation and after visual confirmation of tumour formation, the mice were treated with 4 × 10^6^ HER2-BBζ CAR T cells with endogenous *CD47* KO and overexpressing either 47_WT_ or 47_E_, an equivalent number of mock T cells intravenously by tail-vein injection, or no T cells. The mice were then treated twice with B6H12 (250 µg) or PBS by intraperitoneal injection on day 15 and day 19. Tumours were collected at day 21 after tumour implantation (day 8 after CAR T cell treatment). Tumours were split with a razor, with one section being fixed in 10% paraformaldehyde, and the other mechanically dissociated as described above, before being stained for flow cytometry and FACS. Formaldehyde-fixed tumour had paraformaldehyde removed after 24 h and replaced with 70% ethanol for long-term storage. Tumour sections were then formalin-fixed and paraffin-embedded according to the standard protocol (Supplementary Fig. 1o).

### Flow cytometry and IHC analysis of dissociated tumours

For flow cytometry, tumours were collected as described above. Single-cell suspensions of dissociated tumours were stained for CAR (APC, HER2-Fc, R&D, 1:400), hCD19 (BUV 496, SJ25C1, BD, 1:100), CD11b (BUV 395, M1/70, BD, 1:100), F4/80 (BV605, BM8, BioLegend, 1:100), hCD45 (PerCP-Cy5.5, HI30, Invitrogen, 1:50), hCD3 (BUV 737, SK7, BD, 1:100), mCD45 (BUV 805, I3/2.3, BD, 1:100) and hCD47 (BV711, B6H12, BD, 1:100), and with Fixable Viability Dye (eFluor 780, Invitrogen, 1:2000) for 30 min in PBS + 2% FBS (FACS Buffer) before being analysed by flow cytometry.

For IHC analysis, tumours were collected as described above. Formalin-fixed, paraffin-embedded xenograft tumour sections were used. F4/80 (D2S9R, Cell Signaling Technology, 1:200) staining was performed manually, and hCD3 (SP7, Abcam, 1:100) and ARG1 (D4E3M, Cell Signaling Technology, 1:250) staining was performed using the Ventana Discovery platform. In brief, tissue sections were incubated in either 6 mM citrate buffer (F4/80, 1:200 dilution) or Tris EDTA buffer (CD3/ARG1, 1:100 and 1:250 dilution respectively; cell conditioning 1 standard) at 100 °C for 25 min (F4/80) or 95 °C for 1 h (CD3/ARG1) to retrieve antigenicity, followed by incubation with the respective primary antibody for 1 h. Bound primary antibodies were incubated with the respective secondary antibodies (goat anti-rabbit, polyclonal, F4/80: Vector Laboratories, undiluted or CD3/ARG1: Jackson ImmunoResearch, 1:500), followed by UltraMap HRP (Roche, F4/80) or ChromoMap DAB (Roche, CD3/ARG1) detection. For IHC analysis, tumour regions were identified on the basis of histology. F4/80, CD3 and ARG1 positivity were analysed for each tumour region. F4/80, CD3 and ARG1 IHC positivity scores were automatically quantified in the regions of interest using Aperio ImageScope software (v.12.3.2.8013). Regions of interest were randomly selected within the tumour to exclude macrophages present in the normal tissue around the tumour.

### Single-cell analysis of dissociated 143B tumours

Dissociated tumours from the 143B osteosarcoma model described above were sorted for live cells using a Live/Dead stain (Invitrogen) at the Stanford Shared FACS facility (the gating strategy is shown in Supplementary Fig. 2). scRNA-seq libraries were prepared using the Chromium Next GEM Single Cell 5’ v2 platform (10x GENOMICS). Libraries were sent to Novogene for sequencing on a NovoSeq S4 lane (PE150) with approximately 30,000 mean reads per cell. Reads were aligned and quantified using CellRanger (v.6.0; 10x GENOMICS) using the standard workflow, with the reference transcriptomes GRCh38 for human and mm10 for mouse. The CellRanger output was imported into R (v.4.2.2) using Seurat (v.4.2.0). The following filters were applied using the subset function to select for live cells: nFeature_RNA > 200 and nFeature_RNA < 5000; percent mitochondrial reads < 5%. After filtering, the eight biological samples ranged from 7,658–9,327 mean unique molecular identifiers per cell. The data matrix was normalized with NormalizeData and scaled with Seurat. Cell types were assigned using SingleR automated cell type recognition. Differential expression analysis, clustering and UMAP dimensionality reduction analysis were performed on the resulting data matrix using Seurat^46^. Pathway analysis was performed using Enrichr^47^, with the NCI-Nature 2016 gene set collection queried for human T cells and the KEGG Human Pathway collection queried with converted mouse gene IDs for mouse macrophages.

### Statistical analyses

The specific statistical tests used are indicated in the figure legends. Statistical analyses were performed using R (v.4.2.2), Excel (v.16.64; Microsoft) or Prism (v.9.3.1, GraphPad). For comparisons between two groups, statistical significance was assayed using two-tailed unpaired Student’s *t*-tests or Mann–Whitney *U*-tests. For comparison within in vivo studies and between grouped studies, two-way ANOVA combined with Tukey’s multiple-comparison test for post hoc analysis was performed. Significance for survival data was calculated using the log-rank Mantel–Cox test. Sample sizes were determined on the basis of the variability of tumour models used, determined by experience with well-established, previously published models^31,32,34,38^. Tumour-bearing animals were assigned to the treatment groups randomly to ensure an equal distribution of tumour sizes between groups. Data are represented as mean ± s.d. (in vitro studies) or mean ± s.e.m. (some in vivo studies). For all statistical analyses, *P* values are indicated in each figure panel.

### Reporting summary

Further information on research design is available in the Nature Portfolio Reporting Summary linked to this article.

## Online content

Any methods, additional references, Nature Portfolio reporting summaries, source data, extended data, supplementary information, acknowledgements, peer review information; details of author contributions and competing interests; and statements of data and code availability are available at 10.1038/s41586-024-07443-8.

### Supplementary information

Supplementary Information Supplementary Figs. 1–3 and Supplementary Notes, listing data duplications between figures.

Reporting Summary

Supplementary Tables Supplementary Tables 1–3.

Supplementary Video 1 Phagocytosis of a human T cell by a human macrophage. Time-lapse video of primary human CD19-28ζ CAR T cell phagocytosis by a primary human macrophage after treating with B6H12 in a three-dimensional collagen matrix. Representative images are shown in Extended Data Fig. 4b. CAR T cells (green) and macrophages (red) were labelled separately with lipophilic dyes. CAR T cells were also labelled with pH-sensitive pHrodo Red dye (blue) before B6H12 treatment. The video is representative across *n* = 2 experiments.

### Source data

Source Data Fig. 1

Source Data Fig. 2

Source Data Fig. 3

Source Data Fig. 4

Source Data Fig. 5

Source Data Extended Data Fig. 1

Source Data Extended Data Fig. 2

Source Data Extended Data Fig. 3

Source Data Extended Data Fig. 4

Source Data Extended Data Fig. 5

Source Data Extended Data Fig. 6

Source Data Extended Data Fig. 7

Source Data Extended Data Fig. 8

Source Data Extended Data Fig. 9

Source Data Extended Data Fig. 10

## Data Availability

All data associated with this paper are included in the Article and the Supplementary Information. The scRNA-seq dataset has been deposited at the NCBI Gene Expression Omnibus (GEO) under accession number GSE261475. Data used to generate scRNA-seq UMAP plots from patient data (Fig. 2d) were obtained from publicly available datasets using the GEO series accession numbers GSE168940 (ref. ^14^) and GSE186802 (ref. ^15^). For protein crystal structure modelling, the following publicly availably PDB files were used: 2JJS (hCD47–hSIRPa) and 5TZU (hCD47–B6H12). Source data are provided with this paper.
