# Supplementary material for: Engineered CD47 protects T cells for enhanced antitumour immunity
Source: Nature. 2024 May 15;630(8016):457–65. doi: 10.1038/s41586-024-07443-8 (PMC11168929; doi:10.1038/s41586-024-07443-8)
Supplement: Supplementary file 1 — Supplementary Figs. 1–3 and Supplementary Notes, listing data duplications between figures. [file 41586_2024_7443_MOESM1_ESM.pdf]

nature portfolio

https://doi.org/10.1038/s41586-024-07443-8

Supplementary information

Engineered CD47 protects T cells for enhanced antitumour immunity

In the format provided by the authors and unedited

Supplementary Fig. 1 | Method schematics for *in vivo* studies

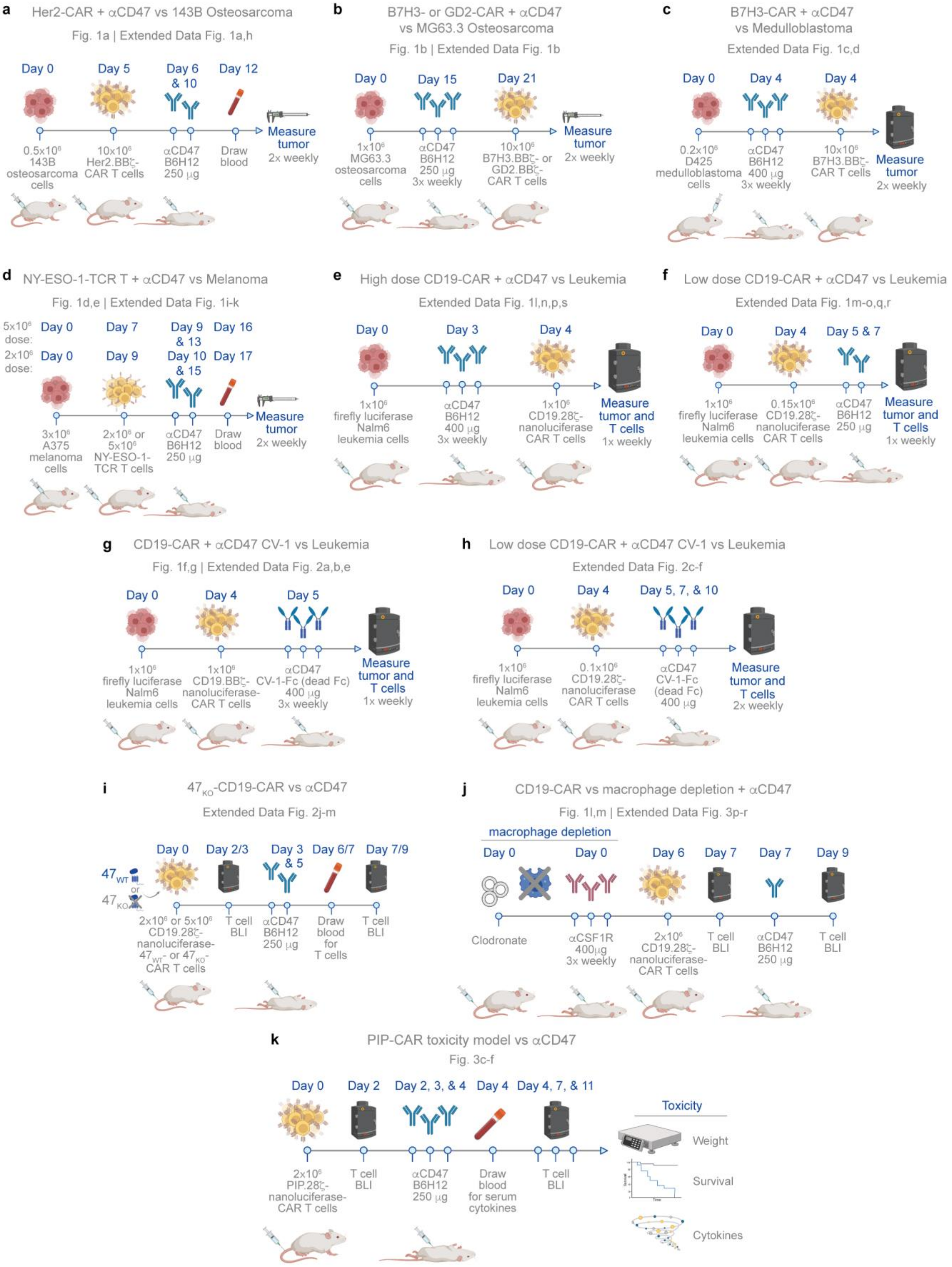

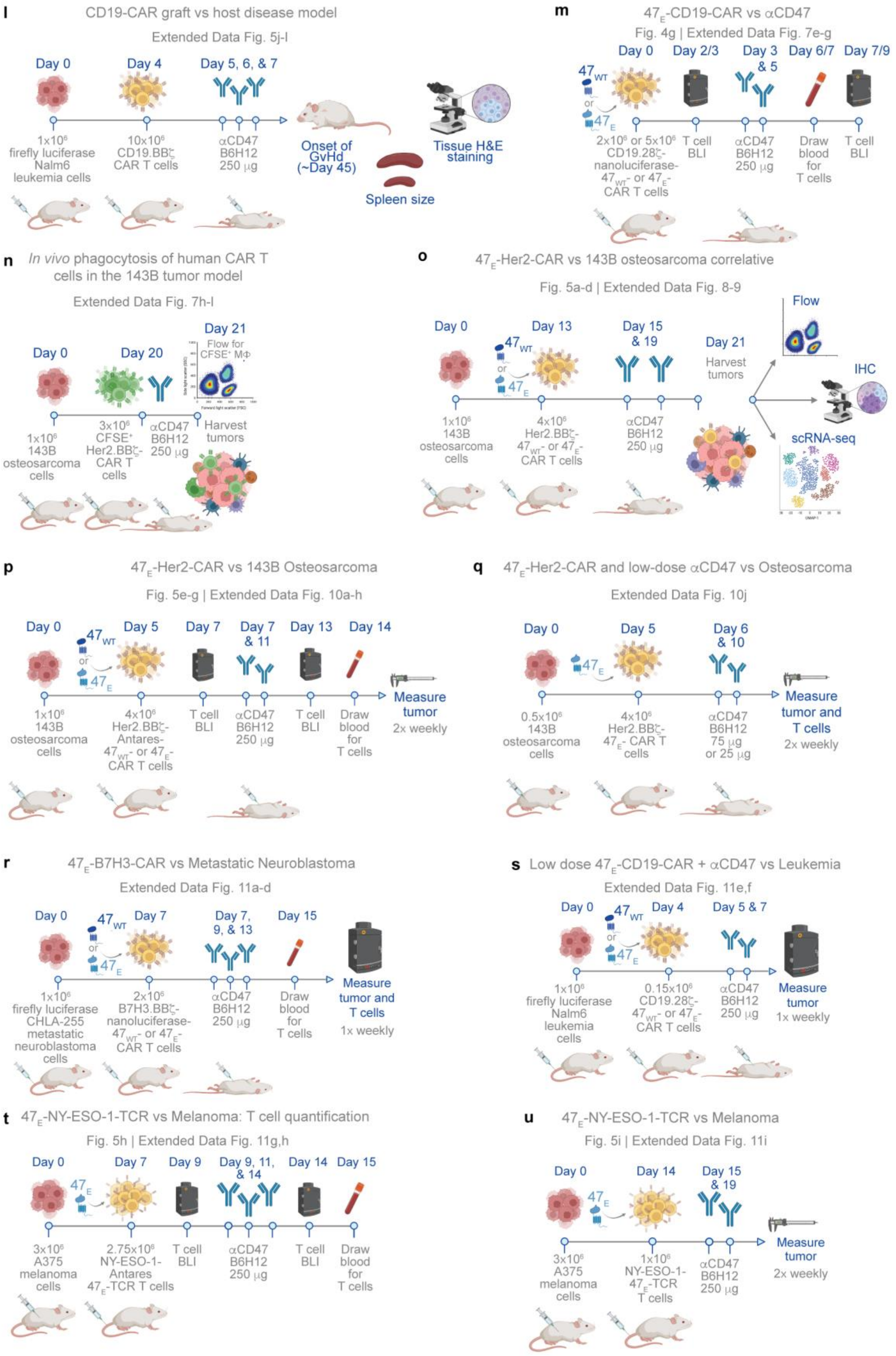

l
CD19-CAR graft vs host disease model
Extended Data Fig. 5j-l
Day 0
Day 4
Day 5, 6, & 7
1x10^6 firefly luciferase Nalm6 leukemia cells
10x10^6 CD19.BBζ CAR T cells
αCD47 B6H12 250 μg
Onset of GvHd (~Day 45)
Tissue H&E staining
Spleen size
m
47E-CD19-CAR vs αCD47
Fig. 4g | Extended Data Fig. 7e-g
Day 0
Day 2/3
Day 3 & 5
Day 6/7
Day 7/9
47WT
or
47E
2x10^6 or 5x10^6 CD19.28ζ-nanoluciferase-47WT- or 47E-CAR T cells
T cell BLI
αCD47 B6H12 250 μg
Draw blood for T cells
T cell BLI
n
In vivo phagocytosis of human CAR T cells in the 143B tumor model
Extended Data Fig. 7h-l
Day 0
Day 20
Day 21
Flow for CFSE+ MΦ
1x10^6 143B osteosarcoma cells
3x10^6 CFSE+ Her2.BBζ-CAR T cells
αCD47 B6H12 250 μg
Harvest tumors
o
47E-Her2-CAR vs 143B osteosarcoma correlative
Fig. 5a-d | Extended Data Fig. 8-9
Day 0
Day 13
Day 15 & 19
Day 21
Harvest tumors
47WT
or
47E
1x10^6 143B osteosarcoma cells
4x10^6 Her2.BBζ-47WT- or 47E-CAR T cells
αCD47 B6H12 250 μg
Flow
IHC
scRNA-seq
p
47E-Her2-CAR vs 143B Osteosarcoma
Fig. 5e-g | Extended Data Fig. 10a-h
Day 0
Day 5
Day 7
Day 7 & 11
Day 13
Day 14
47WT
or
47E
1x10^6 143B osteosarcoma cells
4x10^6 Her2.BBζ-Antares-47WT- or 47E-CAR T cells
T cell BLI
αCD47 B6H12 250 μg
T cell BLI
Draw blood for T cells
Measure tumor
2x weekly
q
47E-Her2-CAR and low-dose αCD47 vs Osteosarcoma
Extended Data Fig. 10j
Day 0
Day 5
Day 6 & 10
47E
0.5x10^6 143B osteosarcoma cells
4x10^6 Her2.BBζ-47E-CAR T cells
αCD47 B6H12 75 μg or 25 μg
Measure tumor and T cells
2x weekly
r
47E-B7H3-CAR vs Metastatic Neuroblastoma
Extended Data Fig. 11a-d
Day 0
Day 7
Day 7, 9, & 13
Day 15
47WT
or
47E
1x10^6 firefly luciferase CHLA-255 metastatic neuroblastoma cells
2x10^6 B7H3.BBζ-nanoluciferase-47WT- or 47E-CAR T cells
αCD47 B6H12 250 μg
Draw blood for T cells
Measure tumor and T cells
1x weekly
s
Low dose 47E-CD19-CAR + αCD47 vs Leukemia
Extended Data Fig. 11e,f
Day 0
Day 4
Day 5 & 7
47WT
or
47E
1x10^6 firefly luciferase Nalm6 leukemia cells
0.15x10^6 CD19.28ζ-47WT- or 47E-CAR T cells
αCD47 B6H12 250 μg
Measure tumor
1x weekly
t
47E-NY-ESO-1-TCR vs Melanoma: T cell quantification
Fig. 5h | Extended Data Fig. 11g,h
Day 0
Day 7
Day 9
Day 9, 11, & 14
Day 14
Day 15
47E
3x10^6 A375 melanoma cells
2.75x10^6 NY-ESO-1-Antares 47E-TCR T cells
T cell BLI
αCD47 B6H12 250 μg
T cell BLI
Draw blood for T cells
u
47E-NY-ESO-1-TCR vs Melanoma
Fig. 5i | Extended Data Fig. 11i
Day 0
Day 14
Day 15 & 19
47E
3x10^6 A375 melanoma cells
1x10^6 NY-ESO-1-47E-TCR T cells
αCD47 B6H12 250 μg
Measure tumor
2x weekly

Supplementary Fig. 1 | Method schematics for *in vivo* studies.

(a) 143B model treatment scheme. Mice engrafted orthotopically in the tibia periosteum with 0.5×10^6 143B were treated intravenously (IV) with 10×10^6 Her2.BBζ-CAR-T cells on day 5, and then intraperitoneally (IP) ± B6H12 twice on days 6 and 10 (250 µg/dose). Blood was drawn on day 12 to assess CAR-T cell expansion.

(b) MG63.3 model treatment scheme. Mice engrafted orthotopically in the tibia periosteum with 1×10^6 MG63.3 were treated IP ± B6H12 three times per week (400 µg/dose) starting on day 15, then IV with 10×10^6 B7H3.BBζ- or GD2.BBζ-CAR-T cells on day 21.

(c) D425 treatment scheme. Mice engrafted with 0.2×10^6 D425 cells in the cerebellum were treated ± B6H12 IP three times per week (400 µg/dose) starting on day 4. Mice were also treated IV with 10×10^6 CD19.BBζ- (non-tumor targeting control) or B7H3.BBζ- (tumor targeting) CAR-T cells on day 4.

(d) A375 model treatment scheme. Mice engrafted subcutaneously (SQ) with 3×10^6 A375 were treated IV with two different dosed of mock or NY-ESO-1-TCR-T cells. The 2×10^6 dose was administered on day 9 ± two doses of B6H12 (250 µg/dose; IP) on days 10 and 15. Blood was drawn on day 17 to assess T cell expansion. The 5×10^6 dose was administered on day 7 ± two doses of B6H12 (250 µg/dose; IP) on days 9 and 13. Blood was drawn on day 16 to assess T cell expansion.

(e) High-dose CAR-T – Nalm6 model treatment scheme. Mice engrafted IV with 1×10^6 Nalm6-fLuc cells were treated ± B6H12 (400 µg/dose; IP) three times per week starting on day 3. Mice were then treated IV with 1×10^6 mock or CD19.28ζ-nLuc-CAR-T cells on day 4. Mice were serially imaged by BLI for both tumor growth (fLuc signal) and T cell expansion (nLuc signal).

(f) Low-dose CAR-T – Nalm6 model treatment scheme. Mice engrafted IV with 1×10^6 Nalm6-fLuc cells were then treated IV with 0.15×10^6 mock or CD19.28ζ-nLuc-CAR-T cells on day 4. Mice were then treated ± B6H12 (250 µg/dose; IP) on days 5 and 7. Mice were serially imaged by BLI for both tumor growth (fLuc signal) and T cell expansion (nLuc signal).

(g) Nalm6 with high-dose CAR-T and CV-1 treatment scheme. Mice engrafted IV with 1×10^6 Nalm6-fLuc were treated IV with 1×10^6 CD19.BBζ-nLuc-CAR-T cells on day 4, ± CV-1 (dead Fc; 400 µg/dose; IP) three times per week starting on day 5.

(h) Nalm6 with low-dose CAR-T and CV-1 treatment scheme. Mice engrafted IV with 1×10^6 Nalm6-fLuc were treated IV with 0.1×10^6 CD19.28ζ-nLuc-CAR-T cells on day 4, ± CV-1 (dead Fc; 400 µg/dose; IP) three times on days 5, 7 and 10.

(i) 47_{KO}- vs 47_{WT}-CAR-T cell depletion scheme. Non-tumor bearing mice were treated IV with two different doses of 47_{KO}-CD19.28ζ-nLuc-CAR-T cells, with (47_{WT}) or without (47_{KO}) CD47 exogenous expression and treated twice ± B6H12 (250 µg/dose; IP) on days 3 and 5. Mice dosed with 2×10^6 CAR-T cells were imaged by BLI on day 2 before and on day 9 after αCD47 treatment, and had blood drawn on day 7. Mice dosed with 5×10^6 CAR-T cells were imaged by BLI on day 3 before and on day 7 after αCD47 treatment, and had blood drawn on day 6.

(j) Macrophage depletion + CAR-T treatment scheme. Non-tumor bearing mice were treated with clodronate (200 µL; IV) on day 0 and αCSF1R (400 µg/dose; IP) three times per week starting on day 0 to deplete macrophages. Mice were then treated IV with 2×10^6 CD19.28ζ-nLuc-CAR-T cells on day 6, followed by a single 250 µg IP dose of B6H12 on day 7. Mice were imaged by BLI before (day 7) and after (day 9) αCD47 treatment.

(k) PIP-CAR toxicity model safety-switch scheme. Mice were treated IV with 2×10^6 CD19.28ζ-nLuc- or PIP.28ζ-nLuc-CAR-T cells ± B6H12 treatment (250 µg daily; IP) three times, two days later. Mice were

imaged by BLI before (day 2) and after (days 4, 7, and 11) αCD47 treatment. Blood was collected on day 4 for cytokine analysis.

(l) Scheme of CAR-T GvHD model. Mice engrafted IV with 1×10^6 Nalm6-fLuc were treated IV with a high dose of 10×10^6 CD19.BBζ-CAR T cells on day 4 ± three doses of B6H12 (250 µg/dose; IP) on days 5, 6, and 7. Mice were monitored for onset of GvHD around day 45.

(m) 47_E- vs 47_{WT}-CAR-T cell depletion scheme. Non-tumor bearing mice were treated IV with 5×10^6 47_{WT}- or 47_E-CD19.28ζ-nLuc-CAR-T cells (with endogenous 47_{KO}) and treated twice ± B6H12 (250 µg/dose; IP) on days 3 and 5. Mice dosed with 2×10^6 CAR-T cells were imaged by BLI on day 2 before and on day 9 after αCD47 treatment, and had blood drawn on day 7. Mice dosed with 5×10^6 CAR-T cells were imaged by BLI on day 3 before and on day 7 after αCD47 treatment, and had blood drawn on day 6.

(n) Scheme of *in vivo* CAR-T phagocytosis model. Mice were engrafted orthotopically in the tibia periosteum with 1×10^6 143B cells and treated intratumorally (IT) with 3×10^6 Her2.BBζ-CAR-T cells on day 20. Mice were co-treated ± B6H12 (250 µg/dose; IP) on day 20. Tumors were excised on day 21 and then analyzed via flow cytometry.

(o) Scheme of correlative mechanistic study in 143B osteosarcoma. Mice were engrafted orthotopically in the tibia periosteum with 1×10^6 143B-CD19 cells and treated IV with no T cells, or 4×10^6 mock, 47_{WT}- or 47_E-Her2.BBζ-CAR-T cells (with endogenous 47_{KO}) on day 13. Mice were then treated ± two doses of B6H12 (250 µg/dose; IP) on days 15 and 19. Tumors were excised on day 21 and then analyzed via flow cytometry, IHC, and scRNA-seq.

(p) 47_E-CAR-T – 143B treatment scheme. Mice were engrafted orthotopically in the tibia periosteum with 1×10^6 143B-CD19 cells and treated IV with 4×10^6 mock-Antares, or 47_{WT}- or 47_E-Her2.BBζ-Antares-CAR-T cells (with endogenous 47_{KO}) on day 5. Mice were then treated ± two doses of B6H12 (250 µg/dose; IP) on days 7 and 11. Mice were imaged by BLI before (day 7) and after (day 13) αCD47 treatment, and had blood drawn on day 14.

(q) Low dose αCD47 + 47_E-CAR-T – 143B treatment scheme. Mice were engrafted orthotopically in the tibia periosteum with 0.5×10^6 143B-CD19 cells and treated IV with 4×10^6 mock or 47_E-Her2.BBζ-CAR-T cells (with endogenous 47_{KO}) on day 5. Mice were then treated ± two doses of B6H12 (75 µg [~3 mg/kg] or 25 µg [~1 mg/kg] per dose; IP) on days 6 and 10.

(r) 47_E-CAR-T – CHLA-255 treatment scheme. Mice were engrafted IV with 1×10^6 CHLA-255-fLuc cells and treated IV with 2×10^6 mock-nLuc, 47_{WT}- or 47_E-B7H3.BBζ-nLuc-CAR-T cells (with endogenous 47_{KO}) on day 7. Mice were then treated ± three doses of B6H12 (250 µg/dose; IP) on days 7, 9 and 13. T cells were imaged by BLI on day 14 and blood was collected on day 15.

(s) 47_E-CAR-T – Nalm6 treatment scheme. Mice engrafted IV with 1×10^6 Nalm6-fLuc cells were then treated IV with 0.15×10^6 mock or 47_{WT}- or 47_E-CD19.28ζ-CAR-T cells on day 4. Mice were then treated ± B6H12 (250 µg/dose; IP) on days 5 and 7. Mice were serially imaged by BLI for tumor growth.

(t) Schematic of quantification of T cells in the A375 – NY-ESO-1 model. Mice engrafted SQ with 3×10^6 A375 were treated IV with 2.75×10^6 mock-Antares or 47_E-NY-ESO-1-Antares-TCR-T cells (with endogenous 47_{KO}) on day 7 ± three doses of B6H12 (250 µg/dose; IP) on days 9, 11, and 14. Mice were imaged by BLI before (day 9) and after (day 14) αCD47 treatment. Blood was collected on day 15.

(u) 47_E-TCR-T – A375 treatment scheme. Mice were engrafted SQ with 3×10^6 A375 cells and treated IV with 1×10^6 mock-Antares or 47_E-NY-ESO-1-Antares-TCR T cells (with endogenous 47_{KO}) on day 14. Mice were then treated ± two doses of B6H12 (250 µg/dose; IP) on days 15 and 19.

Supplementary Fig. 2 | Representative Flow Cytometry Gating Strategies

Representative gating strategy to identify human T cells from blood

Used for: Fig. 1c, 1e, 1g, 1k, 4h, 5f, and 5h | Extended Data Fig. 1e, 1f, 1g, 1h, 1k, 2j, 2m, 7g, 10a, 10b, 10c, 10i, 11a ,and 11h

Representative data from Fig. 1e

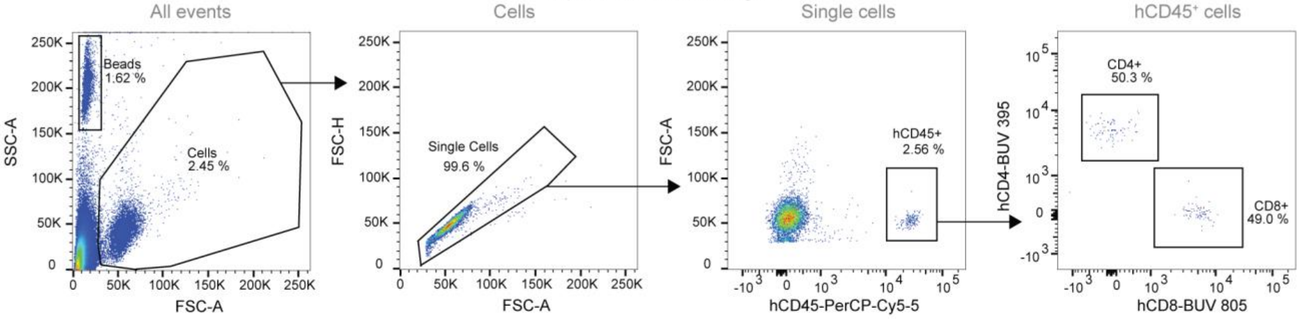

Representative gating strategy to identify human T cells from dissociated tumors and spleens

Used for: Fig. 1c | Extended Data Fig. 1e, 1s

Representative data from Fig. 1c (tumor)

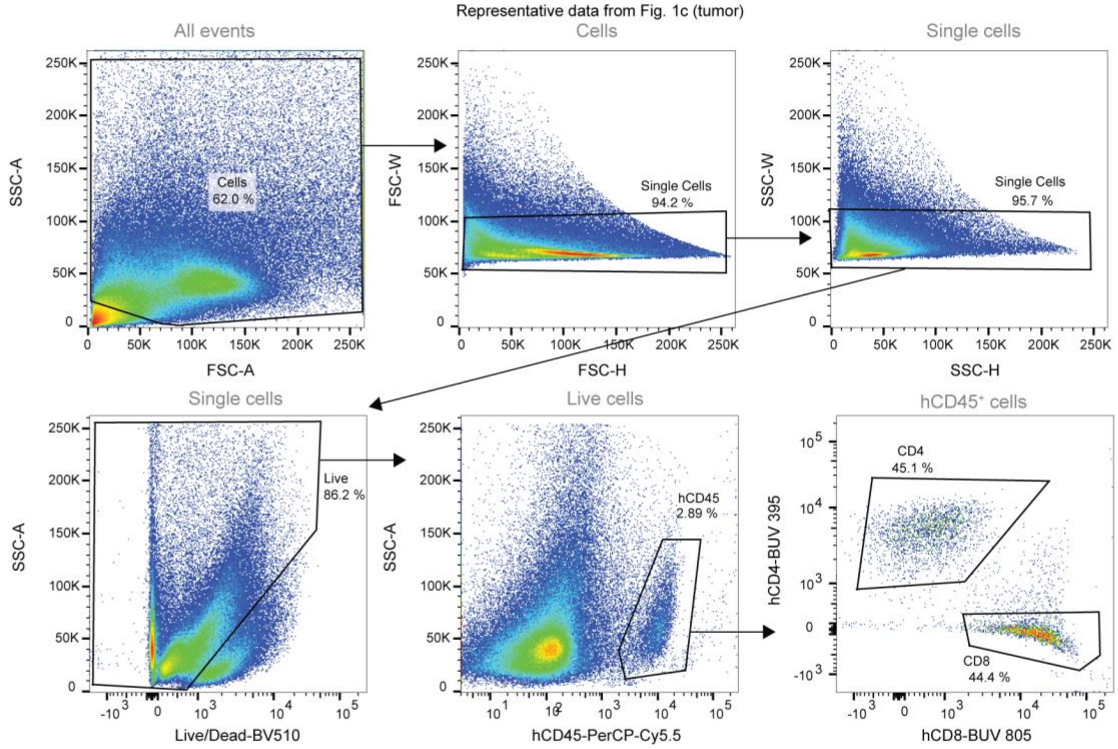

Representative gating strategy to quantify protein expression on T cells and Jurkats

Used for: Fig. 2b, 4e | Extended Data Fig. 3g-l, 3m, 4g, 4h, 5b, 7a, 7b

Representative data from Fig. 4e

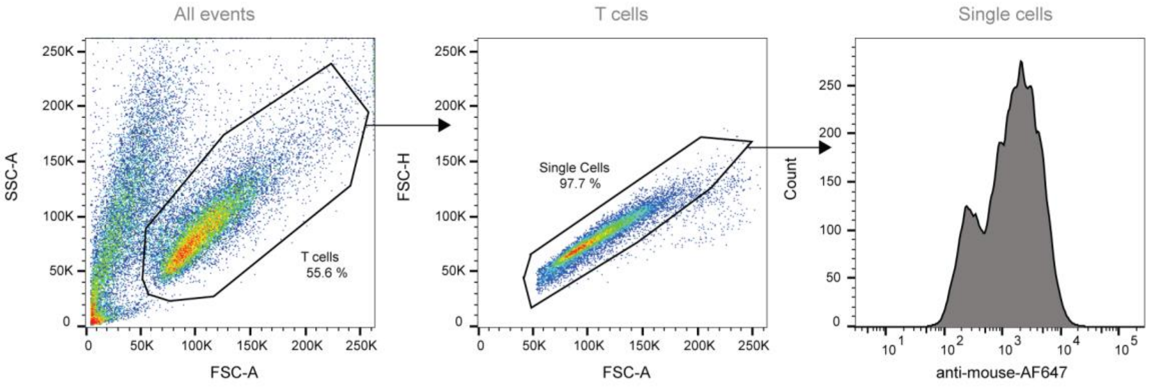

Representative gating strategy for phagocytosis assays

Used for: Fig. 2a and 4f | Extended Data Fig. 4a and 7c

Representative data from Extended Data Fig. 7c

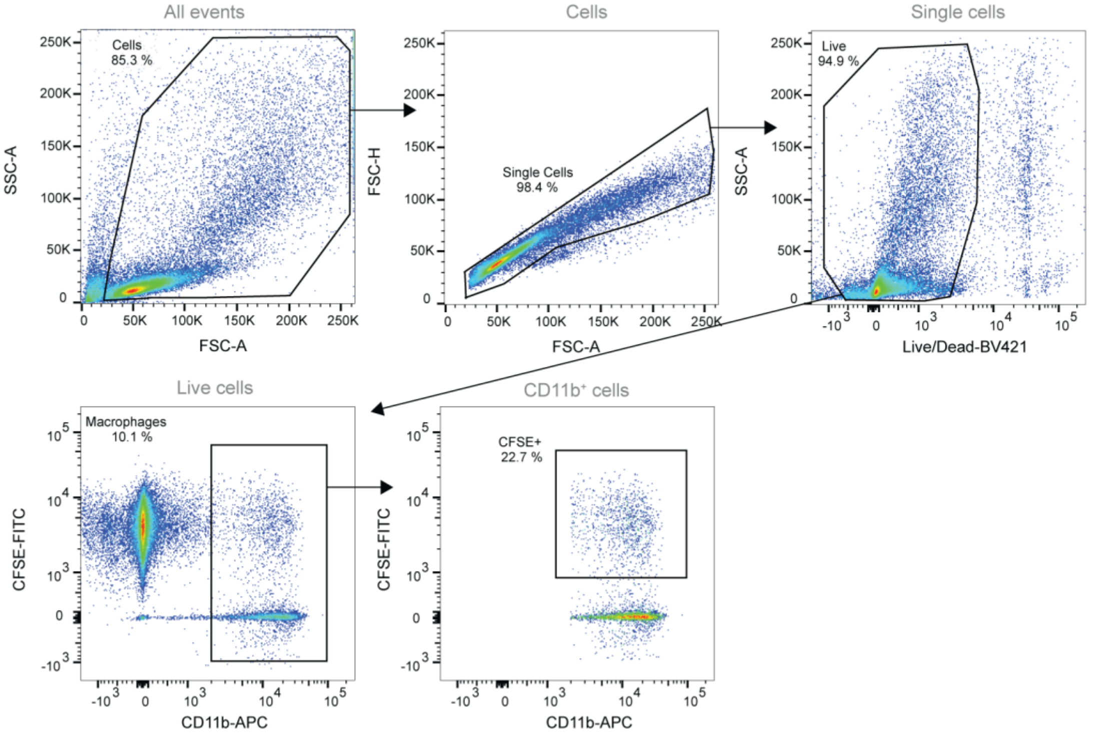

Representative gating strategy for assessing macrophage depletion after peritoneal lavage

Used for: Extended Data Fig. 3p and 3q

Representative data from Extended Data Fig. 3q

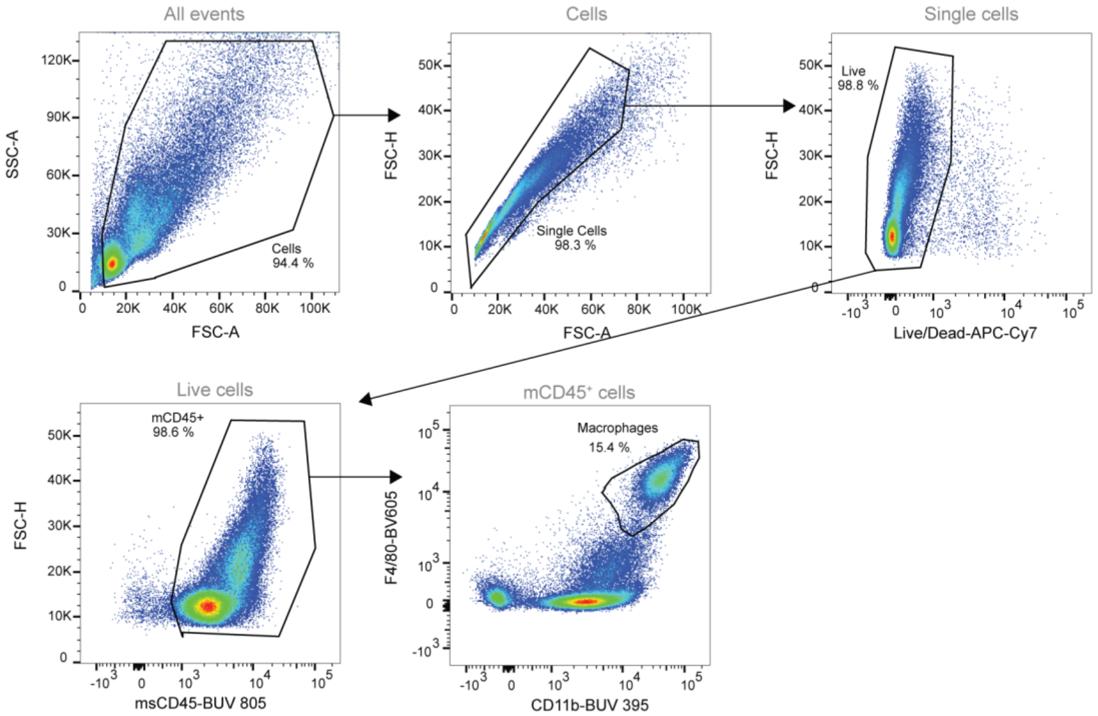

Representative gating strategy for quantifying CD47 expression using QuantiBrite

Used for: Extended Data Fig. 4c, 4d, 4e and 4f

Representative data from Extended Data Fig. 4f

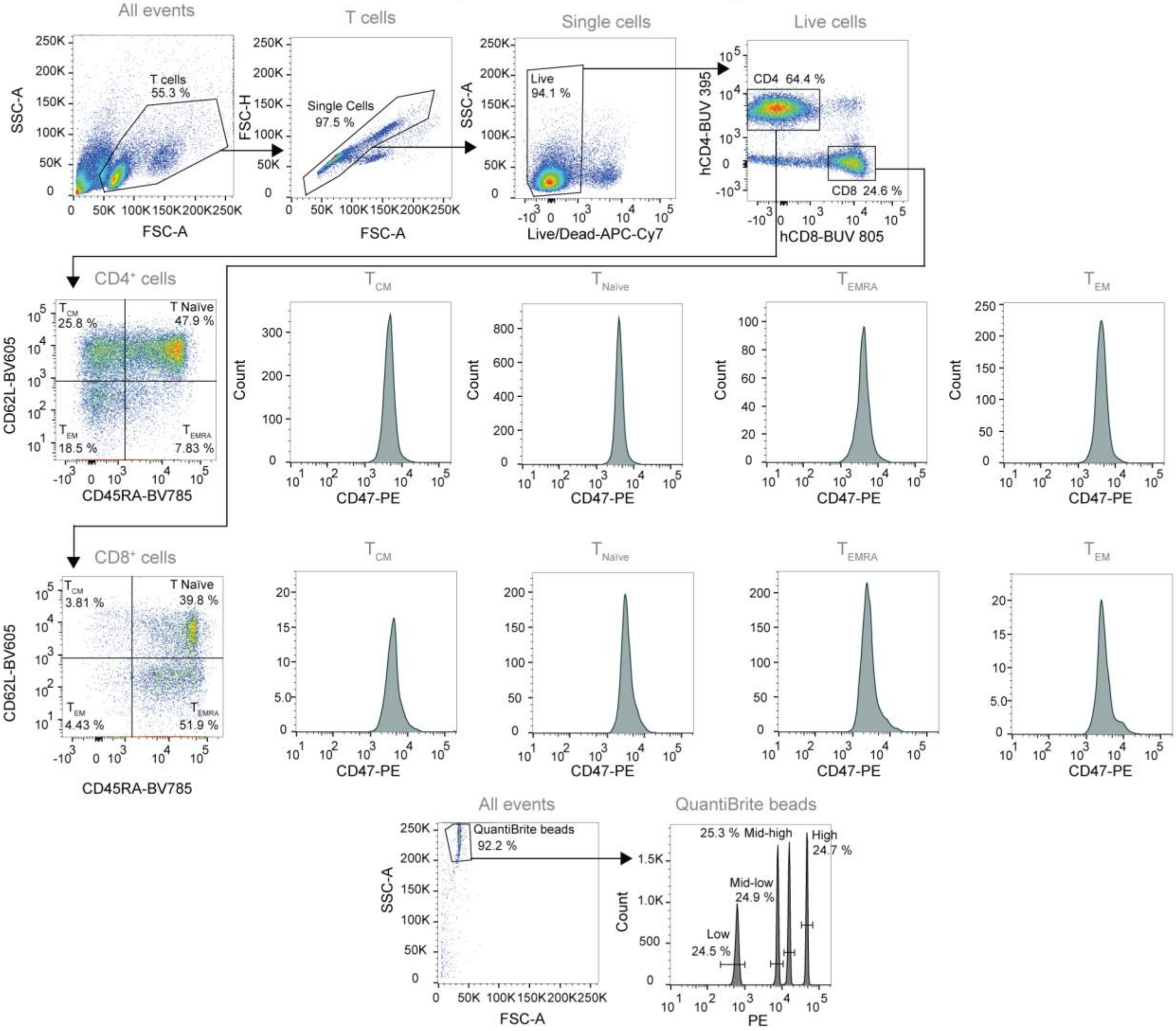

Representative gating strategy for yeast surface display binding and sorting

Used for: Fig. 4b and 4d | Extended Data Fig. 6c, 6d, 6e, 6f, 6g, 6h, 6i, 6k and 6n

Representative data from Extended Data Fig. 6c

Specific sorting gates for CD47 library on single cell populations shown in Extended Data Fig. 6g

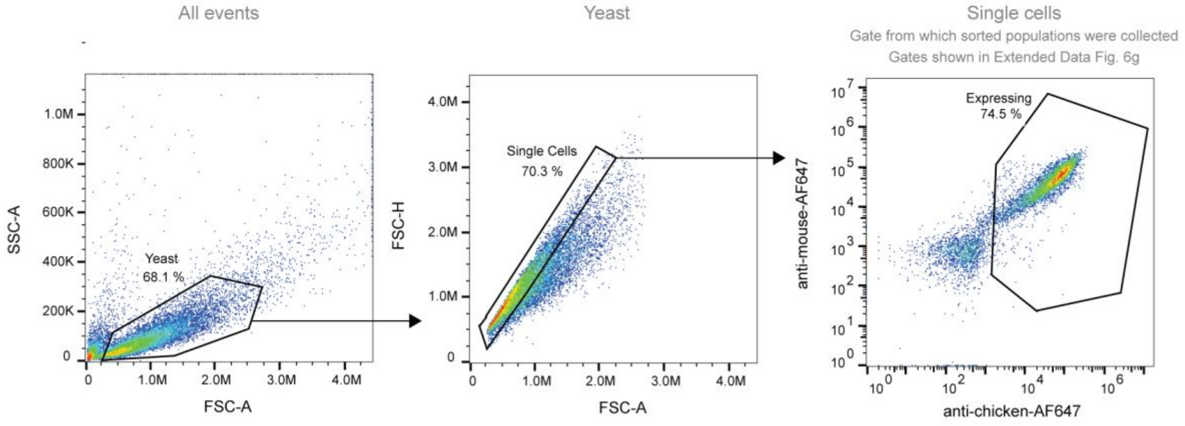

Representative gating strategy for quantifying T cells and macrophages in 143B tumor dissociations

Used for: Fig. 5a and Extended Data Fig. 8a

Representative data from Fig. 5a

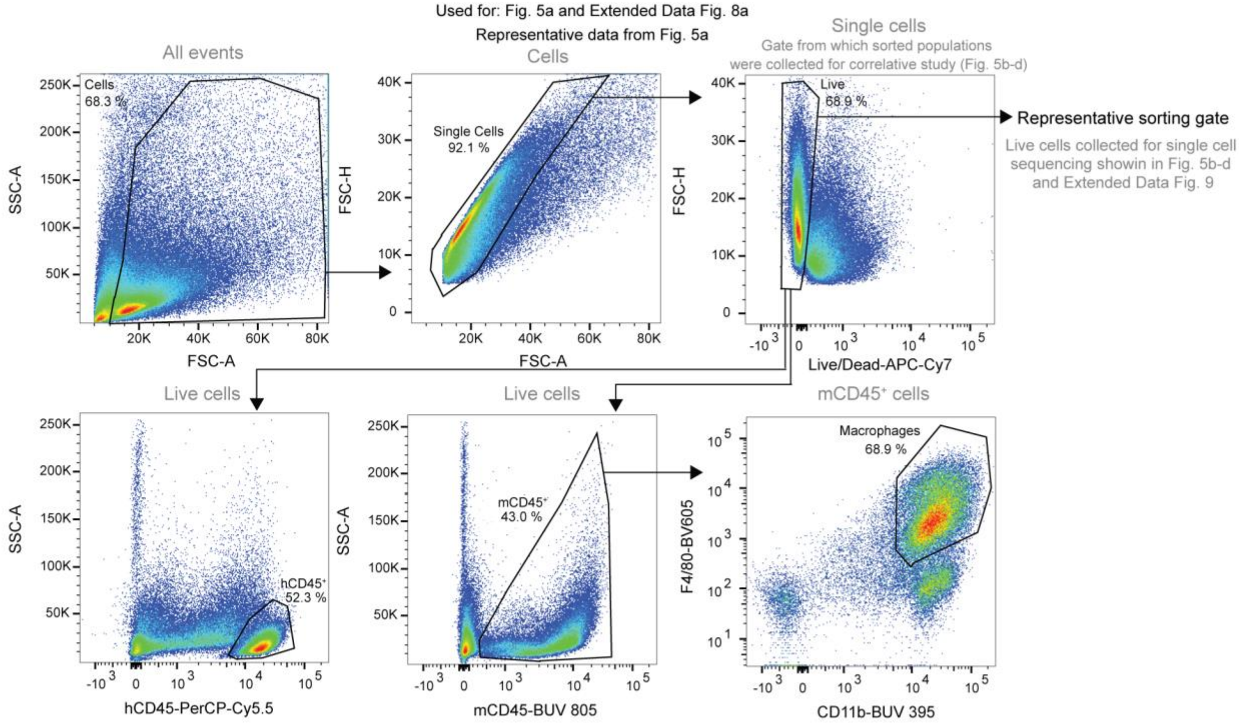

Representative gating strategy for T cells and macrophage identification in the *in vivo* phagocytosis assay

Used for: Extended Data Fig. 7h, 7i, 7j, 7k, and 7l

Representative data from Extended Data Fig. 7h

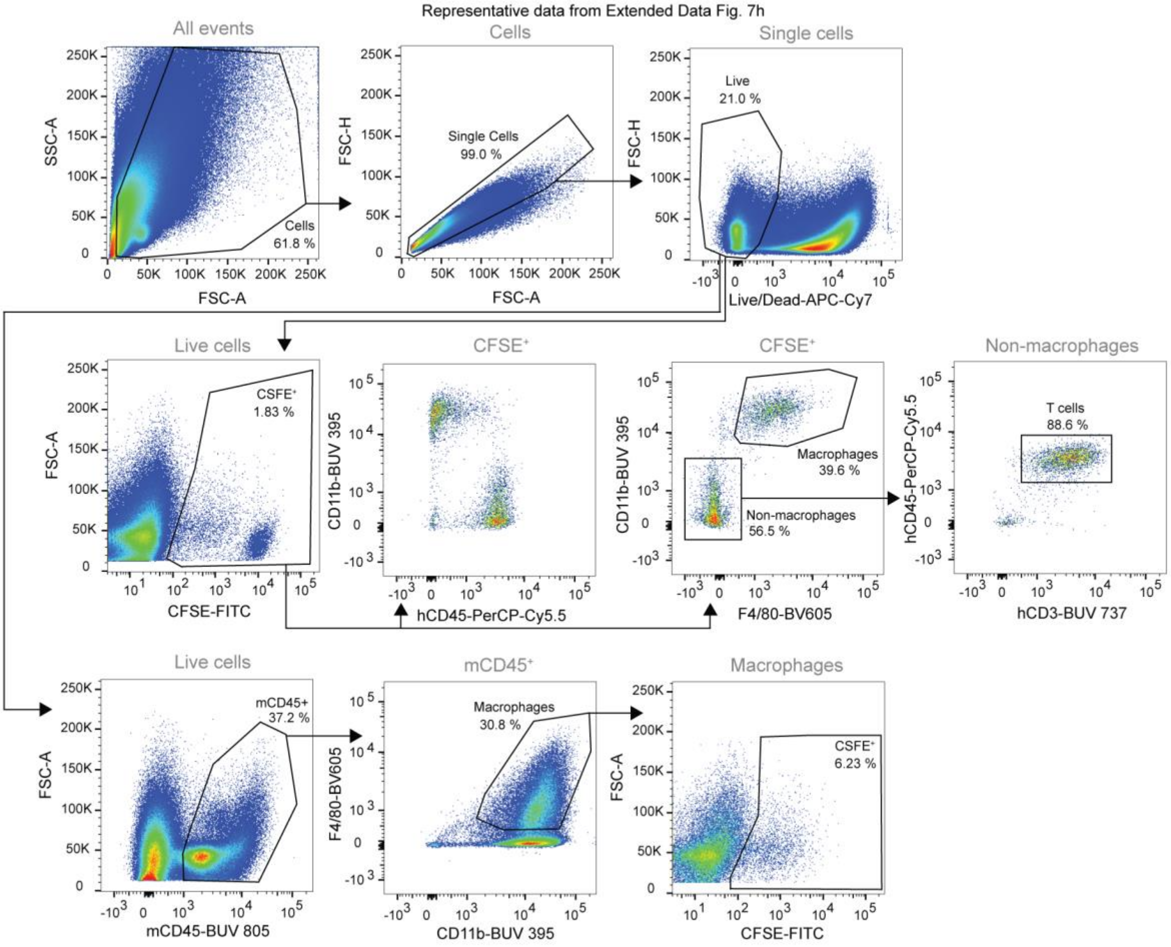

Supplementary Fig. 3 | Raw images of patient CSF sample shown in Fig. 2c

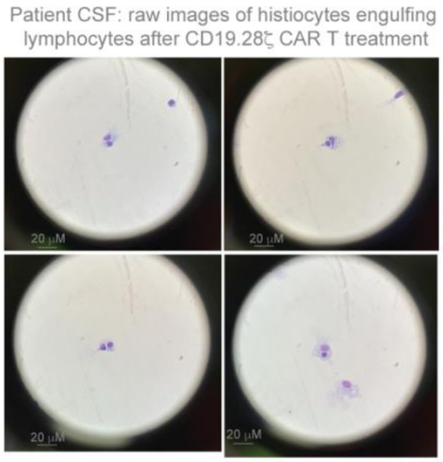

Supplementary Fig. 3 | Microscope images of histiocytes engulfing lymphocytes collected from the CSF of a CD19.28ζ-CAR T treated large B cell lymphoma (LBCL) patient and stained with hematoxylin and eosin (1000x magnification). Fig. 2c depicts enlargements of these images, kept in the same orientation of images.

Supplementary Notes

Data duplication between figures:

- Fig. 1h: Mock and 47_{WT}-CD19.28ζ are shared conditions duplicated in Extended Data Fig. 1m (mock) and Extended Data Fig. 11e (mock and 47_{WT}-CD19.28ζ).
- Fig. 1j: Mock and 47_{OE}-mock are shared conditions duplicated in Extended Data Fig. 3o.
- Fig. 4g: 47_{WT} is a shared condition duplicated in T cell depletion in Extended Data Fig. 2m.
- Fig. 5a: IHC data is also represented in Extended Data Fig. 8d.
- Extended Data Fig. 1m: Mock and mock + B6H12 are shared conditions duplicated in Fig. 1h (mock) and Extended Data Fig. 11e (mock and mock + B6H12).
- Extended Data Fig. 1n: Mock treatment is a shared condition, duplicated in Extended Data Fig. 2h.
- Extended Data Fig. 1o: Mock and mock + B6H12 are shared conditions duplicated in Extended Data Fig. 2i (mock) and Extended Data Fig. 11f (mock and mock + B6H12).
- Extended Data Fig. 2h: Mock treatment is a shared condition, with the image from day 17 duplicated in Extended Data Fig. 1n.
- Extended Data Fig. 2i: Mock and 47_{WT}-CD19.28ζ are shared conditions duplicated in Extended Data Fig. 1o (mock) and Extended Data Fig. 11f (mock and 47_{WT}-CD19.28ζ).
- Extended Data Fig. 2k: 47_{WT} is a shared condition duplicated in T cell depletion in Extended Data Fig. 7e.
- Extended Data Fig. 2l: 47_{WT} is a shared condition duplicated in T cell depletion in Extended Data Fig. 7f.
- Extended Data Fig. 2m: 47_{WT} is a shared condition duplicated in T cell depletion in Fig. 4g and Extended Data Figure 7g.
- Extended Data Fig. 3o: Mock and 47_{OE}-mock are a shared conditions duplicated in Fig. 1j.
- Extended Data Fig. 7e: 47_{WT} is a shared condition duplicated in T cell depletion in Extended Data Fig. 2k.
- Extended Data Fig. 7f: 47_{WT} is a shared condition duplicated in T cell depletion in Extended Data Fig. 2l.
- Extended Data Fig. 7g: 47_{WT} is a shared condition duplicated in T cell depletion in Extended Data Fig. 2m.
- Extended Data Fig. 8b: Data is also represented in Extended Data Fig. 8d.
- Extended Data Fig. 8d: hCD3 is also represented in Extended Data Fig. 8b. F4/80 is also represented in Fig. 5a.
- Extended Data Fig. 11e: Mock, mock + B6H12, and 47_{WT}-CD19.28ζ are shared conditions duplicated in Fig. 1h (mock and 47_{WT}-CD19.28ζ) and Extended Data Fig. 1m (mock and mock + B6H12).
- Extended Data Fig. 11f: Mock, mock + B6H12, and 47_{WT}-CD19.28ζ are shared conditions duplicated in Extended Data Fig. 1o (mock and mock + B6H12) and Extended Data Fig. 2i (mock and 47_{WT}-CD19.28ζ).
