# Supplementary material for: Engineered CD47 protects T cells for enhanced antitumour immunity
Source: Nature. 2024 May 15;630(8016):457–65. doi: 10.1038/s41586-024-07443-8 (PMC11168929; doi:10.1038/s41586-024-07443-8)
Supplement: Supplementary file 2 — Reporting Summary [file 41586_2024_7443_MOESM2_ESM.pdf]

nature portfolio

Corresponding author(s): Crystal Mackall

Last updated by author(s): Mar 26, 2024

Reporting Summary

Nature Portfolio wishes to improve the reproducibility of the work that we publish. This form provides structure for consistency and transparency in reporting. For further information on Nature Portfolio policies, see our Editorial Policies and the Editorial Policy Checklist.

Statistics

For all statistical analyses, confirm that the following items are present in the figure legend, table legend, main text, or Methods section.

n/a	Confirmed	
☐	☒	The exact sample size (*n*) for each experimental group/condition, given as a discrete number and unit of measurement
☐	☒	A statement on whether measurements were taken from distinct samples or whether the same sample was measured repeatedly
☐	☒	The statistical test(s) used AND whether they are one- or two-sided<br>*Only common tests should be described solely by name; describe more complex techniques in the Methods section.*
☐	☒	A description of all covariates tested
☐	☒	A description of any assumptions or corrections, such as tests of normality and adjustment for multiple comparisons
☐	☒	A full description of the statistical parameters including central tendency (e.g. means) or other basic estimates (e.g. regression coefficient) AND variation (e.g. standard deviation) or associated estimates of uncertainty (e.g. confidence intervals)
☐	☒	For null hypothesis testing, the test statistic (e.g. *F*, *t*, *r*) with confidence intervals, effect sizes, degrees of freedom and *P* value noted<br>*Give P values as exact values whenever suitable.*
☒	☐	For Bayesian analysis, information on the choice of priors and Markov chain Monte Carlo settings
☒	☐	For hierarchical and complex designs, identification of the appropriate level for tests and full reporting of outcomes
☒	☐	Estimates of effect sizes (e.g. Cohen's *d*, Pearson's *r*), indicating how they were calculated

Our web collection on statistics for biologists contains articles on many of the points above.

Software and code

Policy information about availability of computer code

Data collection	FACSDiva ver 8.0.1 (BD Biosciences): Flow cytometry data acquisition<br>BD Accuri C6 software ver 1.0.264.21 (BD Biosciences): Flow cytometry data acquisition<br>IncuCyte S3 ver 2019B Rev2 Software (Sartorius): Cytotoxicity data acquisition<br>LivingImage ver 4.7.3 (Perkin Elmer): In Vivo Bioluminescence data acquisition<br>Aura ver 4.0.7 (Spectral Instruments Imaging): In Vivo Bioluminescence data acquisition<br>SH800S cell sorter software ver 2.1.5 (SONY): Cell sorting data acquisition<br>Gen5 ver 2.00.18 (BioTek): ELISA quantification<br>Imaris ver 10.0 (Oxford Instruments): confocal microscopy<br>cellSens ver 3.2 (Olympus Life Science): histology microscopy

Data analysis

FlowJo ver 10.8.1 (BD): Flow cytometry data analysis
Excel ver 16.64 (Microsoft): Analysis of bulk data
Prism ver 9.5.1 (GraphPad): Generation of graphs and statistical analysis
SnapGene ver 6.0.2 (Dotmatics): DNA vector design and molecular cloning
Aura ver 4.0.7 (Spectral Instruments Imaging): In Vivo Bioluminescence data quantification
Imaris ver 10.0 (Oxford Instruments): confocal microscopy
PyMol ver 2.5.8 (Schrödinger, LLC): protein structure modeling
Aperio ImageScope ver 12.3.2.8013: IHC positivity score analysis
CellRanger ver 6.0: demultiplexing and alignment of sequencing reads to host reference genomes
Seurat ver 4.2.0: cell type identification, differential gene expression analyses, and visualizations
R ver 4.2.2: statistical and scRNA-seq data analysis

For manuscripts utilizing custom algorithms or software that are central to the research but not yet described in published literature, software must be made available to editors and reviewers. We strongly encourage code deposition in a community repository (e.g. GitHub). See the Nature Portfolio guidelines for submitting code & software for further information.

Data

Policy information about availability of data

All manuscripts must include a data availability statement. This statement should provide the following information, where applicable:

- Accession codes, unique identifiers, or web links for publicly available datasets
- A description of any restrictions on data availability
- For clinical datasets or third party data, please ensure that the statement adheres to our policy

All data associated with this paper are included in the manuscript and the supplementary materials. All raw data are provided in the source data files. The scRNA-seq dataset has been deposited in the NCBI Gene Expression Omnibus (GEO) and is accessible through the GEO series accession number GSE261475. Data used to generate scRNA-seq UMAP plots from patient data (Fig. 2d) were obtained from publicly available data sets using the GEO series accession numbers GSE168940 (Good Z., et. al., Nature Medicine, 2022) and GSE186802 (Majzner, R.G., et. al., Nature, 2022). For protein crystal structure modeling, the following publicly availably PDB files were used: 2JJS (hCD47 - hSIRPa) and 5TZU (hCD47 - B6H12).

Human research participants

Policy information about studies involving human research participants and Sex and Gender in Research.

Reporting on sex and gender

Sex information for patients can be found in previous publications where the datasets we re-analyzed were originally published: (Good, Z. et. al. Nature Medicine, 2022 and Majzner, R.G., et. al., Nature, 2022). Gender information was not collected. Human T cells were isolated from buffy coats or leuokopaks from anonymous healthy donors (male and female; gender information was not collected) purchased from the Stanford Blood Center or STEMCELL Technologies, respectively.

Population characteristics

Patient characteristics can be found in previous publications where the datasets we re-analyzed were originally published: (Good, Z. et. al. Nature Medicine, 2022 and Majzner, R.G., et. al., Nature, 2022). Human T cells were isolated from buffy coats or leuokopaks from anonymous healthy donors (male and female, under 45) purchased from the Stanford Blood Center or STEMCELL Technologies, respectively.

Recruitment

Patient recruitment information can be found in previous publications where the datasets we re-analyzed were originally published: (Good, Z. et. al. Nature Medicine, 2022 and Majzner, R.G., et. al., Nature, 2022). Written informed consent was obtained from all healthy T cell donors by the Stanford Blood Center and STEMCELL Technologies.

Ethics oversight

Both studies we re-analyzed data from (Good, Z. et. al. Nature Medicine, 2022 and Majzner, R.G., et. al., Nature, 2022) were approved by the Stanford University IRB. Ethical approval pertaining to T cell donors was obtained by the Stanford Blood Center and STEMCELL Technologies. Blood cells used were exempt from IRB approval as there was no identifiable information provided about blood donors.

Note that full information on the approval of the study protocol must also be provided in the manuscript.

Field-specific reporting

Please select the one below that is the best fit for your research. If you are not sure, read the appropriate sections before making your selection.

☒ Life sciences ☐ Behavioural & social sciences ☐ Ecological, evolutionary & environmental sciences

For a reference copy of the document with all sections, see nature.com/documents/nr-reporting-summary-flat.pdf

Life sciences study design

All studies must disclose on these points even when the disclosure is negative.

Sample size

No sample size calculations were performed. Group sizes were determined by experience with well-established, previously published models

Sample size	(1, 2)<br><br>1. Labanieh, L., et al., Enhanced safety and efficacy of protease-regulated CAR-T cell receptors. Cell, 2022. 185(10): p. 1745-1763.e22.<br>2. Theruvath, J., et al., Anti-GD2 synergizes with CD47 blockade to mediate tumor eradication. Nature Medicine 2022 28:2, 2022. 28(2): p. 333-344.
Data exclusions	In the experiments described in Figure 1l,m, one mouse in the macrophage depletion group died prematurely and was excluded from the study. In the IHC and flow analysis described in Figure 5a and Extended Data Figure 8a-e some data were excluded due to lack of detectable tumor after dissociation, notably in the 47E-CAR treated group.
Replication	At least 2 different donor T cells were used for every experiment with primary T cells (save for those noted below) and each experiment was performed independently. For experiments where one representative donor was shown, data were representative of all donors. Experiments with Jurkat cells were performed independently at least 3 times and with different donor derived macrophages. Experiments with yeast were performed with three different clones and were performed independently. All attempts at replication were successful. Only one T cell donor was used for the CHLA-255 metastatic model described in Extended Data Figure 11a-d, the 143B correlative study described in Figure 5a-d and Extended Data Figures 8 and 9, the in vivo phagocytosis model described in Extended Data Figure 7h-l, and the low-dose CD19 CAR-Nalm6 model described in Extended Data Figure 1, Figure 1h,i, and Extended Data Figure 11e,f. A single donor was used in these cases due to practical experimental limitations (such as for single cell RNA sequencing experiments), similar prior experiments with different donors and different doses, but similar results (such as Nalm6 experiments), or confirmation of replication though smaller scale prior pilot studies (such as for CHLA-255, in vivo phagocytosis, and 143B tumor dissociation experiments).
Randomization	For in vivo experiments, cages of mice that were previously engrafted with tumor were randomly assigned CAR T cell and anti-CD47 conditions for infusion, ensuring roughly equal distributions of tumor size between groups prior to treatment. Experiments with primary human T cells were performed using T cells isolated from healthy T cell donors (under age 45) chosen at random. Transformed yeast clones were chosen at random for each condition. Otherwise, all other experiments were not randomized, but instead included controls to account for covariates (such as mock transduced T cells derived from the same donor as CAR T cells or yeast cells stained with only secondary antibodies).
Blinding	In vivo tumor engraftment and T cell infusion were performed by a technician who was blinded to treatments and expected outcomes. Otherwise, fully informed data analysis was performed. Fully blinded experiments were not possible due to personnel availability to accommodate such situations.

Reporting for specific materials, systems and methods

We require information from authors about some types of materials, experimental systems and methods used in many studies. Here, indicate whether each material, system or method listed is relevant to your study. If you are not sure if a list item applies to your research, read the appropriate section before selecting a response.

Materials & experimental systems

n/a	Involved in the study	
☐	☒	Antibodies
☐	☒	Eukaryotic cell lines
☒	☐	Palaeontology and archaeology
☐	☒	Animals and other organisms
☒	☐	Clinical data
☒	☐	Dual use research of concern

Methods

n/a	Involved in the study	
☒	☐	ChIP-seq
☐	☒	Flow cytometry
☒	☐	MRI-based neuroimaging

Antibodies

Antibodies used	From Abcam:<br>Goat anti-chicken Alexa Fluor 647 (polyclonal; catalog: NC0928213)<br>Anti-Calreticulin-phycoerythrin (clone: FMC 75; catalog: ab83220)<br>Anti-CD3 (clone: SP7; catalog: ab16669)<br>mIgG1 isotype control - PE (clone: B11/6; catalog: ab91357)<br><br>From BD:<br>Anti-hCD4-BUV395 (clone: SK3, catalog: 563550)<br>Anti-hCD8-BUV-805 (clone: SK1, catalog: 612889)<br>Anti-hCD47-BV711 (clone: B6H12; catalog: 563761)<br>Anti-hCD3-BUV737 (clone: SK7; catalog: 612752)<br>Anti-CD11b-BUV395 (clone: M1/70; catalog: 563553)<br>Anti-mCD45-BUV805 (clone: I3/2.3; catalog: 752415)<br>Anti-CD19-BUV496 (clone SJ25C1; catalog: 612938)<br>Anti-hCD62L-BV605 (clone: DREG-56; catalog: 562719)<br>Anti-hCD47-PE (clone: B6H12; catalog: 556046)<br><br>From BioLegend:<br>Anti-hTCR Vβ13.1-APC (clone: H131; catalog: 362408)

Anti-CD11b-APC (clone: M1/70; catalog: 101212)
Anti-mF4/80-BV605 (clone: BM8; catalog: 123133)
Anti-DYKDDDDK Tag (FLAG Tag)-BV421 (clone: L5; catalog: 637322)
Anti-hCD69-BV421 (clone: FN50; catalog: 310930)
Anti-hCD39-BV605 (clone A1; catalog: 328236)
Anti-hTIM3-BV510 (clone F38-2E2; catalog: 345030)
Anti-hCD45RA-BV785 (clone HI100; catalog: 304140)

From Bio X Cell:
Anti-hCD47 (clone: B6H12; catalog: BE0019-1)
Anti-mCSF1R (clone: AFS98; catalog: BE0213)
mIgG1 isotype control (clone: MOPC-21; catalog: BE0083)

From Cell Signaling Technology:
Anti-mF4/80 (clone: D2S9R; catalog: 70076)
Anti-mArg1 (clone: D4E3M; catalog: 93668)

From Jackson ImmunoResearch:
Goat anti-rabbit (polyclonal; catalog: 111-005-008)

From Vector Laboratories:
Goat anti-rabbit (polyclonal; catalog: BP-9100-50)

From Invitrogen:
Anti-hCD45-PerCP-Cyanine5.5 (clone: HI30, catalog: 45-0459-42)
Chicken Anti-c-myc (polyclonal; catalog: A21281)
Goat anti-Chicken-Alexa Fluor 488 (polyclonal; catalog: A11039)
Goat anti-mouse Alexa Fluor 488 (polyclonal; catalog: A11029)
Goat anti-mouse Alexa Fluor 647 (polyclonal; catalog: A32728)
Goat anti-human Alexa Fluor 488 (polyclonal; catalog: A11013)
Goat anti-human Alexa Fluor 647 (polyclonal; catalog: A21445)
Anti-CD47-APC (clone: B6H12; catalog: 17-0479-42)
Fixable Viability Dye eFluor 780 (catalog: 65-0865-14)
Anti-hLAG3-PE (clone: 3DS223H; catalog: 12-2239-42)
Anti-hPD1-PE-Cy7 (clone: J105; catalog: 25-2799-41)
Annexin V Apoptosis Detection Kit - APC (catalog: A35110)

Custom antibodies and proteins:
Sourced from the National Cancer Institute: Anti-14G2a CAR (detection of GD2 and HA CARs; clone 1A7, conjugated to Dylight 650 using Thermo Scientific Dylight 650 Labeling Kit; catalog: 84535)
Sourced from Genscript via custom prep: Anti-CD19 CAR (clone FMC63, conjugated to Dylight 650 using Thermo Scientific Dylight 650 Labeling Kit; catalog 84535)
Sourced from R&D: hB7H3-Fc (catalog: 1027-B3-100) and hHer2-Fc (catalog: 1129-ER-050)
Sourced from Sino Biological: hSIRPa-mFc (catalog: 11612-H38H) and mSIRPa-hFc (catalog: 50956-M02H)
Sourced from the Cochran lab: Hu5F9 and TJC4
Sourced from ALX Oncology: CV-1-Fc

Validation

Custom produced proteins (anti-14G2a-CAR, anti-CD19-CAR, Hu5F9, TJC4, and CV-1) were validated by binding to target protein on T cells and displayed on yeast (for CD47 binders), by comparing antibody specific staining to isotype, secondary only, and unstained controls.

All commercial flow cytometry and immunohistochemistry antibodies were validated by manufacturers at the websites listed below:

Goat anti-chicken Alexa Fluor 647: https://www.fishersci.com/shop/products/goat-anti-chicken-igy-h-l-15/NC0928213
Anti-Calreticulin-phycoerythrin: https://www.abcam.com/products/primary-antibodies/pe-calreticulin-antibody-fmc-75-ab83220.html
Anti-CD3: https://www.abcam.com/products/primary-antibodies/cd3-antibody-sp7-ab16669.html
mIgG1 isotype control - PE: https://www.abcam.com/products/primary-antibodies/pe-mouse-igg1-b116-isotype-control-ab91357.html
Anti-hCD4-BUV395: https://www.bdbiosciences.com/en-us/products/reagents/flow-cytometry-reagents/research-reagents/single-color-antibodies-ruo/buv395-mouse-anti-human-cd4.563550
Anti-hCD8-BUV-805: https://www.bdbiosciences.com/en-us/products/reagents/flow-cytometry-reagents/research-reagents/single-color-antibodies-ruo/buv805-mouse-anti-human-cd8.612889
Anti-hCD47-BV711: https://www.bdbiosciences.com/en-us/products/reagents/flow-cytometry-reagents/research-reagents/single-color-antibodies-ruo/bv711-mouse-anti-human-cd47.563761
Anti-hCD3-BUV737: https://www.bdbiosciences.com/en-us/products/reagents/flow-cytometry-reagents/research-reagents/single-color-antibodies-ruo/buv737-mouse-anti-human-cd3.612753
Anti-CD11b-BUV395: https://www.bdbiosciences.com/en-us/products/reagents/flow-cytometry-reagents/research-reagents/single-color-antibodies-ruo/buv395-rat-anti-cd11b.565976
Anti-msCD45-BUV805: https://www.bdbiosciences.com/en-us/products/reagents/flow-cytometry-reagents/research-reagents/single-color-antibodies-ruo/buv805-rat-anti-mouse-cd45.752415
Anti-CD19-BUV496: https://www.bdbiosciences.com/en-us/products/reagents/flow-cytometry-reagents/research-reagents/single-color-antibodies-ruo/buv496-mouse-anti-human-cd19.612938
Anti-CD62L-BV605: https://www.bdbiosciences.com/en-us/products/reagents/flow-cytometry-reagents/research-reagents/single-color-antibodies-ruo/bv605-mouse-anti-human-cd62l.562719
Anti-CD47-PE: https://www.bdbiosciences.com/en-us/products/reagents/flow-cytometry-reagents/research-reagents/single-color-

antibodies-ruo/pe-mouse-anti-human-cd47.556046
Anti-hTCR Vβ13.1-APC: https://www.biolegend.com/nl-be/products/apc-anti-human-tcr-vbeta131-antibody-15532
Anti-CD11b-APC: https://www.biolegend.com/en-us/products/apc-anti-mouse-human-cd11b-antibody-345
Anti-mF4/80-BV605: https://www.biolegend.com/fr-fr/products/brilliant-violet-605-anti-mouse-f4-80-antibody-8702
Anti-DYKDDDDK Tag (FLAG Tag)-BV421: https://www.biolegend.com/en-gb/products/brilliant-violet-421-anti-dykddddk-tag-antibody-16139
Anti-hCD69-BV421: https://www.biolegend.com/de-de/products/brilliant-violet-421-anti-human-cd69-antibody-7141
Anti-hCD39-BV605: https://www.biolegend.com/de-de/products/brilliant-violet-605-anti-human-cd39-16501
Anti-hTIM3-BV510: https://www.biolegend.com/de-de/products/brilliant-violet-510-anti-human-cd366-tim-3-antibody-12009
Anti-hCD45RA-BV785: https://www.biolegend.com/fr-ch/cell-health/brilliant-violet-785-anti-human-cd45ra-antibody-7972
Anti-hCD47 B6H12: https://bioxcell.com/invivomab-anti-human-cd47-be0019-1
Anti-mCSF1R: https://bioxcell.com/invivomab-anti-mouse-csf1r-cd115-be0213
mIgG1 isotype control: https://bioxcell.com/invivomab-mouse-igg1-isotype-control-unknown-specificity-be0083
Anti-mF4/80: https://www.cellsignal.com/products/primary-antibodies/f4-80-d2s9r-xp-rabbit-mab/70076
Anti-mArg1: https://www.cellsignal.com/products/primary-antibodies/arginase-1-d4e3m-xp-rabbit-mab/93668
Jackson ImmunoResearch Goat anti-rabbit: https://www.jacksonimmuno.com/catalog/products/111-005-008
Vector Laboratories Goat anti-rabbit: https://vectorlabs.com/products/rtu-biotinylated-goat-anti-rabbit-igg
Anti-hCD45-PerCP-Cyanine5.5: https://www.thermofisher.com/antibody/product/CD45-Antibody-clone-HI30-Monoclonal/45-0459-42
Chicken Anti-c-myc: https://www.thermofisher.com/antibody/product/Myc-Tag-Antibody-Polyclonal/A-21281
Goat anti-Chicken-Alexa Fluor 488: https://www.thermofisher.com/antibody/product/Goat-anti-Chicken-IgY-H-L-Secondary-Antibody-Polyclonal/A-11039
Goat anti-mouse Alexa Fluor 488: https://www.thermofisher.com/antibody/product/Goat-anti-Mouse-IgG-H-L-Highly-Cross-Adsorbed-Secondary-Antibody-Polyclonal/A-11029
Goat anti-mouse Alexa Fluor 647: https://www.thermofisher.com/antibody/product/Goat-anti-Mouse-IgG-H-L-Highly-Cross-Adsorbed-Secondary-Antibody-Polyclonal/A32728
Goat anti-human Alexa Fluor 488: https://www.thermofisher.com/antibody/product/Goat-anti-Human-IgG-H-L-Cross-Adsorbed-Secondary-Antibody-Polyclonal/A-11013
Goat anti-human Alexa Fluor 647: https://www.thermofisher.com/antibody/product/Goat-anti-Human-IgG-H-L-Cross-Adsorbed-Secondary-Antibody-Polyclonal/A-21445
Anti-CD47-APC: https://www.thermofisher.com/antibody/product/CD47-Antibody-clone-B6H12-Monoclonal/17-0479-42
Fixable Viability Dye eFluor 780: https://www.thermofisher.com/order/catalog/product/65-0865-14
Anti-hLAG3-PE: https://www.thermofisher.com/antibody/product/CD223-LAG-3-Antibody-clone-3DS223H-Monoclonal/12-2239-42
Anti-hPD1-PE-Cy7: https://www.thermofisher.com/antibody/product/CD279-PD-1-Antibody-clone-eBioJ105-J105-Monoclonal/25-2799-41
Annexin V Apoptosis Detection Kit - APC: https://www.thermofisher.com/order/catalog/product/A35110
hB7H3-Fc: https://www.rndsystems.com/products/recombinant-human-b7-h3-fc-chimera-protein-cf_1027-b3
hHer2-Fc: https://www.rndsystems.com/products/recombinant-human-erbb2-her2-fc-chimera-protein-cf_1129-er
hSIRPa-mFc: https://www.sinobiological.com/recombinant-proteins/human-sirp-alpha-11612-h38h
mSIRPa-hFc: https://www.sinobiological.com/recombinant-proteins/mouse-sirp-alpha-50956-m02h

Eukaryotic cell lines

Policy information about cell lines and Sex and Gender in Research

Cell line source(s)

The Nalm6 B-ALL cell line was provided by David Barrett (Children's Hospital of Philadelphia) and retrovirally transduced to express GFP and firefly luciferase. 143B osteosarcoma cells were acquired from the American Type Culture Collection (ATCC, Manassas) and then retrovirally transduced with human CD19. CHLA-255 neuroblastoma line was obtained and provided by Robert Seeger (Children's Hospital Los Angeles) and retrovirally transduced with GFP and firefly luciferase. MG63.3 was provided by Chand Khanna (National Cancer Institute, National Institutes of Health) and retrovirally transduced with GFP and firefly luciferase. D425 was provided by S. Chesier (Stanford University, Stanford, CA) and retrovirally transduced to express GFP and firefly luciferase. Nalm6 and MG63.3 were originally obtained from ATCC. D425 was originally obtained from Sigma Aldrich. A375 melanoma cells and Jurkat cells (clone E6-1) were obtained from ATCC. The 293GP retroviral packaging line was provided by the Surgery Branch (National Cancer Institute, National Institutes of Health). 293T lentiviral packaging cells were obtained from ATCC. Expi293 protein production cells were obtained from ATCC.

Authentication

Engineered cell lines were verified to express proteins of interest via flow cytometry. All cell lines were previously authenticated via STR fingerprinting prior to use in this manuscript. Cell lines were also authenticated by the commercial vendor, authentication techniques can be found on the vendor website: https://www.atcc.org/ and https://www.emdmillipore.com/.

Mycoplasma contamination

Cells were frequently tested for mycoplasma using the Lonza MycoAlert Mycoplasma Detection kit. All experiments reported in this study used cells that tested negative for Mycoplasma.

Commonly misidentified lines
(See ICLAC register)

No commonly misidentified cell lines were used.

Animals and other research organisms

Policy information about studies involving animals; ARRIVE guidelines recommended for reporting animal research, and Sex and Gender in Research

Laboratory animals

NOD/SCID/IL2Rγ-/- (NSG; NOD.Cg-Prkdcscid Il2rgtm1Wjl/SzJ) male and female mice were used for all in vivo experiments. Mice were

Laboratory animals	6-10 weeks old at the time of tumor or T cell engraftment. Mice were housed at 22 °C and 50 % humidity with a 12 hour light/12 hour dark cycle.
Wild animals	No wild animals were used in this study
Reporting on sex	Findings apply to both sexes
Field-collected samples	No field samples were used.
Ethics oversight	All animal studies were undertaken under Stanford University APLAC-approved protocols. Animals were housed in the Stanford Veterinary Service Center (VSC) Barrier Facility at 22 °C and 50 % humidity with a 12-hour light/12 hour dark cycle. Mice were monitored daily by VSC staff and euthanized if endpoint criteria were met.

Note that full information on the approval of the study protocol must also be provided in the manuscript.

Flow Cytometry

Plots

Confirm that:

☒ The axis labels state the marker and fluorochrome used (e.g. CD4-FITC).

☒ The axis scales are clearly visible. Include numbers along axes only for bottom left plot of group (a 'group' is an analysis of identical markers).

☒ All plots are contour plots with outliers or pseudocolor plots.

☒ A numerical value for number of cells or percentage (with statistics) is provided.

Methodology

Sample preparation	For surface staining: as per methods, cells were washed with FACS buffer (PBS + 2% FBS), stained with fluorophore-conjugated antibodies in FACS buffer (100uL total staining volume per sample) for 30 minutes on ice, washed again with FACS buffer, and then analyzed, or stained with secondary antibody (in 100uL total staining volume) for 30 minutes on ice, before washing again, and then analyzing.
Instrument	BD Fortessa X-20, BD LSR II, BD Accuri, SONY SH800S
Software	FACSDiva ver 8.0.1 (BD Biosciences), BD Accuri software (BD Biosciences), SH800S cell sorter software ver 2.1.5 (SONY), and FlowJo ver 10.8.1 (BD Biosciences)
Cell population abundance	Purity of sorted yeast cells was determined by flow cytometry. Sorted cells were grown up, and run via flow cytometry for binding. Purity of sorted populations was estimated at >90% in all samples.
Gating strategy	Samples were gated on lymphocytes (FSC-A/SSC-A), single cells (FSC-H/FSC-A), and relevant markers (CD47, CAR, CD4, CD8, etc. as specified in the manuscript main text). For cells that were stained with live/dead staining, live cells were also gated into the population of interest (live/dead staining was performed using Fixable Viability Kit eFluor 780 [eBioscience catalog #65-0865-14]). Fluorescence minus one (FMO) and isotype controls were used to determine gating where necessary.<br><br>QuantiBrite-PE (BD Biosciences; catalog 340495) and LegendPLEX Th1 Panel (BioLegend; catalog: 741035) beads were gated following the manufacturer's instructions.<br><br>For in vivo murine blood analysis, samples were gated on lymphocytes and single cells as above, with further gating on human CD45hi cells. CountBright absolute counting beads were used to validate absolute cell numbers.<br><br>Yeast were first gated for forward scatter vs side scatter (FSC-A vs SSC-A), then gated for singlets using forward scatter (FSC-H vs FSC-A). Finally, expressing yeast were identified via c-myc-tag staining or GFP expression, with the c-myc or GFP positive population defined by running uninduced and no-secondary controls. In all cases a clear negative and positive population were observed in induced yeast.

☒ Tick this box to confirm that a figure exemplifying the gating strategy is provided in the Supplementary Information.
